# Recycling Agricultural Waste: Sustainable Solutions for Enhancing Livestock Nutrition

**DOI:** 10.1002/vms3.70321

**Published:** 2025-03-29

**Authors:** Mohsen Kazemi

**Affiliations:** ^1^ Department of Animal Science, Faculty of Agriculture and Animal Science University of Torbat‐e Jam Torbat‐e Jam Iran

**Keywords:** animal health, by‐products, conventional feed, feeding strategy, ruminant

## Abstract

The increasing demand for sustainable agricultural practices prompts a reevaluation of feeding strategies in ruminant nutrition. Agricultural waste, often viewed as a by‐product (BP), presents a promising opportunity to enhance the sustainability of livestock production systems. This review explores the potential of incorporating various agricultural BPs into ruminant diets. Utilizing these BPs reduces the environmental impact of livestock farming and contributes to the circular economy by recycling nutrients back into the food system. The nutritional composition of these wastes varies widely, and their inclusion in ruminant diets improves feed efficiency, animal performance and overall health. Research indicates that specific treatments, such as fermentation and ensiling, enhance the digestibility and nutrient availability of these materials. Moreover, incorporating agricultural waste into ruminant nutrition leads to financial benefits for farmers by reducing reliance on conventional feed sources. However, key challenges remain, particularly the need for further research to optimize inclusion rates and address potential anti‐nutritional factors found in some agricultural wastes. Notably, adding these materials to ruminant diets results in 10%–30% reductions in feed costs and improvements of 5%–20% in key performance metrics, such as weight gain and milk production. These findings highlight the economic and sustainability benefits of utilizing agricultural BPs in livestock feeding practices. This review emphasizes the necessity of developing innovative and sustainable feeding strategies that leverage agricultural waste, calling for interdisciplinary approaches that combine animal nutrition, agronomy and environmental science. By adopting these practices, the livestock sector contributes to food security while minimizing its ecological footprint. Future research focuses on innovative processing techniques, effective management of anti‐nutritional factors, and assessing long‐term impacts on animal health and productivity. Additionally, examining the nutritional and health aspects of commonly used BPs, such as pomegranate, grape, pistachio, saffron, raisin, olive and tomato, is essential for fully understanding their potential in ruminant nutrition and guiding the development of targeted feeding strategies.

## Graphical abstract

The increasing demand for sustainable agricultural practices prompts a reevaluation of feeding strategies in ruminant nutrition. Agricultural waste, often viewed as a by‐product, presents a promising opportunity to enhance the sustainability of livestock production systems. This review explores the potential of incorporating various agricultural by‐products into ruminant diets. Utilizing these by‐products reduces the environmental impact of livestock farming and contributes to the circular economy by recycling nutrients back into the food system. The nutritional composition of these wastes varies widely, and their inclusion in ruminant diets improves feed efficiency, animal performance and overall health. Research indicates that specific treatments, such as fermentation and ensiling, enhance the digestibility and nutrient availability of these materials. Moreover, incorporating agricultural waste into ruminant nutrition leads to financial benefits for farmers by reducing reliance on conventional feed sources. However, key challenges remain, particularly the need for further research to optimize inclusion rates and address potential anti‐nutritional factors found in some agricultural wastes. Notably, adding these materials to ruminant diets results in 10%–30% reductions in feed costs and improvements of 5%–20% in key performance metrics, such as weight gain and milk production. These findings highlight the economic and sustainability benefits of utilizing agricultural by‐products in livestock feeding practices. This review emphasizes the necessity of developing innovative and sustainable feeding strategies that leverage agricultural waste, calling for interdisciplinary approaches that combine animal nutrition, agronomy and environmental science. By adopting these practices, the livestock sector contributes to food security while minimizing its ecological footprint. Future research focuses on innovative processing techniques, effective management of anti‐nutritional factors, and assessing long‐term impacts on animal health and productivity. Additionally, examining the nutritional and health aspects of commonly used by‐products, such as pomegranate, grape, pistachio, saffron, raisin, olive and tomato, is essential for fully understanding their potential in ruminant nutrition and guiding the development of targeted feeding strategies.

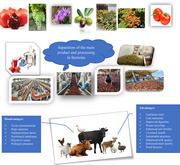

## 1 Introduction

The global demand for livestock products is steadily increasing due to population growth and changing dietary preferences, leading to an urgent need for sustainable agricultural practices. Ruminants, including cattle, sheep and goats, play a crucial role in the agricultural economy by converting fibrous plant materials into high‐quality protein sources for human consumption (Palmonari et al. 2021; Koakoski et al. 2024). However, traditional feeding practices depend on conventional feedstuffs, which can be costly and harmful to the environment. In this context, the utilization of agricultural by‐products (BPs) as feed ingredients presents a promising solution to enhance the sustainability of ruminant nutrition (Cavallini et al. 2021). Agricultural BPs, such as crop residues, fruit and vegetable waste and agro‐industrial BPs, are generated in large quantities worldwide. In 2021, the United Nations estimated that approximately 931 million tons of food waste were produced globally, representing 17% of the total food production worldwide (Thaore et al. 2024). This waste mainly originated from households, retail outlets and the food service industry (Thaore et al. 2024). Additionally, food leftovers are nutritious, highly digestible and safe feed ingredients that can significantly enhance the environmental sustainability of livestock by providing suitable dietary options for both monogastric and ruminant animals (Pinotti et al. 2021). These BPs are often underutilized and disposed of, leading to environmental concerns such as greenhouse gas emissions and soil degradation (Vastolo et al. 2024). The proper utilization of these BPs not only enhances ruminant nutrition but also mitigates environmental impacts by reducing agricultural waste disposal, which, in turn, lessens greenhouse gas emissions and soil degradation. Furthermore, by incorporating these materials into ruminant diets, farmers can not only reduce waste but also improve the nutritional value of their feed, thereby enhancing animal performance and health (Nath et al. 2023; Vastolo et al. 2024). Additionally, the inclusion of botanical supplements and BPs could serve as a viable alternative to antibiotics for promoting growth within the poultry sector (Jalal et al. 2024). Research has shown that agricultural BPs can provide essential nutrients, including fibre, protein and energy, which are vital for ruminant growth and productivity (Akram and Fırıncıoğlu 2019). For example, crop residues, like corn stover and wheat straw, have been demonstrated to be effective feed sources when treated appropriately to enhance their digestibility (Akram and Fırıncıoğlu 2019). Furthermore, the fermentation of fruit and vegetable waste can improve its nutritional profile, making it a viable feed option for ruminants (Sinthuja et al. 2023). A review indicates that when incorporated in appropriate and sufficient quantities, BPs can enhance the quality characteristics of meat, particularly regarding its nutritional, sensory and emotional aspects (Pinotti et al. 2023). The incorporation of BPs, such as distiller's grains and oilseed meals, has also been shown to improve the protein content of ruminant diets, leading to better growth rates and feed conversion efficiency (Nath et al. 2023). However, the literature reports that BPs, mainly distiller's grains, may pose risks related to pesticide and phytotherapeutic residue (Gasparini et al. 2024). Furthermore, research indicates that substituting 11%–16% of energy‐dense feed crops, such as cereals and cassava, with agricultural BPs can save approximately 15.4–27.8 million hectares of land, as well as between 3 and 19.6 km^3^ of blue water and 74.2–137.8 km^3^ of green water for cultivating alternative food crops (Govoni et al. 2023). This approach presents a viable strategy for mitigating the unsustainable exploitation of natural resources, both locally and through the virtual trade of land and water, thereby promoting more sustainable solutions for enhancing animal diets by recycling agricultural waste (Govoni et al. 2023). Despite the potential benefits, the inclusion of agricultural BPs into ruminant diets poses several challenges. Variability in the nutritional composition of these materials, the presence of anti‐nutritional factors and the need for appropriate processing techniques are critical considerations that must be addressed. For instance, certain BPs may contain high levels of tannins or lignin, which can negatively impact digestibility and nutrient absorption. Therefore, a comprehensive understanding of the nutritional value and recommended inclusion proportions of agricultural BPs is essential for developing effective feeding strategies. Moreover, the economic implications of using agricultural BPs cannot be overlooked. The increasing costs of traditional feed ingredients have led farmers to look for alternative sources that are both cost‐effective and environmentally sustainable. Utilizing agricultural BPs can result in substantial economic advantages, which, in turn, enhances the profitability of livestock production (Nath et al. 2023). Additionally, using local agricultural waste can lower transportation costs and decrease the carbon footprint related to feed sourcing. Furthermore, a literature review indicated that rabbit performance, meat quality, immune response and overall health can be influenced by fruit and plant BPs, as well as their essential oils (Abd El‐Aziz et al. 2024). Additionally, animal feeds constitute approximately 60%–70% of the variable production expenses in intensive livestock operations (Salami et al. 2019). Currently, livestock producers are facing reduced profitability, primarily due to rising global prices of traditional feed sources. The incorporation of low‐input feed materials, such as agri‐BPs, has the potential to mitigate feed costs and enhance the economic viability of livestock farmers, particularly in arid regions that heavily rely on imported grains (Salami et al. 2019). Furthermore, the incorporation of agricultural BPs, particularly those containing tannins and phenolic contents, into ruminant diets not only enhances feed efficiency and animal performance but also significantly reduces methane emissions and feed costs, thereby promoting environmental sustainability and contributing to the circular economy in livestock production systems (Manoni et al. 2024, 2023; Khejornsart et al. 2024). This article investigates sustainable approaches for integrating agricultural BPs into ruminant diets, focusing on their advantages, challenges and future research directions. Transforming waste into feed allows the livestock industry to contribute significantly to a more sustainable food system, promoting environmental stewardship while meeting the growing demand for animal protein. Future studies should explore innovative processing methods, such as fermentation and ensiling, to enhance the nutritional value of these BPs and evaluate their long‐term impacts on animal health and productivity. Additionally, this review assesses commonly used BPs in animal feed, including pomegranate, grape, pistachio, saffron, raisin, olive and tomato, with an analysis of recent scientific literature to elucidate their nutritional benefits and health implications for livestock.

## 2 Pomegranate BPs

**FIGURE 1:**
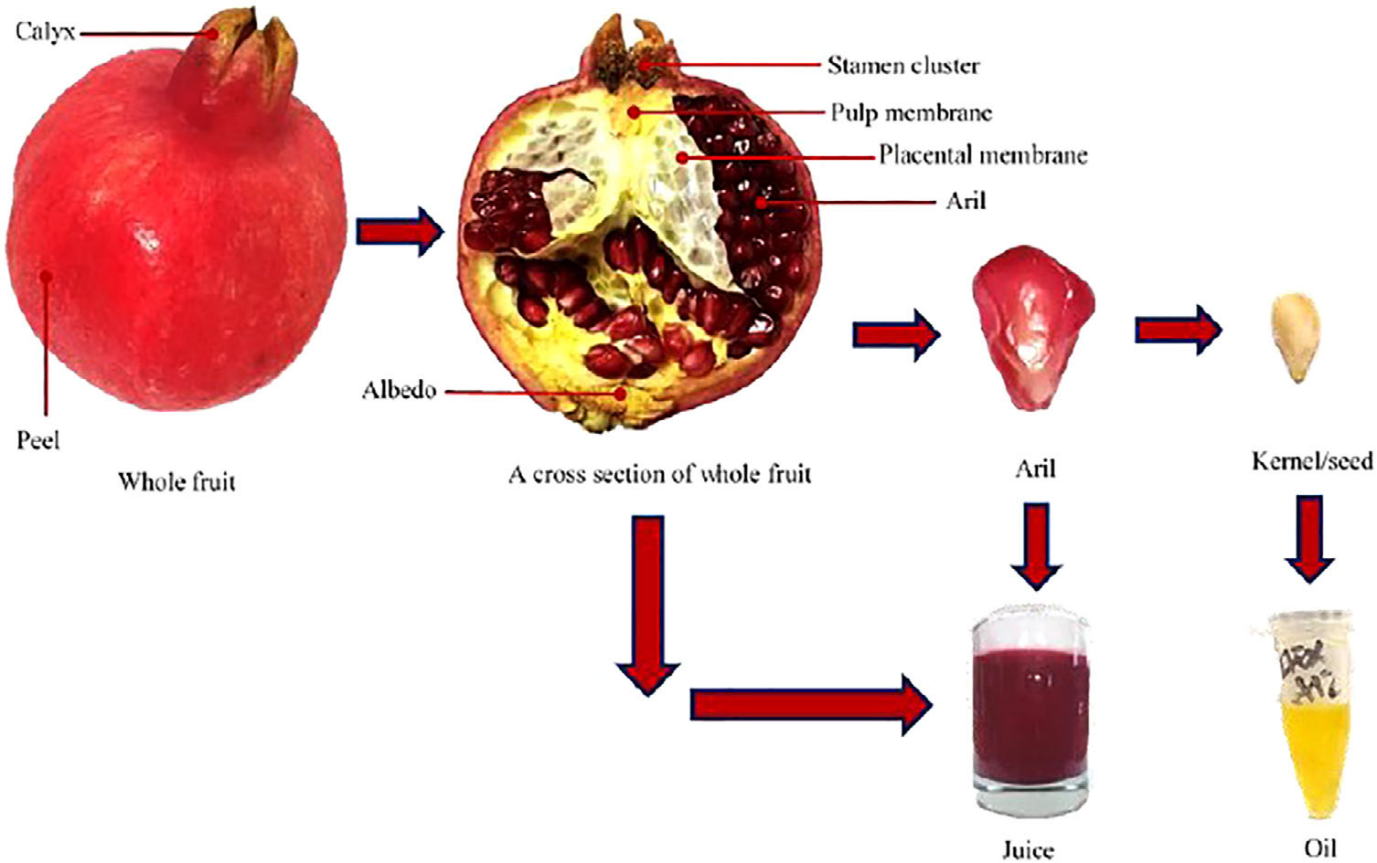
Different parts of pomegranate and its by‐products. *Source*: Derived from Ampem (2017).

The pomegranate, scientifically designated as *Punica granatum* L., is a remarkable species belonging to the family Punicaceae. It can take the form of either a deciduous shrub or a small tree and is widely grown in many different regions, especially in the Middle East, Iran, several European countries and Southeast Asia. This cultivation has garnered significant agricultural and economic importance (El‐Hadary and Taha 2020). Pomegranate fruit is widely used in the fruit processing and beverage industries, especially for creating juices and soft drinks. However, this production process unintentionally creates a significant amount of low‐cost, non‐edible waste derived from fruit, mainly consisting of peels and seeds. Although they are frequently neglected, these BPs are rich in important bioactive compounds (Das et al. 2021). All parts of the pomegranate plant, such as its leaves, stems, fruits, bark and roots, contain a wide range of bioactive compounds. These include a diverse range of phenolic compounds, such as hydrolysable tannins (including pedunculagin, punicalin and punicalagin), as well as ellagic and gallic acids. Additionally, flavonoids like catechins and anthocyanins, along with other complex flavonoids and polysaccharides, are also present (Quideau et al. 2011; Smaoui et al. 2019; Ain et al. 2023). This fruit, commonly referred to as the ‘seeded apple’ or ‘granular apple’, is highly regarded and consumed globally due to its delightful flavour, impressive nutritional profile and numerous medicinal properties that have been well‐documented in various scholarly works (Pathak et al. 2016). These discarded BPs can be repurposed as functional food ingredients, food additives, nutraceuticals and dietary supplements, helping to boost the phenolic content in human diets, which are increasingly acknowledged for their health benefits (Pathak et al. 2016; Gullón et al. 2020). Different parts of pomegranate and its BPs are exhibited in Figure 1. In this figure, various sections of the pomegranate fruit are illustrated, along with the extraction of its juice and the production of oil from pomegranate seeds.

Notably, these bioactive compounds are characterized not only by their natural origins but also by their demonstrated antioxidant and antimicrobial properties. Research has shown that these properties positively influence the quality, safety and longevity of various food products, ranging from oils (El‐Hadary and Taha 2020) to meat (Natalello et al. 2020; Kazemi and Valizadeh 2021; Nemati et al. 2024), fish (Yu et al. 2022) and even dairy products such as cheese, curd and various forms of fermented milk (Kandylis and Kokkinomagoulos 2020). Additionally, a study has confirmed the positive effects of pomegranate macerate on sheep gastrointestinal nematodes (Castagna et al. 2024). Recently, a study focused on reducing the negative effects of pomegranate peel (PP) tannins on fattening lambs by utilizing tannase‐producing bacteria (Chaji and Jahanara 2023). The findings indicated that treating PP with tannin‐degrading bacteria was more advantageous than leaving the raw PP untreated (Chaji and Jahanara 2023). Furthermore, employing these bacteria offers an effective strategy for lowering tannin levels, which enhances the nutritional quality of PP for ruminants (Chaji and Jahanara 2023). In addressing the anti‐nutritional factors present in agricultural BPs, such as tannins and lignin, it is crucial to explore specific solutions that can mitigate their adverse effects. Enzymatic treatments, such as the use of tannase and ligninase, have been shown to effectively reduce the levels of tannins and lignin, thereby enhancing the nutritional value of feed (FAO/IAEA 2000; Grgas et al. 2023). Additionally, microbial fermentation has emerged as a promising approach, where specific strains of bacteria and fungi can degrade these compounds, making nutrients more bioavailable (Salas‐Millán and Aguayo 2024). For instance, the fermentation of BPs using *Aspergillus niger* has demonstrated significant reductions in tannin content, leading to improved digestibility in livestock (Ikusika et al. 2024). These methods not only improve the nutritional profile of the feed but also contribute to the sustainability of livestock farming by utilizing agricultural waste more effectively. A study examined the effects of adding dietary pomegranate pulp to a total mixed ration (TMR) for Ghezel lambs, focusing on growth performance, blood parameters, carcass traits, meat quality and shelf life (Nemati et al. 2024). The findings suggested that adding pomegranate pulp to the diet of Ghezel lambs positively impacts animal performance and meat quality. Specifically, there was an increase in carcass weight, higher concentrations of intramuscular fat and elevated levels of both mono‐ and polyunsaturated fatty acids (MUFAs, PUFAs) (Nemati et al. 2024). Additionally, the study observed higher total unsaturated fatty acid (UFA) content and an improved ratio of polyunsaturated to saturated fatty acids (SFAs) in the meat (Nemati et al. 2024). The use of pomegranate pulp also resulted in lower fat and ash content in the liver, as well as enhancements in the oxidative status (as measured by thiobarbituric acid‐reactive substances) and quality attributes of the meat, including water‐holding capacity, colour and pH (Nemati et al. 2024). Some researchers have suggested that adding 4% pomegranate seed oil to the diet of fattening lambs may improve the levels of specific PUFAs and lower the *n*‐6/*n*‐3 fatty acid ratio in the fat of the carcass (Karampour et al. 2024). This addition appears to have no negative impact on the lambs’ fattening performance, carcass features or the stability of meat colour (Karampour et al. 2024). The findings of a study indicated that supplements of vitamin C, organic selenium, betaine and PP effectively reduce heat stress. Additionally, these supplements positively affect cecal fermentation, the composition of microbiota, antioxidant levels and the overall performance of rabbits (Abu Hafsa et al. 2024). In another study, the impact of water‐extracted PP on ruminal protein degradation and post‐ruminal digestion in dairy cows was assessed (Abarghuei et al. 2020). The inclusion of PP extract reduced the acetate:propionate ratio and ammonia nitrogen production after 24 h of incubation. Additionally, the total number of protozoa, specifically the genera *Dasytricha* and *Isotricha*, along with the subfamilies Entodiniinae, Diplodiniinae and Ophrioscolecinae, decreased with increasing dietary concentrations of PP extract. Higher levels of PP extract were associated with a reduction in the numbers of proteolytic bacteria (Abarghuei et al. 2020). These findings indicate that all levels of PP extract supplementation diminish protozoal populations and lower ammonia nitrogen concentrations. Furthermore, all concentrations of PP extract reduced protein degradation in the rumen, whereas no significant effects on crude protein (CP) degradation were observed in the total digestive tract (Abarghuei et al. 2020). Integrating agricultural BPs, such as pomegranate BPs, into the diet at concentrations between 5% and 40% of DM generally does not negatively impact milk, fat or protein production. This highlights the ability of these feeds to serve as substitutes for concentrates, thereby improving both economic and environmental sustainability and reducing competition for food resources between humans and animals (Correddu et al. 2023). The effects of partially replacing forage and concentrate with pomegranate pulp silage (PPS) and dried pomegranate seed pulp (PSP) on the performance, dry matter intake (DMI) and carcass characteristics of fattening Mehraban lambs were evaluated (Ghoreishi et al. 2021). The results showed that both PPS and PSP are effective alternatives for partially substituting the diet of fattening lambs, leading to lower dietary costs without negatively impacting animal performance (Ghoreishi et al. 2021). In another study, 24 male lambs were chosen to compare the effects of a diet containing PSP at a level of 100 g/kg of dietary dry matter (DM; PSP100) with a control diet that excluded PSP (Obeidat 2023). The results indicated that nutrient intake was significantly higher in lambs fed the PSP100 diet compared to those on the control diet. Additionally, both nitrogen intake and nitrogen retention were greater in the lambs that consumed the PSP100 diet. The final weight, total gain and average daily gain (ADG) were also superior in the lambs receiving PSP100. Both hot and cold carcass weights were significantly higher in the PSP100 group compared to the control group. Furthermore, carcass cut weights increased with the PSP100 diet (Obeidat 2023). The authors concluded that adding PSP to lamb diets positively improved growth and carcass characteristics without negatively impacting the health of the lambs. Therefore, they recommend using PSP as an alternative to traditional feeds in formulated rations for lambs (Obeidat 2023). In another study, the effect of including whole pomegranate BPs (WPB) in the lamb diet on meat flavour was investigated (Natalello et al. 2023). The researchers observed an increase in total PUFAs, as well as levels of vaccenic and rumenic acids, in lambs that received 200 g/kg DM of WPB (Natalello et al. 2023). Additionally, they found that most volatile compounds resulting from lipid degradation, such as aldehydes, alcohols, ketones and hydrocarbons, were present in higher concentrations in the meat from WPB‐fed lambs compared to the control group, with the exception of 2‐pentanone, which was found in greater amounts in the control meat (Natalello et al. 2023). Although the smart nose technology successfully distinguished between the dietary treatments, the consumer panel did not identify any significant differences in meat flavour (Natalello et al. 2023). The incorporation of PP into diets supplemented with fish oil or fat enriched with palmitic acid resulted in enhanced feed intake and antioxidant capacity. However, no interactions were observed concerning oxidative stress and inflammatory markers in dairy cows (Akhlaghi et al. 2022). It was shown that lambs fed a mixture of pomegranate powder and laurel bay leaf powder (LLP) exhibited better performance than those receiving only 1% LLP, without negatively impacting biochemical blood parameters (Abdulzahrah and Al‐Wazeer 2022). A study was conducted to examine the effects of three levels of dietary pomegranate BP extract (PBE) (100, 150 and 200 mg) on the carcass characteristics, growth performance, nutrient digestibility and selected blood parameters of New Zealand White rabbits (Hassan et al. 2020). The findings indicated that supplementing rabbit diets with PBE at concentrations of 100, 150 and 200 mg/kg significantly enhanced both growth performance and nutrient digestibility. Additionally, PBE demonstrated antioxidant and antibacterial effects in the growing rabbits (Hassan et al. 2020). It has been reported that the intensive farming methods used in rabbit production can create environmental problems and compromise animal welfare, especially when inadequate management of agricultural waste occurs (El‐Sabrout et al. 2024). The study examined the effects of dietary pomegranate seed cake (PSC) as a replacement for corn and barley grains on several parameters. These included productive traits, carcass characteristics, the composition of intramuscular and subcutaneous fatty acids and the antioxidant status of the meat (Kotsampasi et al. 2021). The findings suggest that PSC can partially replace cereals in the diets of growing lambs without negatively affecting their performance or quantitative carcass traits. Furthermore, the supplementation of PSC in the diet appears to enhance the nutritional and functional qualities of both meat and subcutaneous fat, as evidenced by an increase in essential fatty acids, particularly *trans*‐10, *cis*‐12 C18:2. Specifically, low levels of PSC inclusion may have a positive influence on the antioxidant potential, as well as the nutritional and functional quality of the meat. However, higher inclusion levels (particularly 235 g/kg of concentrate) may lead to adverse effects (Kotsampasi et al. 2021). Pomegranate leaves have considerable nutritional value for ruminants and can be ensiled with yogurt or molasses to improve their digestive and fermentation properties in both silage and ruminal environments (Kazemi et al. 2022). The techniques of fermentation and ensiling are critical in enhancing the digestibility and nutrient availability of agricultural BPs (Yang et al. 2021). Fermentation, a process involving the microbial conversion of organic substrates, has been shown to significantly increase the bioavailability of nutrients (Knez et al. 2023). For instance, studies indicate that fermentation can improve protein digestibility by up to 25% due to the breakdown of anti‐nutritional factors (Çabuk et al. 2018). Similarly, ensiling, which involves the anaerobic preservation of forage, can enhance the nutritional profile of feed by promoting the growth of beneficial microorganisms such as *Lactobacillus plantarum*, which aids in the fermentation process (Yang et al. 2020; Jin et al. 2024). However, it is essential to address the challenges associated with these processing methods. The costs associated with establishing fermentation or ensiling facilities can be a barrier for many producers, particularly in small‐scale operations. Additionally, scalability remains a concern, as the successful implementation of these techniques often requires specific conditions that may not be feasible in all settings. A balanced view of these methods must consider both their benefits and limitations. To ensure clarity for a broader audience, it is important to define technical terms. The use of pomegranate fruit peel as a substitute for antibiotic growth promoters in poultry nutrition shows significant potential. Research indicates that PP can effectively improve the health and growth of poultry while reducing the side effects associated with the use of antibiotics (Abdelhafez et al. 2015; Hafeez et al. 2023). PP not only supports growth but also enhances immune responses, extends the shelf life of meat, improves egg quality and increases nutrient availability. Additionally, it contributes to better bone quality and reduces the emission of odorous gases from poultry manure, likely due to its abundant antioxidant properties and bioactive compounds such as phenols and tannins (Akuru et al. 2020). Research has demonstrated that incorporating extracts from pomegranate and grape wastes at concentrations of 0%, 2%, 4% and 6% into chilled poultry carcasses can enhance preservation and prolong shelf life, maintaining acceptable quality standards for up to 9 days under suitable storage conditions (Javanmard Dakheli 2020). Furthermore, the use of pomegranate extracts in slaughterhouses may serve as an environmentally friendly, natural and safe decontamination method within a comprehensive food safety system (Javanmard Dakheli 2020). Investigations on the use of pomegranate BPs in animal diets are shown in Table 1. The findings of a study suggested that the inclusion of a blend of *Moringa oleifera* leaf meal and PP powder in quail diets could provide considerable benefits for growth and overall health (Maqsood et al. 2024). The chemical compositions of DM, ash, CP, ether extract (EE), total sugars, neutral detergent fibre (NDF), acid detergent fibre (ADF) and lignin in the peel and seeds of pomegranate were found to be 32.4% versus 20% for DM (peel vs. seed), 3.59% versus 2.47% for ash, 3.80% versus 7.17% for CP, 1.60% versus 1.55% for EE, 42.7% versus 75.3% for total sugars, 26.7% versus 12.8% for NDF, 18.6% versus 8.64% for ADF and 6.80% versus 3.90% for lignin, respectively (de Evan et al. 2024). The metabolizable energy (ME) values for the seeds and peel of pomegranate were reported as 11 and 7.43 MJ/kg DM, respectively (de Evan et al. 2024). Additionally, another study reported the following percentages for moisture, ash, carbohydrates, protein, total fat, crude fibre, total phenolic contents (TPCs) and various mineral contents in PP: 7.27% moisture, 4.3% ash, 66.51% carbohydrates, 3.74% protein, 0.85% total fat, 17.31% crude fibre and TPCs of 18.75 mg/g, with calcium, magnesium, sodium, phosphorus, iron, zinc, manganese and copper measured at 342, 148.64, 64.63, 118.3, 6.35, 0.93, 0.78 and 0.64 mg/100 g, respectively (Azmat et al. 2024). Furthermore, the PSP contained 94.0% DM, 17.8% CP, 34.3% NDF, 20.3% ADF and 11.3% EE (Obeidat et al. 2024). A study conducted by Hagag et al. (2023) identified the contents of CP, moisture, ash and EE as 17.51%, 13.80%, 4.40% and 3.65% of DM, respectively. TPCs in animal feed have been shown to positively impact the health and performance of ruminants (Bešlo et al. 2022). These compounds can enhance the fermentation process in the rumen, leading to improved nutrient utilization and growth performance (Manoni et al. 2023). Moreover, TPC has been associated with a reduction in methane emissions, a significant greenhouse gas produced during ruminant digestion (Manoni et al. 2024, 2023). By modulating the microbial population in the rumen, phenolic and polyphenolic compounds can decrease methanogenic activity, thus contributing to more sustainable livestock production (Teng et al. 2024). Overall, incorporating high levels of TPCs in ruminant diets can lead to both environmental and production benefits.

**TABLE 1:** Investigation on the use of pomegranate by‐products in animal diets.

Pomegranate by‐product	Animal	Offered amount	Finding	Reference
Peel powder	Male Baluchi lambs	5% and 10% of dietary DM	· Increase in plasma gamma‐glutamyl transferase · No alterations in other plasma parameters · Decrease in weight gain	Omidi and Nik (2023)
Seed pulp powder	Awassi ewes and their lambs	7.5% of dietary DM	· Increase in DMI of ewes · Increase in ADG · Increase in total weight gain · Increase in milk yield · Improvements in total solids, protein, fat and lactose content in ewe's milk	Obeidat et al. (2024)
Whole pomegranate by‐product	Lamb	200 g/kg DM of concentrate	· Increase in concentrations of total PUFAs, linolenic, RA and VA in liver and muscle tissues · Detection of punicic acid and three isomers of conjugated linolenic acid in rumen digesta and lamb tissues · Reduction in the C18:1 t10/t11 ratio in rumen digesta, liver and muscle · Suggestion of prevention of the t10‐shift in the rumen biohydrogenation pathway · Improvement of fatty acid composition in the meat	Natalello et al. (2019)
Seed oil	Dairy goat	2.5% of dietary DM or 25 g/kg DM of pomegranate seed oil	· Increase in milk fat concentration and fat/protein ratio · Increase in proportions of VA and C18:1 (*trans*‐9 + *trans*‐10) acids · Increase in rumenic acid concentration within the milk · Increase in both monounsaturated and PUFAs, including *n*‐3 PUFAs · Decrease in the *n*‐6/*n*‐3 PUFAs ratio · Enhancement of conjugated linoleic acid content in milk	Emami et al. (2016)
Peel powder	Brown Swiss dairy cows	5% and 10% of dietary DM	· Linear increase in proportions of C18:2*n*‐6 and C18:3*n*‐3 in milk · Decrease in milk urea nitrogen, blood urea nitrogen and urinary nitrogen excretion · Improvement in fatty acid composition of milk · The addition of 10% peel into the diet resulted in a reduction of metabolic and environmental nitrogen load · Increase in plasma ALT activity	Niu et al. (2023)
Silage (seed and peel)	Dairy cow	60, 120 and 180 g/kg dietary DM	· Improvement in digestibility of NDF and ADF · Increase in production of total ruminal VFAs and molar proportion of propionate · Increase in total milk production and energy‐corrected milk · Decrease in milk protein and lactose content · Increase in lying behaviour · Reduction in feeding costs and decreased ruminal ammonia‐nitrogen production · Improvement of in vitro fermentation parameters	Khorsandi et al. (2019)
Peel powder	Isa Brown laying hens	2.5% and 5% of dietary DM	· Improvements in egg quality characteristics, particularly in yolk · Decrease in yolk MDA levels · Decrease in arachidic acid with 5% peel addition to the diet · Increase in TPCs in the yolk	Lioliopoulou et al. (2023)
Peel powder	Chick	0.2% fermented PP and 0.2% unfermented PP	· Increase in body weight, ADG and villus height/crypt depth ratio of the small intestine by Day 28 · Maintenance of intestinal microbiota in a state favourable to the host · Decreasing unusual changes in the composition of gut microbiota	Xu et al. (2024)
Peel powder	Japanese quail chicks	6% of dietary DM	· Enhanced hepatic expression of the IGF‐1 gene · Improvement in growth performance · Increase in antioxidant properties without adverse influence on carcass quality and haematological parameters	Kamel et al. (2021)
Peel extract	Mice	50 mg/kg/day of total polyphenols	· Increase in energy expenditure · Reduction of liver arachidonic acid content · Increase in antioxidant capacity · Increase in gut microbiota alpha diversity	Duarte et al. (2024)
Seed methanolic extract	Wistar rats	Addition of 500 and 1000 mg to drinking water	· Increase in plasma HDL · Decrease in urea, triglycerides and total cholesterol · Influence on plasma lipid profile and blood glucose · Protection of creatinine and urea	Osman et al. (2024)
Pomegranate supplement (Granatum Plus, *Punicalagina Plus con Pomanox*)	Wistar rats	0.2% (w/v) in drinking water	· Hepatoprotective effect on Wistar rats challenged with fructose · Reduction in adipose tissue accumulation · Improvement of markers of liver injury, including steatosis	Sánchez‐Terrón et al. (2024)
Peel powder	Female rabbits	3% of dietary DM	· 28.5% increase in the number of kits at birth compared to the control group · Increase in birth weight · Increase in plasma haemoglobin content at kit weaning age · Increase in lymph cells and progesterone · Decrease in creatinine and triglyceride levels · Improvement in reproductive efficiency	Hagag et al. (2023)
Peel powder	Rabbit doe	4.5% of dietary DM	· Increase in serum levels of gonadotropic hormones and oestradiol‐17β 2 h post‐mating, on the 20th day of lactation and after weaning · Increase in progesterone levels at mid‐pregnancy · Increase in prolactin levels on the 10^th^ day of lactation · Increase in total DNA, protein concentration, litter size, milk yield and nest traits · Improvements in reproductive performance of does and increase in their antioxidant parameters	Bakeer et al. (2021)

Abbreviations: ADF, acid detergent fibre; ADG, average daily gain; ALT, alanine aminotransferase; DM, dry matter; DMI, dry matter intake; DNA, deoxyribonucleic acid; HDL, high‐density lipoprotein; IGF‐1, insulin‐like growth factor 1; MDA, malondialdehyde; NDF, neutral detergent fibre; PP, peel powder; UFAs, polyunsaturated fatty acids; RA, rumenic acid; TPCs, total phenolic contents; VA, vaccenic acid; VFAs, volatile fatty acids.

Ensiling effectively preserves feed quality by enhancing fermentation, reducing spoilage and improving nutrient availability for livestock. In this regard, co‐ensiling PP with berseem improves silage quality by reducing silage pH, ammonia nitrogen and butyric acid levels and increasing DM and non‐fibre carbohydrates (NFCs) (Ahmed et al. 2025).

## 3 Grape BPs (GBs)

**FIGURE 2:**
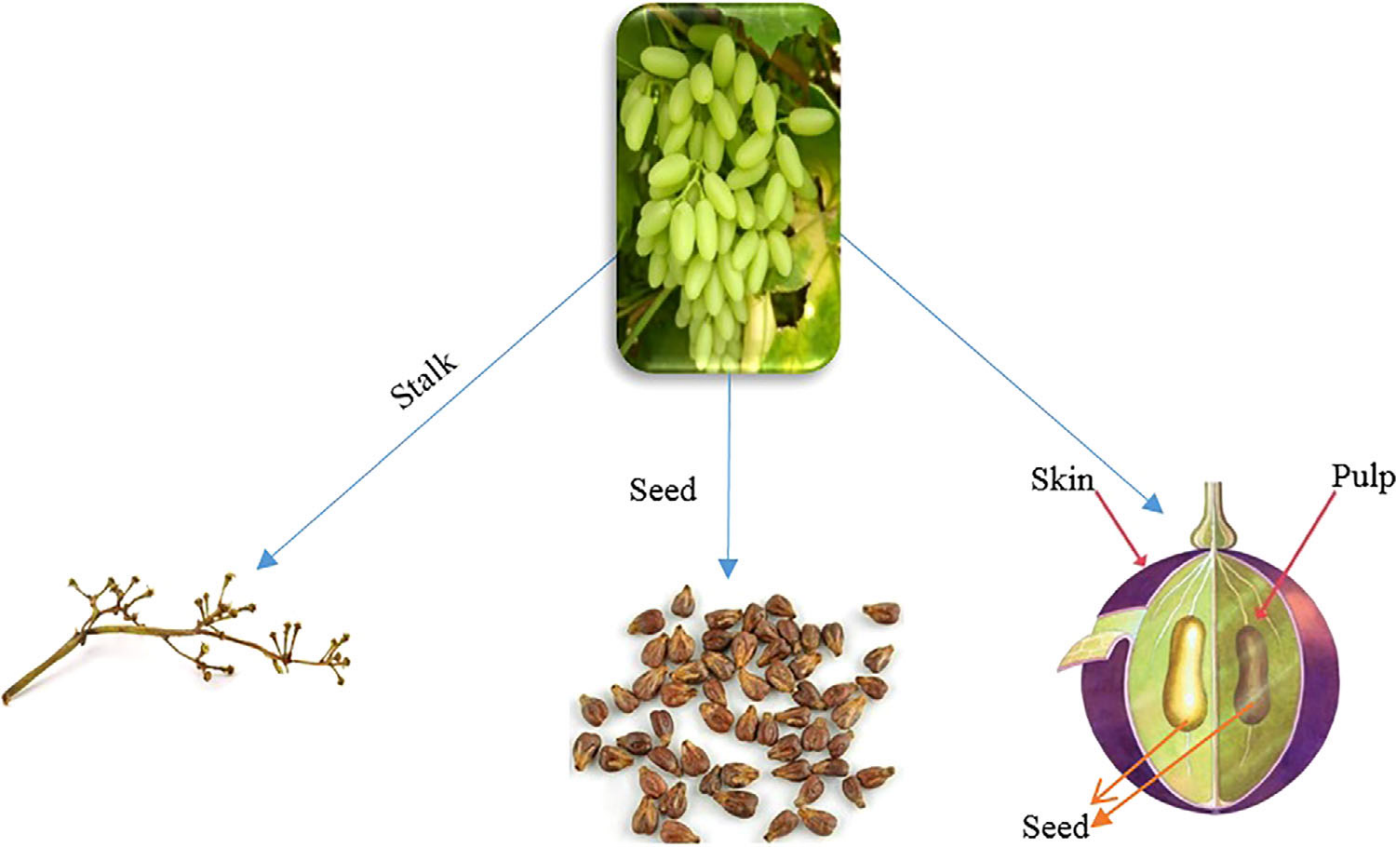
Grape by‐products after juicing.

The grapevine (*Vitis vinifera* L.) is a globally significant agricultural species, widely cultivated for its economic value and diverse product offerings, including raisins, fruit juice, vinegar, seed oils, table grapes and wine (Daler 2024). On the other hand, approximately 75% of global grape production is allocated to the wine industry, with the remaining 25% composed of skins, seeds and stalks, which represent the total weight of grapes utilized in winemaking (Wang et al. 2020; Quagliardi et al. 2024). Seedless pomace accounts for about 48%–62% of total grape pomace (GP), serving as a source of dietary fibre and phenolic compounds, whereas seeds constitute approximately 38%–52%, primarily providing oils rich in UFAs (Beres et al. 2017). A form of GBs after juicing is shown in Figure 2. In this figure, the various parts of the grape fruit, including the pulp, skin, seed and stalk, are highlighted. Each of these components can be considered BPs and utilized in animal feed. The highest concentrations of phenolic compounds, including strong antioxidant, cytotoxic and antibacterial properties, are found in the seeds. In contrast, the skins contain elevated levels of *p*‐coumaric acid hexoside and anthocyanins (Peixoto et al. 2018). Given these factors, GBs can be utilized as dietary supplements, acting as nutraceuticals, foods or ingredients that provide medical or health benefits (Quagliardi et al. 2024). These BPs are abundant in phytonutrients, plant‐derived compounds and, more specifically, phytochemical bioactive plant‐derived compounds linked to positive health outcomes (Frank et al. 2020; Quagliardi et al. 2024). For instance, GP can be utilized to produce extracts with antioxidant properties and as fermentation substrates due to its high content of bioactive compounds demonstrating significant antioxidant activity, including various polyphenols (such as anthocyanins, flavanols, flavan‐3‐ols and procyanidins), phenolic acids, resveratrol and dietary fibre (Caponio et al. 2023). It is one of the most widely cultivated crops, thanks to its many nutritional advantages, which are largely due to its high concentration of polyphenols (Turcu et al. 2021). BPs, like GP, seeds and grape seed oil (GSO), represent valuable but often underutilized resources for ruminants and poultry (Costa et al. 2022). These BPs are rich in fibre and bioactive phenolic compounds, which render them promising alternatives to conventional, unsustainable feed components. Unfortunately, GBs are often discarded or improperly utilized, leading to negative environmental consequences (Costa et al. 2022). Grape seeds make up about 15%–52% of DM in GP and have a complex chemical structure that is affected by various ecological factors, including the conditions of grape cultivation and harvesting time (Teixeira et al. 2014). They generally consist of approximately 40% fibre (mainly cellulose), 16% essential oils, 11% protein and 7% polyphenols (which include flavanols, anthocyanins, phenolic acids and resveratrol) (Antunović et al. 2024). They also contain minerals, sugars and antioxidant compounds like β‐carotene (Antunović et al. 2024). Major minerals found in grape seeds include iron and copper; however, their application in food is limited due to high lignin content, as well as ADF and NDF levels. Additionally, grape seeds are a good source of vitamin E, contributing to their elevated antioxidant properties (Antunović et al. 2024). In poultry nutrition, the effect of adding GBs in feed has been shown to depend on dosage and form. Typically, GBs are included at rates of up to 6%–10% of the feed (DM basis) (Erinle and Adewole 2022). Elevated levels of these BPs may result in substantial concentrations of anti‐nutritional compounds, such as fibre and polymeric polyphenols (e.g., proanthocyanidins), which can impede nutrient digestion and absorption, ultimately affecting weight gain negatively. Conversely, lower inclusion rates (less than 6% feed) have been associated with beneficial bioactive effects, including improved gut morphology, modulation of gut microbiota and enhanced antioxidant activity, primarily due to polyphenol presence (Erinle and Adewole 2022). GBs have been utilized across various livestock species to evaluate their beneficial properties. The most commonly studied animals include dairy cows (Signor et al. 2024), fattening lambs (Elsheikh et al. 2024), lactating goats (Badiee Baghsiyah et al. 2023), beef cattle (Molosse et al. 2023), dairy ewes (Buffa et al. 2020; Bennato et al. 2022), horses (Kolláthová et al. 2020; Kolláthová 2021) and rabbits (Derbali et al. 2024; Romelle Jones et al. 2024).

It was observed that feeding broilers 1%–4% grape seeds enhanced the growth of *Lactobacillus* while decreasing the populations of harmful *Streptococcus* spp. and *Escherichia coli* in the ileum (Abu Hafsa and Ibrahim 2017). Similarly, it was reported that chicks fed 0.72% grapeseed extract exhibited an increase in *Lactobacillus* sp. in their ileal contents (Viveros et al. 2011). Adding grape seeds to the diet (up to 4%) has been found to reduce oxidative stress by boosting the activity of antioxidant enzymes in the plasma, such as superoxide dismutase (SOD) and glutathione peroxidase (GSH‐Px) (Abu Hafsa and Ibrahim 2017). At the same time, it lowers the levels of thiobarbituric acid‐reactive substances (Abu Hafsa and Ibrahim 2017). Overall, the bioactive properties of GBs contribute to the maintenance of intestinal barrier integrity and disease prevention in poultry, notably promoting growth (Erinle and Adewole 2022). For instance, feeding broilers with 2.5% GP increased the relative abundance of beneficial genera like *Bacteroides* and *Lactobacillus* while decreasing the *Firmicutes* to *Bacteroidetes* ratio in the cecum (Erinle et al. 2022). Furthermore, they demonstrated that GBs increased the ratio of intestinal villus height to crypt depth (Erinle et al. 2022). Recent studies have investigated the effects of diets supplemented with grape seed cake (GSC, 5% and 10% of dietary DM) on goat milk quality, blood metabolic profiles and antioxidant status in lactating goats (Antunović et al. 2024). Goats receiving 10% GSC exhibited higher SOD and glutathione reductase activity, along with reduced glucose levels, somatic cell counts and β‐hydroxybutyrate concentrations. It was concluded that 10% GSC supplementation, rich in polyphenols, may alleviate lactation‐related stress, a notably challenging period for livestock (Antunović et al. 2024). The remarkable nutritional profile of white GP (WGP) was emphasized, particularly its significant mineral content, with manganese levels at 106.35 mg/kg (Oancea et al. 2024). Additionally, it has a high concentration of PUFAs at 65.05 g/100 g of fatty acid methyl esters, including a notable amount of omega‐3 fatty acids at 62.66 g/100 g of fatty acid methyl esters (Oancea et al. 2024). The antioxidant compounds present in WGP included 12.49 mg/g of total polyphenols and 3.27 mg/kg of total flavonoids (Oancea et al. 2024). The study also demonstrated the strong antioxidant potential of WGP, as shown by its results in the following assays: 2,2‐diphenyl‐1‐picrylhydrazyl radical‐scavenging activity, measuring 74.26 mM equivalent to Trolox; 2,2′‐azinobis(3‐ethylbenzothiazoline‐6‐sulfonic acid) radical scavenging activity at 75.07 mM equivalent to Trolox and total antioxidant capacity (TAC) at 286.26 mM equivalent to ascorbic acid (Oancea et al. 2024). In another study, researchers examined the effects of adding 100 g/kg of DM from GP silage (GPS) and GP bran (GPB) as alternatives to traditional fibre sources in the diets of steers (Molosse et al. 2024). They focused on carcass traits, meat quality and composition, as well as shelf life. The group receiving GPS exhibited a higher carcass pH compared to the control. Both the GPS and GPB groups demonstrated enhanced oxidative status, characterized by reduced lipid peroxidation and lower concentrations of reactive oxygen species in the meat compared to the control group. On the first day of storage, the activity of the antioxidant enzyme glutathione *S*‐transferase was significantly greater in the meat from the GPS and GPB groups than in the control. Furthermore, the use of GPS was linked to lower bacterial levels in the meat, as evidenced by reduced total coliform counts and a tendency for decreased enterobacteria counts when compared to the control group (Molosse et al. 2024). The dietary treatments also modified the fatty acid profile of the meat; specifically, the GPB diet resulted in an increased concentration of *n*‐6 fatty acids, whereas the GPS diet showed a trend towards higher amounts of both *n*‐6 and *n*‐9 fatty acids. Both diets (GPS and GPB) led to an increase in the concentrations of long‐chain fatty acids (Molosse et al. 2024). Furthermore, the GPS diet resulted in lower levels of SFAs. Their research indicated that dietary interventions utilizing GPS and GPB could serve as promising alternatives for maintaining meat quality standards in real‐world retail situations (Molosse et al. 2024). GP is an abundant source of various polyphenolic compounds, including tannins, proanthocyanidins, anthocyanidins, flavonoids and phenolic acids. The presence and diversity of these compounds are influenced by the conditions, efficiency and chemical reactions involved in the winemaking process (Li et al. 2024). Studies have shown that many of these polyphenolic compounds do not have a negative effect on the growth performance and digestive metabolism of beef cattle (Voicu et al. 2016). Investigations on the use of GBs in animal diets are presented in Table 2. Condensed tannins (CTs) are particularly notable, accounting for 58.7% of the TPCs found in GP (Tayengwa et al. 2020). In a study, the effects of different levels of GP (0%, 10% and 20% of DM from corn silage replaced with dried GP) were examined regarding their impact on growth performance, nitrogen utilization efficiency, antioxidant activity and the microbiota in the rumen and rectum of Angus bulls (Li et al. 2024). The findings revealed that the ADG was higher in the GP0% and GP10% groups compared to the GP20% group, and urinary nitrogen levels decreased linearly with increasing GP addition. In terms of antioxidants, there were higher levels of catalase (CAT) in the GP10% group compared to the GP0% and GP20% groups, and TAC was significantly greater than that observed in the GP20% group. Additionally, an analysis of the microbial network diagram indicated enhanced microbial community complexity and stability in the GP10% group (Li et al. 2024). In another study, the effects of dietary supplementation with dehydrated GP (DGP) at levels of 1% and 2% of dietary DM were evaluated concerning growth performance, nutrient digestibility, nitrogen balance, semen quality, fertility parameters and biochemical metabolic parameters in the blood plasma and seminal fluid of rabbit bucks (Derbali et al. 2024). The 2% DGP supplementation led to improved growth performance and feed intake in the rabbits. However, it enhanced fat digestibility while reducing organic matter (OM) and CP digestibility without affecting nitrogen balance. Notably, 1% DGP improved both sperm mass motility and concentration. The study concluded that adding 1% DGP as a feed additive to the diets of rabbit bucks is an environmentally friendly approach that improves reproductive performance without negatively affecting growth (Derbali et al. 2024). To evaluate the potential of grape stem‐based ingredients for rabbit nutrition, a study assessed its nutritional value, discovering a high fibre content (>40%) and polyphenol levels (>6%) alongside antioxidant and antimicrobial activity against *Staphylococcus aureus* (San Martin et al. 2024). Following this, a feed efficiency trial demonstrated that including up to 10% of grape stem ingredients did not adversely affect rabbit mortality, average daily feed intake, daily weight gain or feed conversion ratios (San Martin et al. 2024). Similarly, the use of GP preserved as silage in the diets of growing–finishing swine was investigated (Giuliani et al. 2024). They reported that the inclusion of GP did not change the proximal composition, cholesterol content, fatty acid profile, juiciness or flavour of the meat. Furthermore, GP improved pork quality characteristics such as increased tenderness, enhanced red colour intensity and delayed lipid oxidation, marking it as a sustainable alternative for utilizing waste products. Grape stems are another BP of winemaking, often discarded or minimally used as soil amendments (San Martin et al. 2024). Nevertheless, they are rich in fibre and polyphenols, making them a promising feed source for livestock. In this context, rabbits are particularly well‐suited for such nutrition because their diets require high fibre levels to prevent digestive issues, whereas polyphenols can contribute to improved animal performance through their antimicrobial and antioxidant properties (San Martin et al. 2024). Bioactive compounds found in GBs have the potential to combat foodborne pathogens, including *Campylobacter jejuni*, *E. coli*, *Listeria monocytogenes*, *Salmonella enterica*, *S. aureus* and *Vibrio cholerae*, as well as microbial toxins such as ochratoxin A and Shiga toxin. These compounds can enhance food microbiological safety, help prevent or treat illnesses in both humans and animals and optimize the utilization of grapes and their BPs (Friedman 2014). In the aquafeed sector, BPs from the wine industry have been investigated as a means to improve feed quality. Their volatile components can enhance sensory attributes and beneficial characteristics, aligning with the principles of a circular economy. Specific compounds, such as hexanoic acid and terpenoids like limonene, may serve as antibacterial, antioxidant and antiproliferative agents. Additionally, esters and terpenoids contribute positively to the aroma of aquafeeds, imparting fruity, sweet, green, fresh and berry notes (Câmara et al. 2020). A study examined the effects of a high‐fat diet (HFD) on lipotoxicity and disturbances in energy metabolism in rat hearts, specifically investigating the protective properties of grape seed extract (GSE) (Majoul et al. 2023). This study highlighted the anti‐lipotoxic and cardioprotective effects of GSE, particularly concerning free fatty acid profiles and energy metabolism enzymes, suggesting its potential as a therapeutic agent for cardiac dysfunction related to obesity. Furthermore, another study explored the therapeutic benefits of GP for treating different stages of non‐alcoholic fatty liver disease in mice (Daniel et al. 2021). The findings indicated that GP reduced food intake, serum leptin levels and body weight gain, as well as mitigating ectopic fat deposition while preserving white adipose tissue mass (Daniel et al. 2021). Additionally, GP improved glucose tolerance and insulin sensitivity, prevented adipose tissue inflammation and decreased hepatic steatosis. The researchers proposed that GP could serve as a beneficial supplement for the prevention and treatment of hepatic steatosis and obesity‐related disorders. In another study, it was reported that silage containing 144 g/kg of viniculture residues enhanced carcass conformation in lambs (Gonçalves et al. 2024). Additionally, the inclusion of GSO positively influenced cow health, particularly enhancing oxidative stability by increasing total thiols, plasma ferric reducing capacity and overall antioxidant capacity (Signor et al. 2024). This finding indicates that adding GSO might enhance the antioxidant defences in cows. A previous research indicated that the seed and pulp fractions of GP exhibit different characteristics, such as cell wall content (523 g/kg DM for seeds vs. 243 g/kg DM for pulp), CP content (104 g/kg DM for seeds vs. 138 g/kg DM for pulp), EE (99.0 g/kg DM for seeds vs. 31.7 g/kg DM for pulp), PUFAs levels (69.6% for seeds vs. 53.3% for pulp) and extractable polyphenols (55.0 g/kg DM for seeds vs. 32.1 g/kg DM for pulp) (Guerra‐Rivas et al. 2016). Additionally, a recent study showed that diets supplemented with grapeseed procyanidins, a type of phenolic compound, improved resilience to weaning stress (Fang et al. 2020). This was evidenced by the increased expression of antioxidant‐related genes like GSH‐Px, SOD and CAT in the liver, as well as lower levels of malondialdehyde (MDA) in the serum, liver and muscle tissues (Fang et al. 2020). Another study indicated that GP and grape seed meal can be added to cattle diets at levels of up to 20% of DM without significantly impacting ruminal fermentation (Khiaosa‐Ard et al. 2023). Furthermore, the study emphasized that grape phenols may have a positive effect on nitrogen metabolism in the rumen, leading to a reduction in ammonia production. Therefore, grape and winery BPs are recommended to be combined with feed ingredients that are high in highly digestible protein (Khiaosa‐Ard et al. 2023). Moreover, it was suggested that using lactic acid bacteria inoculum and zeolite supplementation can improve the quality of GPS for future use in animal feed (Sokač Cvetnić et al. 2023). Overall, similar to other agricultural BPs, grape‐derived materials are abundant in compounds with antioxidant properties beyond the widely recognized resveratrol. These compounds can act as effective scavengers of reactive free radicals or enzyme activators and possess antibacterial, anti‐inflammatory and anticancer properties, among various other health benefits. Moreover, the utilization of grape biomass is gaining traction as a source of dietary fibre and pigments. GBs can be included in the diets of monogastric animals to reduce feeding costs associated with conventional crops like maize and soybean meal, while also enhancing meat quality (Alfaia et al. 2022). Additionally, GBs offer numerous industrial applications, particularly in animal feed, due to their high polyphenol content, which can positively influence intestinal microbiota and morphology and enhance anti‐inflammatory and antioxidant capabilities, thereby promoting intestinal health and production in pigs (Proca et al. 2024).

**TABLE 2:** Investigation on the use of grape by‐products in animal diets.

Pomegranate by‐product	Animal	Offered amount	Finding	Reference
Grape pomace	Churra ewes	75 g/kg dietary DM	· Reduction in plasma lipid peroxidation · Lowering of ruminal TVFAs and ammonia nitrogen concentrations · Induction of milk enrichment in phenolic compounds · No significant impact on milk antioxidant and inflammatory status	Guerra‐Rivas et al. (2016)
Grape pomace	Ewe	10% of dietary DM	· No effect on milk yield and chemical composition · No change in TPCs and AOA · Increase in MUFAs · Decrease in MCSFAs · No modification in total SFAs, PUFAs, SCSFAs and LCSFAs · Decrease in C14 desaturation index · Increase in C18 index · No impact on total caseins and whey protein · Lower κ‐casein content in GP+ milk compared to control milk on Day 60 · Inclusion of 10% GP in diet without modifying milk gross composition but significantly altering fatty acid profile	Bennato et al. (2022)
Grape pomace	Lamb	8% of dietary DM	· Increase in DMI · Enhancement of *Prevotella 1*, *Ruminococcus* 2 and *Sharpea* · *Decrease in acetate‐producing Ruminococcaceae* · Decrease in methane‐producing *Methanobrevibacter* · Stimulation of starch metabolism, intestinal amino acid synthesis and biosynthesis of B vitamins in the rumen · Down‐regulation of colonic methanogenesis	Cheng et al. (2023)
Grape pomace	Lamb	25%, 37.5% and 50% of dietary DM	· Linear negative effect on weight gain and DMI with increasing dietary GP · Increase in carcass cover fat · Improvement in feed conversion ratio · No induction of intoxication in animals receiving GP despite higher copper content · Lower activity of matrix metalloproteinase‐9	Flores et al. (2020)
Grape pomace	Sheep	1% and 2% of dietary DM	· Increase in population of *Methanobrevibacter*, *Butyrivibrio, Fretibacterium* and *Verrucomicrobia _Subdivision3 _genera_incertae_sedis* · Decrease in populations of *Succiniclasticum* and *Selenomonas* · Support for the upregulated pathway of methanogenesis from H_2_ and CO_2_ through significant increase in *Methanobacteriaceae* · Increase in diversity of the rumen bacterial community · Contribution to maintaining rumen bacterial homeostasis	Rolinec et al. (2023)
Grape pomace	Steers	150 g/kg DM of diet	· Increase in DMI · Increased ruminal concentrations of total volatile fatty acids, acetate, isovalerate and acetate to propionate ratio · Reduction in propionate concentrations compared to the control diet · Increase in nitrogen intake, faecal nitrogen, nitrogen retention and nitrogen efficiency utilization · Better potential of GP compared to citrus pulp for replacement with wheat bran	Tayengwa et al. (2021)
Spent grapes	Sow and piglet	16.75% of dietary DM or replacing with 25% of maize	· Using grapes up to 25% in sow and piglet rations · No negative impact on overall performance · Reduction in total feed cost	Tripura et al. (2021)
Grape pomace	Pig	5% of DM	· Increase in proportion of *Lactobacillus delbrueckii*, *Olsenella umbonata* and *Selenomonas bovis* in the cecum · Increase in villus height and villus height/crypt depth ratio in the jejunum · Down‐regulation of pro‐inflammatory cytokines (IL‐1β, IL‐8, IL‐6 and TNF‐α) in cecal tissue · No effect on SCFAs receptors (*GPR41* and *GPR43*) · Improvement in disease resistance potential	Wang et al. (2020)
Grape pomace	Lactating goat	5% of dietary DM	· No effect on DMI · No effect on milk yield and composition · No negative effect on main groups of milk fatty acids · Decrease of rumenic acid in milk fat	Renna et al. (2023)
Grape seed extract	Weaned beef calves	4 g/day	· Increase in ADG · Increase in ruminal contents of microbial protein and butyrate · Increase in DM and NDF digestibility · Increase in serum concentrations of triglyceride, CAT, SOD, immunoglobulin G and immunoglobulin M · Improvement in antioxidant ability and immunity	Ma et al. (2023)
Grape pomace	Dairy cow	7.5% of dietary DM	· Identification of 40 genes affected by GP supplementation · Down‐regulation of genes DNAJA1, *MFF* and *IMPACT* · Positive enrichment of IFN‐α and IFN‐β genes · Positive enrichment of IL6‐JAK‐STAT3 signalling genes · Positive enrichment of complement system genes · Positive enrichment of processes related to protozoan response and negative regulation of viral genome replication · Overall view of blood transcriptomic signature after 60 days of GP supplementation in dairy cows · GP‐induced immunomodulatory effect	Pauletto et al. (2020)
Grape pomace	Dry cow	0.116 kg/cow/day	· GP contains polyphenols and fatty acids · No significant differences in nutrient and fatty acid concentrations between control and experimental groups · Typical nutrient levels in colostrum related to calving time · No effect of GP on nutrient concentrations and fatty acid composition · Positive effect on somatic cell score of colostrum sampled 12 h after calving · GP can be used as a nutrient source for dry cows in small amounts	Rolinec et al. (2021)
Grape seed oil	Rat	25 mg/kg BW	· Improvement in dyslipidaemia and hyperglycaemia in diabetic rats in an experimental model	Shiri‐Shahsavar et al. (2023)
Grape pomace	Rabbit	20% of dietary DM	· No significant differences in live weights · Impaired feed conversion rate, carcass weight and yield in GP group · Higher intramuscular fat percentage in GP · Improved polyunsaturated/SFAs ratio in GPD · Better atherogenicity and thrombogenicity indexes in GP · Higher *n*‐6/*n*‐3 ratio in GPD · Lower total volatile basic nitrogen in GPD meat · Delayed spoilage in GP meat · No improvements in TPCs, antioxidant capacity, reducing power and lipid oxidation in meat · No phenolic compounds detected in meat samples	Bouzaida et al. (2021)

Abbreviations: ADG, average daily gain; AOA, antioxidant activity; BW, body weight; CAT, catalase; DM, dry matter; DMI, dry matter intake; GP, grape pomace; GPD, grape pomace diet; LCSFAs, long‐chain saturated fatty acids; MCSFAs, medium‐chain saturated fatty acids; MUFAs, monounsaturated fatty acids; NDF, neutral detergent fibre; PUFAs, polyunsaturated fatty acids; SCFAs, short‐chain fatty acids; SCSFAs, short‐chain saturated fatty acids; SFAs, saturated fatty acids; SOD, superoxide dismutase; TPCs, total phenolic contents; TVFAs, total volatile fatty acids.

Ensiling enhances the fermentation process by encouraging beneficial microbial activity, which leads to improved nutrient preservation and overall feed quality for livestock. In this regard, a study showed that ensiling GP, particularly with the addition of *Lactiplantibacillus plantarum*, enhances in vitro digestion efficiency by improving nutrient degradation and reducing inhibitory polyphenolic compounds (De Bellis et al. 2022).

## 4 Pistachio BPs (PBs)

**FIGURE 3:**
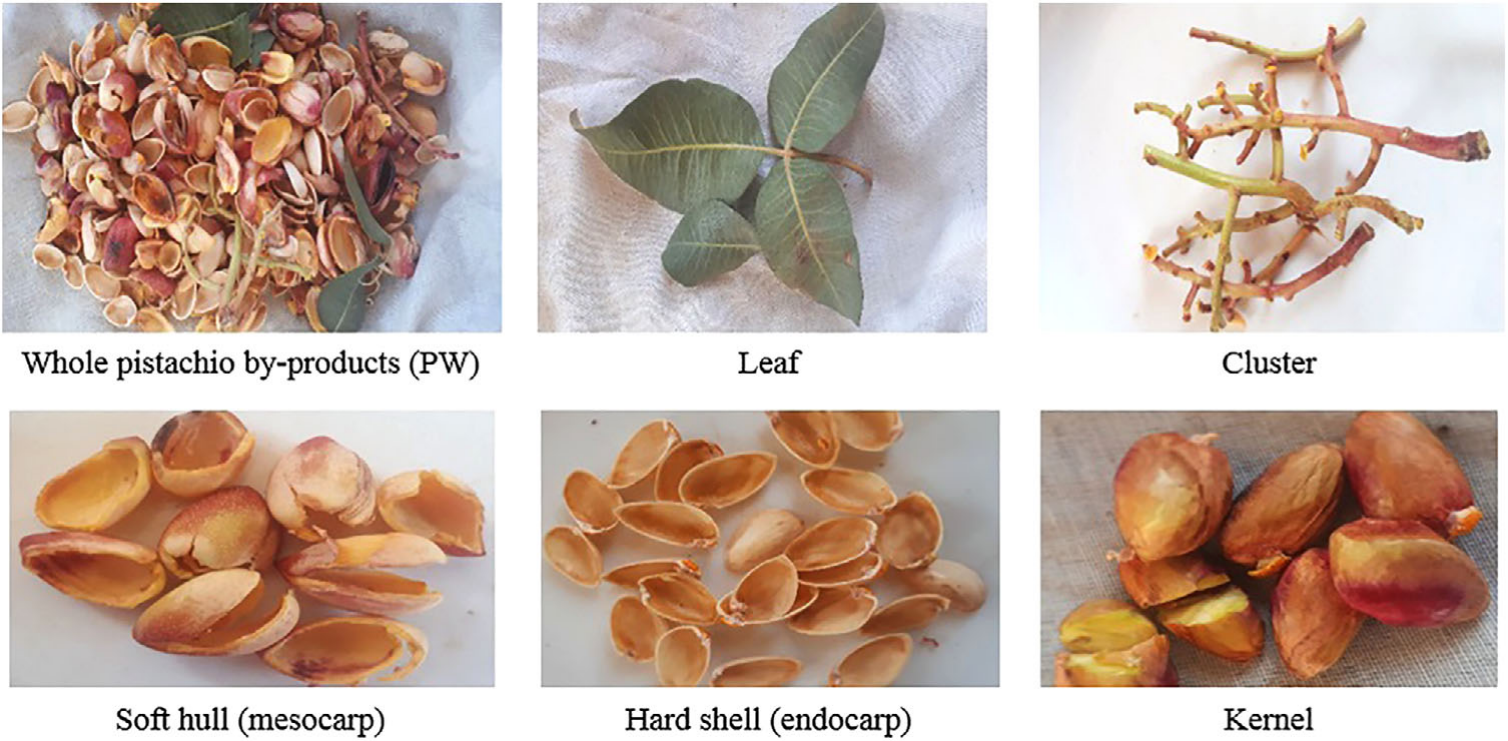
Photos of pistachio by‐products and its derivatives. *Source*: Derived from Kazemi et al. (2024).

Nuts, including pistachios (*Pistachia vera* L.), have long been valued for their nutritional and functional properties. Pistachios are commonly grown and highly valued for their distinctive taste and unique composition. These characteristics make them ideal for use as food ingredients and for oil production (Özdikicierler and Öztürk‐Kerimoğlu 2023). Conventional oil extraction techniques, especially solvent extraction, often necessitate refining processes that can diminish bioactive compounds. As a result, supercritical fluid extraction provides a superior option for maintaining nutritional quality. Notable BPs from pistachio oil production include flour, hulls and extracts, which are rich in terpenoids, phenolic compounds, lipids, amino acids and carbohydrates (Özdikicierler and Öztürk‐Kerimoğlu 2023). Additionally, ground pistachio shells (PSs) can serve as dye adsorbents in water treatment (Özdikicierler and Öztürk‐Kerimoğlu 2023). On the other hand, every year, a significant quantity of PBs accumulates in processing facilities, often discarded in the vicinity of pistachio orchards (Kazemi et al. 2024). Photos of PBs and its derivatives are illustrated in Figure 3. In this figure, the various parts of the pistachio, including the leaves, cluster, hard shell, soft shell and broken kernels, are presented. Each of these components can be incorporated as BPs in animal feed. These BPs typically remain moist, despite their rich nutrient content, creating an ideal environment for microbial growth. This situation can lead to environmental issues and an increase in fungal contaminants around pistachio orchards (Kazemi et al. 2024). During pistachio processing, two BPs are generated: pistachio green hulls (PGH) and PS, which are removed during de‐hulling and shelling, comprising over 75% of the harvested crop. PGH is particularly rich in bioactive compounds, notably phenolics, but is often discarded as waste, creating environmental concerns (Ripari Garrido et al. 2024). Studies have demonstrated its significant antioxidant, antimicrobial and antimutagenic properties. Pistachio nuts primarily consist of lipids (47.5%–57%), followed by protein (17.1%–27.1%), with dietary fibre ranging from 8.6% to 15.3%. Of this dietary fibre, about 97% is insoluble (Ripari Garrido et al. 2024). The kernels have a low moisture content (3.3%–5.34%) and an ash content of approximately 3%. Triglycerides dominate the lipid fraction, with oleic, linoleic and α‐linolenic acids as the main UFAs. Potassium is the primary mineral, particularly abundant in Iranian pistachios (1589 mg/100 g), whereas sodium content is notably low (between 8.72 and 26.4 mg/100 g). Pistachios also contain lipophilic vitamins (A and E) and water‐soluble vitamins, including vitamin C and several B vitamins such as pyridoxine (B6), thiamine (B1) and riboflavin (Ripari Garrido et al. 2024). Research indicates that PB contains a substantial amount of NFC (36.9%–40.4%), moderate levels of CP (11.4%–13.0%) and NDF (30.9%–33.3%), along with being abundant in polyphenolic and tannin compounds (Hosseini Ghaffari et al. 2013a, 2013b; Shakeri et al. 2014). Due to its cost‐effectiveness and availability, PB serves as a preferred source of energy and protein for ruminants (Shakeri 2016). Recently, different amounts of pistachio waste (PW) have been added to the diets of sheep (Ebadi and Mahdavi 2023; Hajalizadeh and Dayani 2021), dairy cows (Mokhtarpour et al. 2012), growing calves (Shakeri et al. 2014), broiler chickens (Ahmadi Kohanali et al. 2022) and Saanen dairy goats (Hosseini Ghaffari et al. 2013a, 2013b; Kordi et al. 2022). However, the use of PW for its phenolic and tannin content in ruminant diets is limited (Bohluli et al. 2007). The TPCs in dried PB have been reported to vary between 7.5% and 14.2%, depending on the variety and growth stage (Bohluli et al. 2007; Bagheripour et al. 2008). Tannins, which are complex polyphenolic compounds, can have either beneficial or harmful effects on ruminants, depending on factors such as consumption method, chemical structure, molecular weight or the physiology of the consuming animals (Hagerman and Butler 1991).

High concentrations of tannins (>50 g/kg DM) in lamb diets can reduce DMI due to their astringent properties and lower nutrient digestibility (Lima Júnior et al. 2010; Mazza et al. 2020). Polyethylene glycol (PEG) is a synthetic, high molecular weight polymer that is poorly digested and absorbed in animal intestines (Grosell and Genz 2006). It is widely utilized as a raw material in the food and pharmaceutical sectors (He et al. 2019). Due to its strong affinity for tannins, PEG can form a complex with tannins (Makkar et al. 1995). Aluminosilicate (AS), a porous clay composed of aluminium oxide and silicon dioxide, has a high specific surface area that can absorb various cations, including ammonium, and is used in treatments for numerous gastrointestinal issues (Herremans et al. 2018). Several methods have been used to reduce the negative effects of phenolic and tannin compounds in ruminant diets, including electron irradiation (Fatehi et al. 2020), gamma irradiation (Valizadeh et al. 2019), ensiling (SoltaniNezhad et al. 2016; Hajalizadeh and Dayani 2021) and the use of materials like PEG (Kordi et al. 2022). Additionally, several management strategies have been suggested to alleviate the impact of tannins, including drying, crushing, chemical treatments and the use of binding agents such as PEG and polyvinyl pyrrolidine (Bagheripour et al. 2008). It has been observed that bentonite clay can act as an effective inactivating agent because it is less expensive than PEG and has the capability to absorb or bind anti‐nutritional factors, such as tannins, in animal feed (Kemboi et al. 2023). Recently, a study was conducted on PWs to examine their chemical and mineral compositions, along with the nutritional impacts of incorporating AS and PEG into PW‐based diets (Kazemi et al. 2024). In the first experiment, the chemical compositions and ruminal fermentation activities of PW and its derivatives were determined. In the second experiment, 40 male Mahabadi goats were divided into 4 groups: a control group, a group with 40% PW, a group with PW plus 10 g of PEG and a group with PW plus 10 g of AS. The results indicated that adding PW to the diet reduced DMI, final body weight and growth performance; however, the addition of PEG or AS significantly improved digestibility and ruminal fermentation activities, enhancing growth performance. It is recommended to include PW at a level of 40% in the diets of fattening goats, supplemented with AS or PEG for optimal nutritional benefits (Kazemi et al. 2024). In another study, the effects of pistachio hull (PH), a tannin‐rich BP, on nitrogen metabolism, milk production and digestibility in lactating dairy cows were examined (Sadeghi et al. 2024). They utilized a 4 × 4 Latin square design involving 12 Holstein cows to evaluate diets that included either soybean meal or slow‐release urea (SRU) in combination with PH. Their results showed that feeding diets with 7.65% PH reduced milk yield, milk urea nitrogen and milk efficiency, while increasing milk fat and protein concentration. The inclusion of PH led to a reduction in the digestibility of DM, CP and NDF. Although there was no significant impact on nitrogen intake and urine or milk excretion, PH increased faecal nitrogen excretion and reduced apparent nitrogen efficiency (Sadeghi et al. 2024). They demonstrated that replacing sugar beet pulp with PH negatively impacted nutrient digestion and milk production efficiency. In contrast, the use of SRU was shown to be a viable nitrogen source that did not affect lactation productivity (Sadeghi et al. 2024). Investigations on the use of PBs in animal diets are presented in Table 3. The study examined how incorporating 6% pistachio skin into the diets of grower and finisher rabbits affects the quality of their meat (Attard, Liotta, et al. 2024). In the study, 150 Martini rabbits were divided into two groups: One group received a diet containing pistachio skins, whereas the other did not. The findings indicated no negative impact on growth or carcass characteristics. However, the treated group showed an enhanced fatty acid profile, characterized by higher levels of monounsaturated and PUFAs, whereas SFAs were reduced. This suggests that pistachio skin could potentially enhance the nutritional value of rabbit meat (Attard, Liotta, et al. 2024). Furthermore, another study compared the nutritional value of PS powder to soybean hulls in pig diets (Kim et al. 2024). In two experiments involving gestating and lactating sows, they measured the apparent total tract digestibility (ATTD) of DM, gross energy (GE) and total dietary fibre (TDF), as well as digestible energy (DE). Their results showed that PS powder had significantly lower ATTD of DM, GE and TDF, as well as lower DE, compared to soybean hulls (Kim et al. 2024). Consequently, the study suggests that the inclusion of PS powder in lactation diets should be limited due to its lower energy content (Kim et al. 2024). One of the primary BPs of pistachio processing is the green hull, which makes up over 60% of the total PBs (Arjeh et al. 2020). The PGH is rich in bioactive compounds, particularly phenolics (Rafiee et al. 2018). A study reported the composition of PGH as follows: DM 23.0%, 11.0%, crude fibre 15.0%, ash 12.0%, crude fat 6.0% and nitrogen‐free extract 55.50% (Shakerardekani and Molaei 2020). Another study investigated the effects of PGHs aqueous extract (PHE) on Ross 308 broiler chicks that were challenged with *Eimeria* (Noruzi et al. 2024). They found that increasing PHE levels improved growth performance, antioxidant capacity, and reduced excreta oocyte counts and lesion scores in challenged broilers. PHE also mitigated the negative effects of *Eimeria* on antioxidant levels, leading to an increase in TAC and enzyme activity. Overall, they noted that PHE enhanced performance and health without causing any adverse effects on intestinal morphology (Noruzi et al. 2024). Furthermore, another study examined the effects of aerobic exercise and pistachio soft hull extract (PSHE) on inflammatory gene expression in the soleus muscles of rats that were fed an HFD (Barshan et al. 2024). The study found that the HFD led to a significant decrease in IL‐6 and an increase in IL‐1β expression. Both aerobic exercise and PSHE, alone or in combination, significantly reduced IL‐1β levels and increased IL‐6 expression compared to the HFD control group. Overall, the findings suggest that aerobic exercise and PSHE can mitigate HFD‐induced inflammation in skeletal muscle (Barshan et al. 2024).

**TABLE 3:** Investigation on the use of pistachio by‐products in animal diets.

Pomegranate by‐product	Animal	Offered amount	Finding	Reference
Shell	Awassi sheep	5% of dietary DM	· Significant reduction in alanine, citrulline, glutamine, glutamic acid, glycine, leucine, ornithine and alpha‐aminoadipic acid in supplemented groups · The group receiving PS exhibited increases in argininosuccinic acid, gamma‐aminobutyric acid, beta‐alanine and sarcosine levels · There was no significant effect on the ratio of short, medium and long‐chain fatty acids in the milk · Significant changes in milk amino acid profiles with 5% inclusion of PS in sheep diets · Importance of considering the benefit‐harm relationship in the context of altered milk amino acid profiles	Kahraman et al. (2023)
Ensiled pistachio residues	Sheep	33%, 66% and 100% of dietary DM	· No significant difference in meat traits among groups; lower fat content in EPR treatments compared to control, with the lowest in the 66% EPR group · Increase in zinc and iron content in mutton following EPR addition, with the highest levels observed in the 100% EPR diet. Improvement in quality and nutritional value of mutton with EPR supplementation compared to standard diet	Ebadi and Mahdavi (2023)
Pistachio external hull polysaccharides (PHP)	Male Wistar rats	10, 25, 50, 75 and 100 mg/kg of body weight	· Study focused on extraction and benefits of PHP · PHP demonstrated protective effects against CCl_4_ and cisplatin‐induced hepatotoxicity and nephrotoxicity in rats · Recovery of plasma biomarkers associated with hepatotoxicity included reductions of 23.23% in AST, 98.97% in ALT and 81.39% in LDH · Normalization of nephrotoxicity biomarkers: creatinine (17.5%), urea (14.59%) and uric acid (14.81%) reductions · PHP improved lipid profile alterations caused by CCl_4_ · Significant reduction in lipid peroxidation (50% and 67.56%) in liver and kidney tissues, indicating reduced oxidative stress · Increases in antioxidant enzymes: SOD (19.89%), GSH‐Px (31.15%) and CAT (27.57%) compared to CCl_4_ treatment; higher enhancements against cisplatin · Histopathological examination supports the protective role of PHP in liver and kidneys · PHP's monosaccharides recognized as potential bio‐antioxidants for pharmaceutical applications	Hamed et al. (2021)
Pistachio green hull	Broiler Chickens	1.5%, 3% and 5% of dietary DM	· Significant reduction in feed conversion ratio during both the initial and entire rearing periods with pistachio skin addition · Addition of 3% PH resulted in significantly higher body weight compared to control and 5% treatment · Increased total antibody titre and immunoglobulin G levels against sheep red blood cells with PH inclusion · Decreased serum cholesterol and LDL concentrations compared to control · A 3% inclusion level resulted in increased height of jejunum villi, decreased width and enhanced relative weight of organs (breast, heart and bursa of Fabricius), along with reduced abdominal fat percentage · Supplementation with green pistachio skin enhanced functional traits, with an optimal level of 3% leading to cholesterol reduction, improved conversion rates and strengthened immune responses	Ahmadi Kohanali et al. (2022)
Pistachio seed coat	Kermani sheep	5, 10 and 15% of dietary DM	· Results showed that feed intake, nitrogen intake, excreted nitrogen and nitrogen retention were not significantly affected by the diets · The addition of PSC decreased ruminal ammonia nitrogen levels without impacting ruminal fluid pH at various time points after feeding · Total protozoa population was unchanged, but cellulolytic species’ population increased linearly · No significant differences in total purine derivatives or microbial protein synthesis across treatments · PSC can be included up to 15% of DM in sheep diets, substituting for wheat bran or other ingredients	Esmaili et al. (2021)
Pistachio by‐product biochar	Lamb	1% of dietary DM	· Inclusion of biochar did not affect DMI but improved ADG and FCR · Higher ruminal ammonia nitrogen observed in lambs fed PBB compared to control · No significant changes in pH, number of rumen protozoa, total VFAs or individual VFAs proportions across treatments · Increased concentrations of allantoin, xanthine plus hypoxanthine and total purine derivatives, along with enhanced microbial nitrogen supply · Biochar serves as a cost‐effective feed additive for improving ruminal fermentation and enhancing animal performance in fattening lamb diets	Mirheidari et al. (2020)

Abbreviations: ADG, average daily gain; ALT, alanine aminotransferase; AST, aspartate transferase; CAT, catalase; DM, dry matter; DMI, dry matter intake; EPR, ensiled pistachio residue; FCR, feed conversion ratio; GSH‐Px, glutathione peroxidase; LDH, lactate dehydrogenase; LDL, low‐density lipoprotein; PBB, pistachio by‐products biochar; PH, pistachio hulls; PHP, pistachio external hull polysaccharides; PS, pistachio shell; PSC, pistachio seed coat; SOD, superoxide dismutase; VFAs, volatile fatty acids.

Ensiling not only improves the stability of feed by minimizing spoilage factors but also enhances the overall nutritional profile, making it a valuable practice in animal nutrition management. Therefore, a study reported that ensiling PBs significantly reduces the levels of CTs, hydrolysable tannins and fibre, thereby decreasing in vitro gas production and OM digestibility (Bagheripour et al. 2008).

## 5 Saffron BPs

**FIGURE 4:**
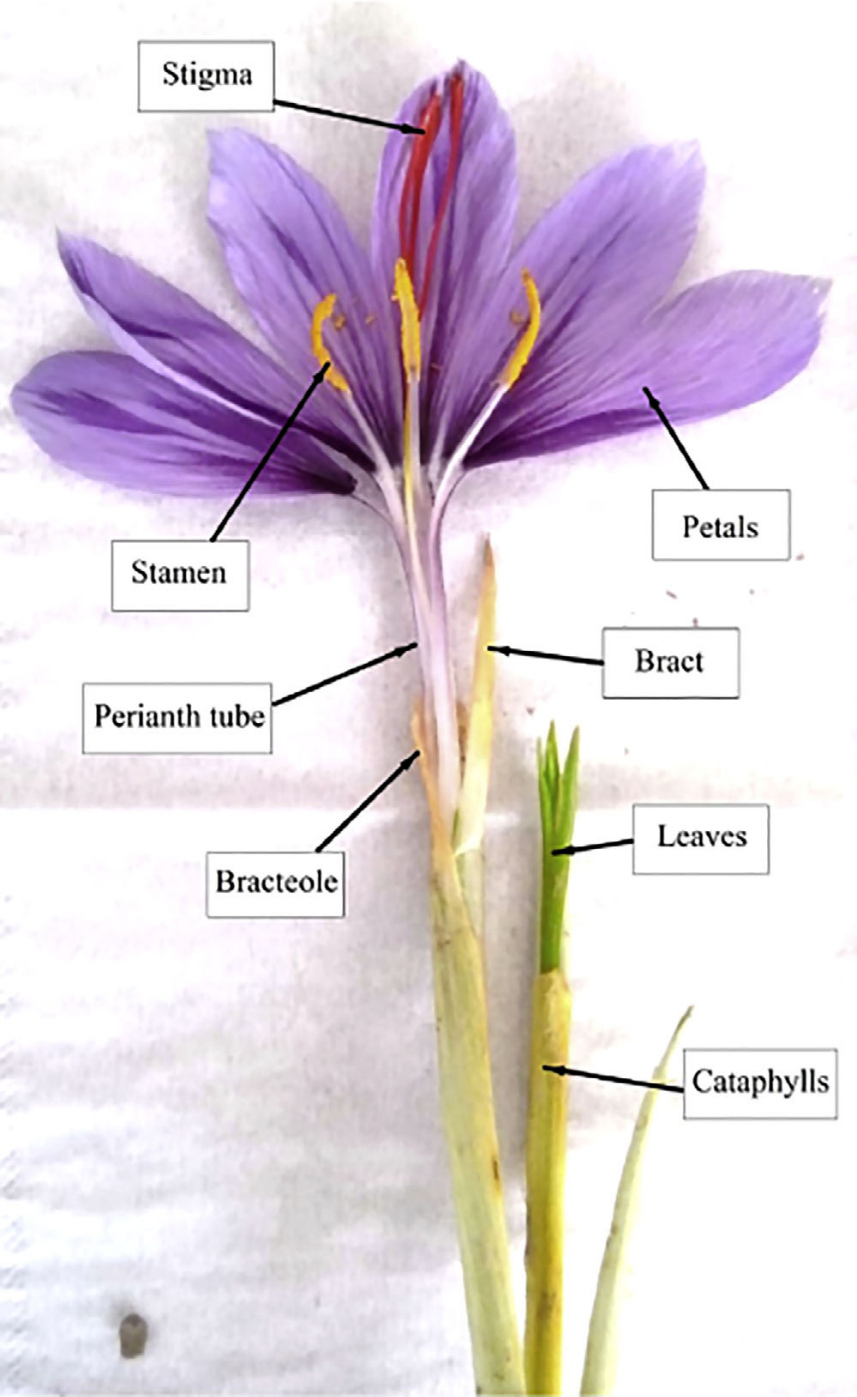
The various parts of the saffron flower. *Source*: Derived from Rashed‐Mohassel (2020).

*Crocus sativus* L. (CC), widely recognized as saffron, is a highly valued spice originating from Asia, particularly from Iran, which is the leading producer of this spice. Saffron, derived solely from dried stigmas, is recognized as the most expensive spice in the world (Marrone et al. 2024). Iran and several countries in the Middle East experience arid and semi‐arid climates. Due to significant constraints on water resources in the region and predictions of future droughts, a shortfall in forage resources previously cultivated through irrigation is anticipated. CC is an autumn‐blooming plant primarily cultivated for its red flower stigmas that, upon drying, produce saffron, often referred to as ‘red gold’ due to its high value (Popović‐Djordjević et al. 2021). With growing concerns about synthetic antioxidants in food, researchers are increasingly investigating the antioxidant properties of saffron's bioactive compounds, including crocin, crocetin, picrocrocin and safranal. These compounds exhibit significant antioxidant potential, demonstrated through various assays (Popović‐Djordjević et al. 2021). Moreover, saffron processing waste and specific plant parts are emerging as valuable sources of bioactive compounds, highlighting the importance of waste valorization in the agri‐food sector and its role in reducing environmental pollution (Popović‐Djordjević et al. 2021). Therefore, identifying and utilizing agricultural waste that can partially meet livestock feed requirements is vital. The use of these unconventional feed sources can not only reduce feed costs but also alleviate the environmental issues associated with their accumulation. Significantly, the primary and notable BPs identified in this region consist of the petals, stamens (anthers) and stem (perianth tube) of saffron. Additionally, saffron is a monocotyledonous plant belonging to the Iridaceae family. Botanically, it completes its life cycle within a year, but agronomically, it is regarded as a perennial plant (Fallahi and Mahmoodi 2018). The presence of compounds such as crocin, picocrocin and safranal in saffron has made it a popular natural colouring and flavouring agent in various industrial and food products (Kaveh and Salari 2018). The various parts of the saffron flower are illustrated in Figure 4. In this figure, the various parts of the saffron plant, including the stigma, petals, stamen, perianth tube, bract, leaves, bracteole and cataphylls, are displayed. The stigma is the most valuable part of saffron, known for its numerous nutritional applications, particularly as a food seasoning, whereas the remaining parts have the potential for use in animal nutrition. Furthermore, investigations on the use of saffron BPs in animal diets are shown in Table 4. Currently, Iran is the largest producer of saffron in the world. Specifically, the production of saffron in Iran reached approximately 336,000 kg in the year 2016, and in 2017, this figure rose by 12% to exceed 376,200 kg (Agricultural Statistics 2017). The total saffron cultivation area in the country in 2017 was equivalent to 1,080,864 hectares, with Razavi Khorasan province holding the largest share of 84,226 hectares (Agricultural Statistics 2017). The weight percentages of the components of fresh saffron flowers include 86.42% petals, 5.93% anthers (stamen) and 7.64% stigma (Hemmati Kakhki 2001). In Iran, after the stigma is separated from the flower, it is dried and sent to the market; however, the remaining saffron flower BPs, including the petals, stamen and stem (perianth tube), are left as main BPs, amounting to approximately 194,445 t of sepals and petals and 13,753 t of anthers annually (Hemmati Kakhki 2001). Occasionally, a small quantity of stigma, leaves, cataphylls, bract, perianth tube and bracteole may be present in this waste, potentially introduced during the harvesting of saffron flowers. Although these residues contain significant amounts of valuable compounds, they are currently disposed of in the environment without utilization. Feeding livestock with these residues can prevent the wastage of these valuable BPs. Saffron petals (SPs) present a relatively suitable potential for livestock nutrition, with a reported CP concentration of 11.55% on a DM basis, and the rapid degradation component of the DM of SP being reported as 65.94% (Alipour et al. 2016). In another study, the concentrations of sodium, potassium, calcium, copper, iron, manganese, zinc and phosphorus found in SP were reported to be 25.75, 542.13, 486.25, 0.87, 17.99, 2.93, 1.80 and 209.90 mg/100 g of DM, respectively (Khoshbakht Fahim et al. 2012). A further study on saffron residues revealed that these residues provide appropriate nutritional value for livestock, especially in the late stages of vegetative growth, with effective degradability, CP, NDF, water‐soluble carbohydrates and calcium percentages reported as 69.6%, 13.9%, 32.5%, 27.2% and 1.3%, respectively (Kardan Moghadam et al. 2014). A moisture content of 91.33% has been reported for SP (Alipour et al. 2016). The concentrations of ash, CP, fat and total carbohydrates in SP were reported as follows: 7.08% ash, 6.35% CP, 3% fat and 71.16% total carbohydrates (Jadouali et al. 2018). One of the key strategies for reducing wastewater production in silos is the use of moisture‐absorbing materials, which retain nutrients within the silo by absorbing the produced leachate (Seidali Dolat‐Abad et al. 2016). In a study, wheat bran (WB) was used as a moisture‐absorbing substance for silage of high moisture citrus pulps (Kordi and Naserian 2012). Saffron has diverse applications across the food, beverage, pharmaceutical and cosmetic industries. Like other phytochemicals, saffron and its BPs are considered important sources of bioactive natural compounds. The health benefits of saffron are well‐established, particularly its antioxidant and anti‐inflammatory properties, which help reduce pro‐inflammatory cytokines and are acknowledged in internal medicine (Marrone et al. 2024). Specifically, saffron's health benefits are linked to its ability to combat degenerative maculopathy, depression, anxiety, neurodegenerative disorders, metabolic syndrome, cancer and chronic kidney disease, while also enhancing glucose metabolism. A review emphasizes key studies that showcase saffron's potential as a valuable ally in the prevention and treatment of various conditions. Additionally, it advocates for the use of saffron BPs within a bio‐circular economy framework, which aims to minimize waste, optimize resource utilization and promote environmental and economic sustainability (Marrone et al. 2024). In a study, the researchers assessed the chemical composition, silage characteristics, digestibility and in vitro gas production parameters of saffron waste (SW), specifically petals and stamens, both prior to and following the ensiling process (Kazemi et al. 2020). The experimental design comprised seven treatments, each with four replicates: (1) SW before ensiling (SWBE); (2) SW after ensiling (SWAE); (3) a mixture of 96.88% SWAE and 3.12% WB based on fresh weight; (4) a mixture of 93.75% SWAE and 6.25% WB (fresh weight); (5) a mixture of 87.5% SWAE and 12.5% WB (fresh weight); (6) a mixture of 75% SWAE and 25% WB (fresh weight) and (7) a mixture of 50% SWAE and 50% WB (fresh weight). The second treatment exhibited poor quality and an undesirable odour as a result of significant mould growth and adhesion. However, the addition of WB, especially in larger amounts (as seen in treatments 4, 5 and 6), significantly improved the quality of the silage (Kazemi et al. 2020). The chemical composition of the treatments varied, with DM ranging from 10.40% to 54.37% (fresh weight), NDF from 12.83% to 27.35%, ADF from 7.23% to 11.45%, CP from 14.88% to 15.67%, EE from 5.43% to 5.77% and ash content from 5.89% to 11.12% (dry weight) (Kazemi et al. 2020). Notably, the highest concentrations of NDF and ADF (27.35% and 11.45%, respectively) were recorded in treatment 7 (Kazemi et al. 2020). Furthermore, treatment 7 also exhibited the lowest pH values, along with the highest levels of lactic and acetic acids, gas production after 12 and 24 h of incubation and a consistent gas production rate. True DM digestibility varied between treatments, with values ranging from 76.30% for treatment 2 to 79.95% for treatment 1 (Kazemi et al. 2020). Overall, the study concluded that SW possesses good nutritional value prior to ensiling, whereas ensiling without additives diminishes its quality. Additionally, the authors indicated that ensiling SW with WB as a moisture‐absorbing agent did not adversely affect certain nutritional parameters (Kazemi et al. 2020). They further observed that treatment 7 demonstrated the most favourable conditions in terms of appearance, odour, absence of mould and fermentation characteristics within the silo environment (Kazemi et al. 2020). Recent pharmacological research suggests that saffron extract exhibits a range of beneficial effects, including antitumor, anticonvulsant, antidepressant, anti‐inflammatory, anti‐lipid, anti‐instigative, antihyperlipidemic and antioxidant properties (Asdaq and Inamdar 2009; Melnyk et al. 2010; Goli et al. 2012). It has been proposed that certain BPs derived from Moroccan saffron could be used as feed supplements for ruminants throughout both the wet and dry seasons (Jadouali et al. 2021). When used as a feed additive, saffron has the potential to serve as a natural colourant and enhance antioxidant activity. Studies suggest that it may improve the oxidative stability of poultry meat and eggs (Botsoglou et al. 2010). Additionally, evidence has been reported regarding the chemopreventive and protective effects of saffron extract against oxidative stress induced by genotoxic agents in animal models (Asdaq and Inamdar 2009). Further investigations have highlighted the pharmacological benefits of SW in treating certain gastrointestinal diseases, which are attributed to its content of phenolic compounds and phytosterols (Ashktorab et al. 2019; Rezaee Khorasany and Hosseinzadeh 2016).

**TABLE 4:** Investigation on the use of saffron by‐products in animal diets.

Pomegranate by‐product	Animal	Offered amount	Finding	Reference
Petal	In vitro using lamb ruminal fluid	1%, 2% and 3% of DM	· Positive effects of SP on rumen fermentation · Increased cellulolytic bacteria and fibrolytic enzyme activity in rumen fluid · Decreased protozoa population with SP inclusion · Significant improvement in antioxidant capacity of fermented rumen fluid · Non‐significant decrease in proteolytic bacteria and protease levels · Enhanced digestibility of the diet · Reduction in ammonia and methane production of rumen fluid · Optimal effects were observed with the inclusion of 2% and 3% of SP · SP has the potential to be used as a natural phytobiotic additive at levels up to 3%	Akbari Shooshood et al. (2024)
Stigma extract	Wistar Albino rats	80 mg/kg BW	· Beneficial properties of saffron include its anti‐apoptotic, anti‐inflammatory and antioxidant effects · Numerous applications as a flavouring and herbal remedy · Protective efficacy of saffron against AFB_1_ toxicity in Wistar albino rats · BW changes showed significant decreases in all treatment groups compared to the control group · Increase in basophils, platelets, monocytes and lymphocytes; and a decrease in neutrophils and eosinophils · Increase in serum levels of uric acid, creatinine, AST, alkaline phosphatase, nitric oxide and MDA; reduced testosterone levels in the AFB1 group · The AFB1 group displayed alterations in testes, liver and kidney tissues · Saffron administration restored the oxidative stress biomarkers and normal tissue structure, similar to the control group	Ashi et al. (2024)
Stigma extract	Rat	200 mg/kg BW	· Acrylamide identified as a dietary pollutant linked to liver and kidney damage · Saffron and its constituents suggested to have preventive properties against digestive and urinary tract disorders · Significant increases in creatinine, urea, protein fractions and serum minerals observed in rats receiving both acrylamide and saffron extract with Vit C · Delayed administration of saffron extract and Vit C after acrylamide exposure still resulted in elevated liver function tests and minerals · Saffron extract and Vit C demonstrated potential to reverse alterations caused by acrylamide	Moustafa Omar (2023)
Ethanolic SP	Baluchi male lamb	25 mg/kg BW or 500 mg oral dose/kg BW	· No significant effects on growth performance or many blood metabolites · Plasma cholesterol levels in ISPE and Vit E groups lower than OSPE and control · ISPE group exhibited lower plasma triglycerides compared to OSPE and Vit E · Highest plasma GPx activity found in OSPE group · ISPE and Vit E groups demonstrated higher SOD activity than control · ISPE group had lower plasma MDA levels compared to OSPE · Treatment effects on GPx and SOD activities in kidney and heart · Addition of ethanolic SPE improved antioxidant status and reduced lipid oxidation in lambs	Alipour et al. (2019)
Green zinc oxide (gZnO) nanoparticles produced from SP	Ram	7.5, 10 and 12.5 µg/mL of semen	· 12.5 µg/mL gZnO nanoparticles had destructive effect on sperm quality · Lower gZnO levels (7.5 and 10 µg/mL) increased sperm motility · Significant improvement in membrane integrity at 7.5 µg/mL gZnO · Antibiotics combined with gZnO reduced microbial load · Strong positive correlation between zinc and sperm motility · Zinc's antioxidant power reduces reactive oxygen species and lipid peroxidation · Zinc nanoparticles stabilized the membrane lipids and enhanced mitochondrial activity · Higher concentrations of gZnO (>10 µg/mL) have toxic effects on sperm · The utilization of gZnO nanoparticles decreases both antibiotic usage and cytotoxic effects · The combined effects of gZnO and antibiotics strengthened antibiotic efficacy and diminished resistance · The optimal concentrations of gZnO (7.5 and 10 µg/mL) resulted in the most significant enhancement of frozen–thawed sperm quality · Combined use of gZnO and antibiotics increased effectiveness and reduced toxicity	Khoshvaght et al. (2024)
Petal extract	Laying hens	40, 60 and 80 ppm	· Significant decrease in yolk cholesterol with saffron extract inclusion · Feed intake, feed conversion ratio and egg weight remained unaffected · Increased egg production percentage in saffron‐fed hens compared to control · Lower serum cholesterol in saffron‐fed hens · Decreased blood glucose and triglyceride levels noted with 80 ppm saffron extract · Faecal minerals excretion unchanged across treatments · Significantly lower NH_3_ gas emissions in faeces from hens receiving 60 and 80 ppm saffron extract · An extract of saffron at a concentration of 80 ppm was effective in lowering cholesterol levels in egg yolk and serum, as well as in reducing faecal ammonia emissions	Vakili and Mokhtarpour (2023)

Abbreviations: AFB_1_, aflatoxin B_1_; AST, aspartate transferase; BW, body weight; DM, dry matter; GPx, glutathione peroxidase; gZnO, green zinc oxide nanoparticles; ISPE, injected saffron petal extract; MDA, malondialdehyde; OSPE, oral saffron petal extract; SOD, superoxide dismutase; SP, saffron petal; SPE, saffron petal extract; Vit, vitamin.

Research on SWs as a dietary supplement for small ruminants is limited. Therefore, two experiments were conducted to evaluate the nutritional potential of SW (Kazemi 2024). The first trial included a proximate analysis of SW from various regions of northeast Iran, revealing variability in its chemical and mineral composition. The second trial evaluated the incorporation of a 1:1 mixture of SW at two levels (30 and 60 g/day) in the diets of Afshari male lambs. Although DMI and nutrient digestibility remained unaffected, certain health indicators, including MDA, TAC and cholesterol levels, showed significant changes in lambs fed 60 g SW/day. This finding highlights the need for further long‐term investigations to evaluate the in vivo effects of SW (Kazemi 2024). A study examined the impact of SP supplementation on the lactation performance, nutrient digestibility and antioxidant status of dairy goats (Ebrahimi et al. 2024). Eighteen multiparous Saanen goats were assigned to three groups receiving diets with 0%, 1.5% and 3% SP. Results indicated that although DMI and nutrient digestibility remained constant, the 3% SP group experienced a significant increase in milk production and protein content. Additionally, plasma glucose and cholesterol levels decreased, whereas TAC improved in both plasma and milk. The study concluded that up to 3% SP supplementation positively affects milk yield and antioxidant status without harming nutrient digestibility (Ebrahimi et al. 2024). A study investigated the effects of different extraction methods on the antioxidant activity of CC and their performance in vegetable oils (Najafi et al. 2022). Saffron stigmas were extracted using various solvents and methods, revealing that the methanol/water (50:50) extract, obtained through a combination of ultrasonic‐assisted and microwave‐assisted extraction, exhibited the highest TPCs (31.56 mg/g GAE) and antioxidant activity (83.24% inhibition). The freeze‐dried saffron extract, containing key bioactive compounds, significantly enhanced the oxidative stability of canola, sunflower and corn oils when added at 1000 ppm (Najafi et al. 2022). Furthermore, the extraction efficiency of bioactive compounds from saffron anthers was investigated using various solvents, including ethanol, methanol and distilled water (Mahood et al. 2023). Ethanol was found to be the most effective solvent, yielding the highest TPCs, total flavonoid content (TFC) and antioxidant activity, with flavonoids showing superior antioxidant properties compared to ascorbic acid. Notably, key compounds identified included gallic acid, syringic acid, vanillic acid and various flavonoids. This research highlights that saffron anthers, typically regarded as agricultural waste, are a valuable source of bioactive compounds, offering potential economic benefits through the valorization of saffron BPs in the Mashhad region of Iran (Mahood et al. 2023). In another research study, the nutritive value of saffron residues was evaluated by determining their chemical composition, in situ degradability and in vitro gas production using two permanently fistulated Holstein heifers (Kardan Moghaddam et al. 2015). The analysis indicated that the composition of saffron forage consists of 96.8% OM, 6.7% CP, along with noteworthy amounts of both fibre and NFC. However, essential minerals such as sodium, magnesium, zinc and iron were found to be insufficient for ruminant requirements. The study indicated that the rapidly and slowly degradable fractions were 32% and 39.2%, respectively, whereas the OM digestibility was 53.9%. The research concluded that saffron forage could serve as an economical feedstuff alternative within animal diets (Kardan Moghaddam et al. 2015).

## 6 Raisin BPs (RBs)

**FIGURE 5:**
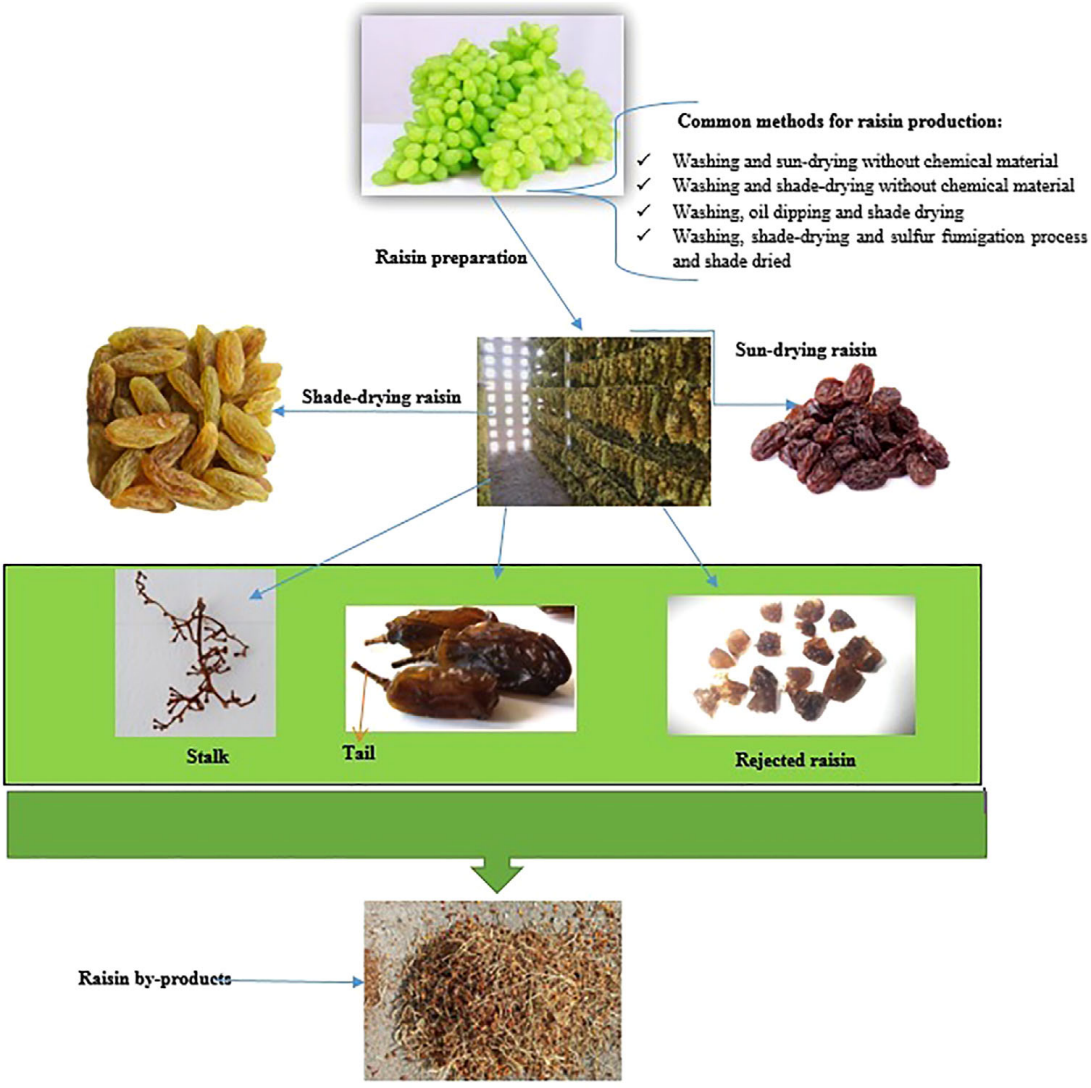
A schematic of raisin preparation and its by‐products.

Raisins are the ripe, dried fruit of grapes, marketed under various names depending on the type of grape, the drying method and conditions and permissible additives. Like other dried fruits, raisins are available throughout the year. To produce raisins, grapes with white flesh and green skins are used; after drying, their colour changes and darkens, ultimately turning into a sweet snack with small seeds. Raisins are rich in iron, potassium, calcium and B vitamins, and they possess antioxidant properties that help prevent cellular damage (Schuster et al. 2017; Thiruchelvi et al. 2020). They contain a significant amount of fibre, antioxidants and energy. The fibre in raisins can help prevent colon cancer and abnormal cell growth, as well as aid in the control of blood sugar levels, making them beneficial for treating stomach issues and constipation. Eating raisins helps combat fatigue and lethargy while also strengthening the body (Schuster et al. 2017; Thiruchelvi et al. 2020). The bioflavonoids found in raisins contribute to detoxification and purification of the blood and the body, supporting liver health and the treatment of liver diseases. Nutritionists believe that eating a few raisins daily can enhance memory and may protect individuals from Alzheimer's disease. Additionally, the high fibre content in raisins aids in reducing cholesterol levels and improving intestinal function. Potassium, which is abundantly present in raisins, helps to lower blood pressure and prevent fluid retention in the body (Schuster et al. 2017; Thiruchelvi et al. 2020). The high calcium content in raisins supports bone health and helps prevent osteoporosis. Moreover, raisins contain selenium, which contributes to skin clarity and nourishment. The Iranian grape, scientifically classified under the family Vitaceae and the species *V. vinifera* (commonly referred to as European grape), has seen an increase in the establishment of agricultural processing factories in recent years. A schematic of raisin preparation and its BPs are shown in Figure 5. This figure outlines several different methods for producing raisins. Generally, during the production of raisins, the grapes undergo an initial washing and the removal of unhealthy or damaged fruits before being dried without the addition of chemicals, either in the shade or under sunlight. Grapes dried in the shade are referred to as ‘sun‐dried raisins’, whereas those dried in direct sunlight are called ‘shade‐dried raisins’. In some instances, after thoroughly washing the grape clusters, they are briefly immersed in a special oil and then dried in designated areas using metal wire rods, away from sunlight and in the shade. Another method of raisin production involves washing the grape clusters and discarding unsuitable fruits, then drying them in the shade followed by sulphur fumigation. This last process typically results in golden‐coloured raisins, which are commonly known as ‘smoked raisins’ in Kashmar, a city in the Razavi Khorasan province. Although there may be various other methods worldwide for producing raisins, Figure 5 highlights the most important ones. It is noteworthy that during the preparation of raisins for human consumption, waste products such as rejected raisins (those that are broken and not suitable for human consumption), tails and stalks are generated during the processing of raisins and at cleaning factories, which can have nutritional potential for livestock. These facilities often produce a significant amount of agricultural waste, including BPs from raisin production. Notably, the processing of raisins generates approximately 900 t of pomace and tails, alongside 135 t of sugared raisin and 463 t of other waste annually. On the other hand, RBs comprise the skins and stems of grapes, as well as unripe berries along with their stems, leaves and rachises generated during the production of raisins (Ahmadi Kohanali et al. 2022). Unfortunately, these BPs are often incinerated despite their potential utility in the food industry and are typically disposed of in the surrounding environment without proper treatment (Yari et al. 2016). The use of RBs, especially in animal feed, presents a promising opportunity to reduce environmental pollution while addressing certain nutritional needs in livestock. However, it is crucial to note that these BPs might also contain anti‐nutritional factors like tannins and lignin (Alipour and Rouzbehan 2007; Besharati and Taghizadeh 2009; Yari et al. 2016). High levels of tannin in the diet can negatively impact livestock health by binding to proteins, minerals and carbohydrates, which may impair microbial activity and disrupt digestion in both the rumen and intestines (McSweeney et al. 2001; Besharati and Taghizadeh 2009). However, tannins can also affect protein metabolism in ruminants by reducing the ruminal degradation of dietary protein. This may lead to more protein reaching the intestines, potentially increasing the concentration of VFAs and providing beneficial effects (Besharati and Taghizadeh 2009; McSweeney et al. 2001; Yari et al. 2016). Adding tannin‐rich materials into animal diets has been proposed as a strategy to reduce protein degradability in the rumen (McSweeney et al. 2001). Additionally, it is important to note that some RBs contain high levels of insoluble carbohydrates, mainly made up of simple sugars (Yari et al. 2016). A study investigated the use of RBs as a substitute for barley grain in diets that included cereal straw for growing ram lambs (Ahmadi et al. 2022). Three iso‐nitrogenous and iso‐energetic diets with RBs replacing 9% and 18% of barley were tested on 12 lambs using a 3 × 3 Latin square design. The nutritional composition of RBs showed 78.4% DM, 3.7% CP and significant fibre content. The results indicated that substituting ground barley with RBs did not affect final body weight, ADG, feed intake or nutrient digestibility. However, blood urea levels decreased, whereas blood total protein and triglycerides increased at the 9% substitution level.

The findings suggest that up to 18% of barley can be replaced with RBs in lamb diets without negatively impacting growth performance or health metrics (Ahmadi et al. 2022). Furthermore, another study examined the sustainable potential of biomass produced during the grape drying process, analysing 11 different RBs for their organic content and energy properties (Okasha et al. 2023). Key findings included significant variations in carbon, nitrogen, hydrogen and oxygen levels among RBs. BPs no. 10 and no. 11 exhibited the highest calorific values of 22.73 ± 0.08 and 22.80 ± 0.07 MJ/kg, whereas BPs no. 5%–9% had minimal lignin content. The maximum biogas production was observed in BP groups C (11.50 NL/L) and B (11.20 NL/L). The research concludes that biomass from various raisin production stages shows promise as a solid fuel and energy source, particularly suitable for pyrolysis (Okasha et al. 2023). Some researchers investigated the nutritive value of several RBs for ruminants in semi‐arid conditions, employing the National Research Council feeding system and in vitro gas production methods (Yari et al. 2015b). The study identified three types of RBs: outer layers of flesh and skin (RBs1), rejected raisins (RBs2) and peduncles with branches (RBs3) (Yari et al. 2015b). Results showed that RBs1 and RBs2 had significantly higher predicted ME and net energy for lactation compared to RBs3. Additionally, RBs2 exhibited superior in vitro gas production, energy estimates and OM digestibility. The findings suggest that RBs can serve as alternative feeds for ruminants during dry periods (Yari et al. 2015b). Similarly, in another study, the nutritional value of several RBs for ruminants was evaluated, with the aim of addressing feed shortages during dry periods (Yari et al. 2015a). The RBs assessed include different parts of the grapevine (flesh and skin; rejected raisins and branches). Results indicate that RBs1 had the lowest NDF and lignin content, whereas RBs3 had the highest. RBs1 and RBs3 also exhibited higher total tannin concentrations compared to RBs2. In situ ruminal degradation analysis showed that RBs1 had better degradability and lower undegradable fractions than RBs3, with RBs2 being intermediate. The findings suggest that RBs can serve as alternative ruminant feed during dry periods, although their tannin and lignin levels must be carefully considered. Additionally, the nutritional value of RBs as feed for ruminants was investigated, particularly in semi‐arid climates like Malayer in Hamedan province (Yari et al. 2016). The objective is to compare this BP with late‐flowering alfalfa hay and evaluate its impact on the in vitro fermentation of alfalfa. RBs revealed significantly higher total phenol, tannin and OM content compared to alfalfa hay, while demonstrating lower values for DM, CP and NDF (Yari et al. 2016). The in situ results indicated that RBs had higher degradability for DM and OM but lower degradability for NDF and CP. The study also found that increasing levels of RBs improved the rate of gas production and nutrient supply, largely due to its higher NFC content and the presence of tannins, which appeared to influence fermentation dynamics (Yari et al. 2016). The researchers concluded that the RBs can be considered a viable feed option for ruminants, enhancing the in vitro gas production kinetics of alfalfa hay and overall nutrient efficiency (Yari et al. 2016). The effects of varying inclusion levels of RB in the diets of growing lambs were studied, involving 24 male lambs aged 6 months (Saremi et al. 2014). Four inclusion levels were tested: R0 (0 g), R1 (100 g), R2 (200 g) and R3 (300 g) RB/kg of DM. The study assessed animal performance, ruminal parameters and protozoa populations. Results indicated that R2 and R3 diets led to the highest final body weights. However, R3 exhibited the lowest DMI and feed conversion rate. Although total protozoa numbers increased with RB inclusion, *Epidinium* spp. disappeared in the R3 diet. Dietary inclusion of RBs above 200 g/kg DM significantly decreased digestibility of CP and NDF. The study concludes that RBs can be included in growing lamb diets up to 200 g/kg DM without adversely affecting production performance (Saremi et al. 2014). In another study, an experiment was conducted to evaluate the impact of PEG (PEG‐6000) and urea on in vitro DM and OM digestibility (IVDMD and IVOMD) and gas production in vitro, using raisin stalks (Angaji et al. 2011). The raisin stalks contained 8.6% CP and significant levels of total extractable phenol and tannin. Treatments included control (no supplementation), 3% urea, 5% urea and a combination of 3% urea with 5% PEG. Results showed that PEG significantly enhanced IVOMD and IVDMD and increased gas production during incubation compared to other treatments. The study concluded that PEG treatment can mitigate the negative effects of tannins on digestibility and gas production in raisin stalks (Angaji et al. 2011). Because in raisin production factories, raisin processing waste is viewed as a low‐value BP, less attention may be paid to maintaining its quality, and due to storage for a relatively long time, anti‐nutritional substances such as mould may be observed in it. In general, it should be noted that raisin waste may contain anti‐nutritional substances such as tannins and lignin. Feeding high levels of tannins can negatively affect animal performance due to the binding of tannins with proteins. However, the presence of a certain amount of tannin in the diet can have beneficial effects on protein metabolism and animal performance by reducing protein degradation in the rumen and increasing the amount of protein that passes to the intestine.

## 7 Olive BPs

**FIGURE 6:**
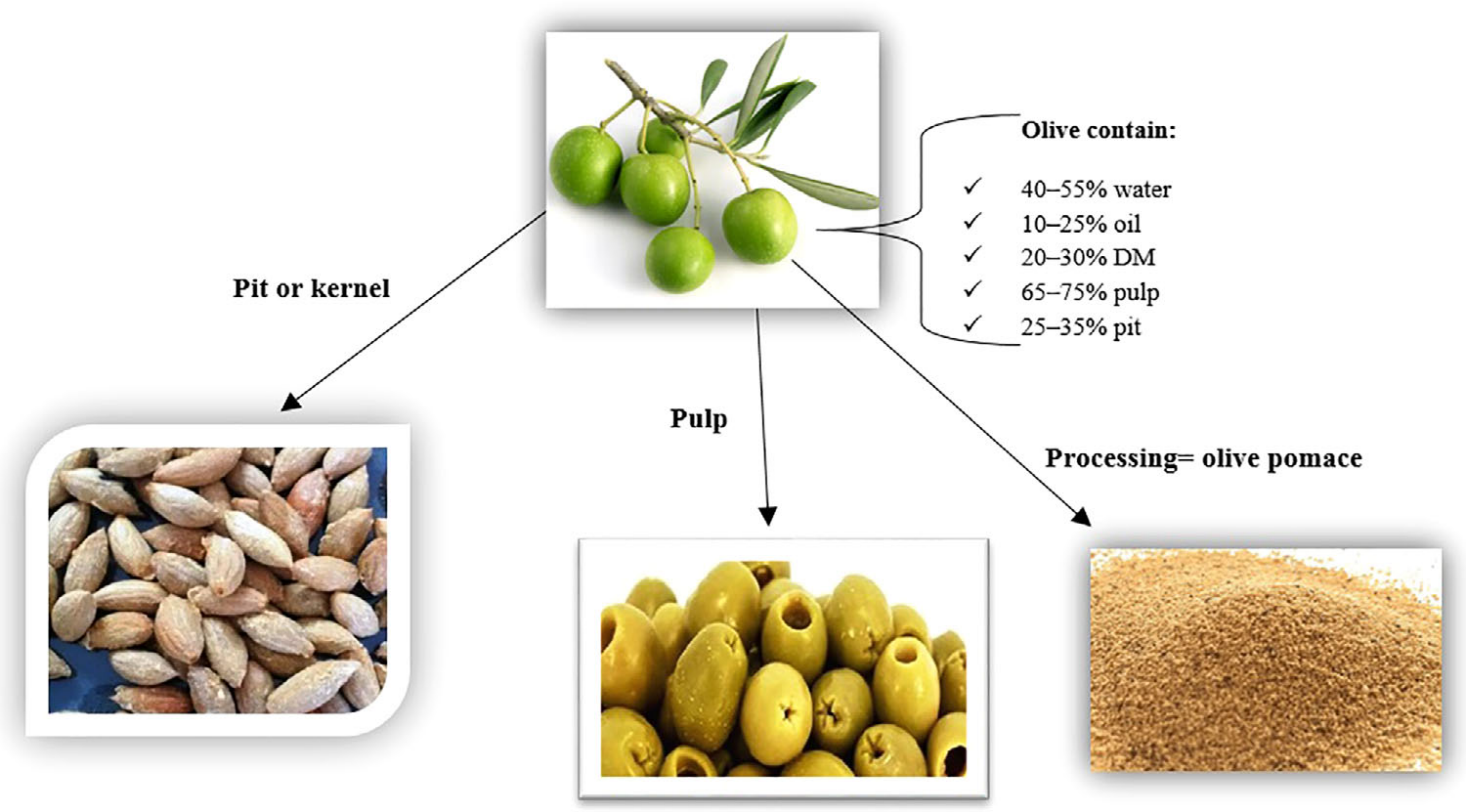
A schematic of olive and its by‐products.

Olive pomace (OP), also known as oil cake, is a BP generated during the oil extraction process. It comprises the skins, pulp and pits of olives, along with a residual quantity of fat, and can represent up to 80% of the total processed fruit (Monteiro et al. 2024). This BP is rich in bioactive compounds, making comprehensive characterization essential to explore its potential in functional foods, nutraceuticals and pharmaceuticals for both humans and animals (Monteiro et al. 2024). A schematic of the olive and its BPs is illustrated in Figure 6. This figure illustrates the BPs generated from olives, which mainly include pits or kernels and pulp that are no longer suitable for human consumption. These BPs can be easily converted into animal feed through various processing methods. In vitro studies indicate that biophenols from OP exhibit antioxidant, anti‐inflammatory and antiproliferative properties, which may help mitigate cardiovascular disorders and improve animal immunity and meat quality. Although human clinical trials are limited and show minimal biological changes, some biomarkers suggest potential cardioprotective effects (Monteiro et al. 2024).

In industrialized societies, waste from olive production poses a significant challenge, as international statistics indicate that approximately 5%–10% of agricultural production is discarded as waste. This waste has the potential to serve as a food source for a significant population. OP enhances the flavour of meat in livestock and poultry and provides advantages over traditional feeds in animal husbandry. However, it also presents certain drawbacks. Notably, the high moisture and oil content in OP can lead to mould formation when exposed to air, rendering it unfit for consumption as feed. To prevent mould growth, OP is pressed to remove its moisture and oil and subsequently dried at high temperatures to produce olive meal for animal feed. This process increases the shelf life of olive meal, resulting in a healthier feed option for livestock and poultry. In Iran, approximately 50,000 t of OP are produced annually in oil extraction facilities, with about 50% of this material composed of water. From this total, around 20,000 t of usable waste are obtained, which can be utilized as animal feed. OP contains adequate amounts of oil and can be considered an energy‐generating food source. Its use in feeding livestock, poultry and aquatic animals leads to the reduction of environmental pollution and ration cost, but the amount of consumption of each food item in the ration is important. It is another important thing that should be paid enough attention to, and it is possible that the consumption of an edible substance in a certain amount in the diet has beneficial effects on the growth and breeding of fish, but at higher levels, it is not only not fruitful but also causes a decrease in growth and adverse effects. One of the most important limitations of using OP is the variability of its chemical composition and the presence of large amounts of anti‐nutritional compounds such as tannins, which in combination with dietary protein and carbohydrates reduce the activity of digestive enzymes. OP is a valuable source of oil and can serve as an energy‐rich feed ingredient. Its incorporation into the diets of livestock, poultry and aquatic animals can help reduce environmental pollution and lower feed costs. However, the quantity of each feed component in the diet is crucial. It is essential to recognize that although moderate consumption of OP can positively influence the growth and reproduction of fish, excessive amounts may lead to detrimental effects, including reduced growth rates. A significant challenge in utilizing OP is the variability in its chemical composition, as well as the presence of substantial levels of anti‐nutritional compounds, such as tannins. These compounds can interact with dietary proteins and carbohydrates, inhibiting the activity of digestive enzymes. In the Mediterranean region, olive oil production generates bio‐waste such as olive oil pomace and olive tree leaves, which can be utilized in animal diets (Scicutella et al. 2024). Investigations on the use of olive BPs in animal diets are presented in Table 5. Two in vitro trials examined the effects of these co‐products on rumen fermentation and microbiome ecology, using isoproteic and isoenergetic diets (Scicutella et al. 2024). Results indicated that both olive oil pomace and olive tree leaves increased the levels of beneficial fatty acids (C18:1 c9 and C18:3 c9c12c15) while selectively altering microbial communities, reducing the abundance of certain genera like *Butyrivibrio* and *Pseudobutyrivibrio* and increasing others such as *Christensenellaceae_R‐7_group* and *Manheimia*. This study demonstrates the potential of these BPs to effectively modulate rumen microbial communities (Scicutella et al. 2024). Furthermore, in another study, the effects of adding olive cake (OC) into the diets of indigenous Bísaro pigs were evaluated, focusing on the quality of processed meat products (Leite et al. 2024). Loins and ‘cachaços’ were produced using a standardized process, revealing significant differences in physicochemical properties such as water activity, moisture, total fat, protein and haem pigments (Leite et al. 2024). The diet also significantly influenced NaCl content. However, the diet did not affect the proportions of saturated, monounsaturated or PUFAs, although a significant increase in *n*‐3 fatty acids was noted. The diet with 25% centrifuged OC yielded the highest levels of *n*‐3 fatty acids, resulting in a lower PUFAs *n*‐6/*n*‐3 ratio compared to the control (Leite et al. 2024). OC constitutes approximately 35% of the weight of olives processed and serves as a solid BP of olive oil extraction (Difonzo et al. 2022; Amato et al. 2024). Given its significant environmental impact, there has been a surge of research in recent years focusing on sustainable applications for this BP. This includes energy production, the development of new materials, pharmaceutical uses, food products and animal feeds (Espeso et al. 2021; Amato et al. 2024). In particular, the addition of OC into animal feed presents a noteworthy alternative to conventional feed for both monogastric animals (Leite et al. 2024) and ruminants (Abbeddou et al. 2014; Hafez et al. 2024), owing to its high content of water‐soluble polyphenols (such as hydroxytyrosol and tyrosol) and UFAs, particularly oleic acid (C18:1 *cis*‐9). Among the various methods for olive oil extraction, the two‐phase milling process is noted for its ability to yield a greater quantity of polyphenols (Scicutella et al. 2023). Numerous studies have identified feeding strategies that can enhance the fatty acid composition of milk, which is linked to beneficial metabolic effects in humans (Amato et al. 2024) and serves as an important indicator of the metabolic and energy status of cows (Giannuzzi et al. 2022; Amato et al. 2024). Reports indicate that cows fed diets supplemented with OC exhibit increased levels of MUFAs in their milk, a reduction of SFAs and lower thrombogenic and atherogenic indices (Castellani et al. 2017). These findings are supported by additional studies (Chiofalo et al. 2020; Neofytou et al. 2023), which have demonstrated that the inclusion of OC, whether ensiled or dried, in ruminant diets can serve as an effective nutritional strategy to enhance the composition of dairy products while simultaneously reducing animal feeding costs. Researchers investigated the chemical composition, fatty acid profile and polyphenol content of Provola cheese produced from the milk of cows whose diets were supplemented with OC (Attard, Bionda, et al. 2024). The study, conducted over several months, demonstrated that both the diet and seasonal variations significantly influenced the composition of the cheese. Notably, the cheese produced in spring from cows on the supplemented diet exhibited the most favourable health attributes, characterized by lower atherogenic and thrombogenic indices alongside a higher total polyphenol content. Specifically, cheese from the treated group had a 32.9% increase in polyphenol levels compared to the control group, enhancing its nutritional functionality. Overall, the inclusion of dried and stoned OC in dairy cow diets not only fosters sustainable production practices but also enhances the nutritional quality of the resulting cheese (Attard, Bionda, et al. 2024). Moreover, another study evaluated the effects of substituting 20% of the TMR with milled olive cake (MOC), with or without the addition of PEG or fibrolytic enzymes, on the productive performance of Barki sheep (Bakr et al. 2024). In the first experiment, five rations were compared, revealing that PEG had no significant impact, making the fourth group (20% MOC) the most effective (Bakr et al. 2024). The second experiment involved 18 Barki lambs divided into three groups, where the ration containing 20% MOC resulted in decreased DM and OM digestibility, with significant differences in CP digestibility (Bakr et al. 2024). Rumen pH and ammonia levels remained unchanged across groups. However, significant differences were noted in total gain, ADG and feed efficiency, with a slight increase in DMI in the group fed the ration with fibrolytic enzymes (R3) (Bakr et al. 2024). It is reported that OP is rich in polyphenols, known for their health benefits, including anti‐inflammatory and antibacterial properties (Cavallucci et al. 2024). The anti‐inflammatory effects of in vitro digested OP were examined as a potential functional ingredient in horse feed, suggesting that OP could serve as a valuable source of nutraceuticals while also mitigating the environmental impact of olive oil BPs (Cavallucci et al. 2024). Although horses can utilize dietary fat effectively, the fat content in commercial feeds is often restricted (Cavallucci et al. 2024). The impact of a solid‐state‐fermented mixture of olive mill stone waste (OMSW) and *Lathyrus clymenum* husks (LP) on the antioxidant blood parameters of weaned piglets was investigated in another study (Eliopoulos et al. 2024). Two hundred piglets were divided into two groups, receiving either a control diet or a diet with 50 g of OMSW‐LP per kg for 40 days. Blood examinations indicated that the OMSW‐LP diet significantly reduced thiobarbituric acid‐reactive species and protein carbonyls, while enhancing free radical scavenging activity, reduced glutathione levels and CAT activity. These findings suggest that the dietary inclusion of solid‐state‐fermented OMSW‐LP can improve antioxidant profiles, which is essential for the health and growth of piglets post‐weaning (Eliopoulos et al. 2024). The mineral composition of dairy products is influenced by various factors, including the genetic traits of the dairy cows, their lactation stage and their diet. In this context, a study was conducted to examine the mineral content in Provola cheeses produced from dairy cows that were fed two different integrated diets: one supplemented with Biotrak and another serving as a control group without any additives (Potortì et al. 2024). The findings indicated that the Biotrak cheeses had significantly higher levels of essential minerals, particularly selenium, which ranged from 0.112 to 0.281 mg/kg, approximately double that of the control cheeses. Only cadmium was detected among the toxic elements, with levels averaging below 0.11 mg/kg. Thus, the addition of OC in animal feed effectively enhances the mineral profile of the resulting cheese (Potortì et al. 2024). The environmental impact of substituting apple and OP for maize grain in the diets of fattening pigs was investigated (Rebolledo‐Leiva et al. 2024). Using life cycle assessment (LCA), the study evaluates four diet alternatives, highlighting a diet with 33% maize and 43% subproducts as the most environmentally effective. It emphasizes the importance of allocation methods for subproduct loads, such as mass, economic and zero‐burdens allocation, to validate strategy assumptions. The consequential LCA suggests the strategy may have both positive and negative environmental impacts, depending on substitutes for maize stover and displaced bioproducts, like those for bioenergy (Rebolledo‐Leiva et al. 2024). In another study, the transformation of OMSW and walnut shell (WS) into protein‐rich animal feed was explored using solid‐state fermentation with the *fungus Pleurotus ostreatus* (Arapoglou et al. 2024). A substrate composed of 80% WS and 20% OMSW resulted in the most significant protein enhancement, achieving 7.57% of its total mass, which represents a 69.35% increase (Arapoglou et al. 2024). Additionally, this combination led to a 26.13% reduction in lignin content. Furthermore, the procedure increased the β‐glucan content threefold, reaching 6.94% of the substrate's mass (Arapoglou et al. 2024). These findings suggest the potential of OMSW and WS mixtures as effective substrates for developing novel animal feed, aiding in addressing protein shortages and reducing the environmental impact of agro‐industrial processes in‐line with circular economy principles (Arapoglou et al. 2024). In a separate study, the main phenolic compounds identified in spray‐dried olive oil mill wastewater were analysed. These compounds included oleuropein derivatives (54.67%), tyrosol (17.03%), hydroxytyrosol (12.35%), verbascoside (5.83%) and hydroxytyryloleate (4.70%) (Cifuni et al. 2023). Additionally, the wastewater contains other phenolic compounds such as caffeic acid, vanillic acid, *p*‐coumaric acid, apigenin, luteolin, diosmetin, rutin and oleuropein (Cifuni et al. 2023). The total concentration of the identified phenolic compounds in the spray‐dried olive oil mill wastewater was found to be 11,991 mg/kg (Cifuni et al. 2023). In a study, the impact of a novel silage, composed of olive mill wastewater, GP and deproteinized feta cheese whey, in broiler chicken diets was assessed (Bonos et al. 2022). A total of 216 male Ross‐308 chicks were allocated to three groups receiving diets with 0%, 5% or 10% silage. Results showed that a 10% silage inclusion significantly increased final body weight and feed intake, and it positively modified jejunum and cecum microflora, as well as meat microflora (Bonos et al. 2022). Additionally, thigh meat's oxidative stability improved, and significant differences were observed in fatty acid profiles between supplemented treatments and controls. They demonstrated that the novel silage could enhance broiler performance and meat quality (Bonos et al. 2022). Due to rising animal feed costs, there is an ongoing need for alternative funding sources to replace high‐cost raw materials in animal feed. In a study, researchers explored the effects of alkaline hydrogen peroxide (AHP) pretreatment on improving the in vitro rumen digestibility of exhausted OP (EOP), which is a widely available agricultural BP (Masmoudi et al. 2024). Under optimized conditions (1.6% H_2_O_2_, 5% NaOH), the phenolic content in treated OP (TOP) significantly decreased, and approximately 25% of lignin was removed. TOP exhibited improved nutritional parameters, showing higher yields of CP and fibres compared to untreated EOP. Enzymatic hydrolysis demonstrated a 48% reducing sugar yield for TOP, whereas IVOMD and ME were also significantly higher in TOP than EOP. Overall, AHP pretreatment effectively detoxifies EOP and enhances its suitability as animal feed (Masmoudi et al. 2024). Additionally, a study focused on the incorporation of OP into ruminant feed, emphasizing its potential to address environmental issues related to OP disposal, decrease food‐feed competition and improve food security (Mnisi et al. 2024). Despite its benefits, OP's high fibre and low CP content limit its nutritional value. The researchers evaluated the impact of oyster mushrooms (OYM) on the nutritive value of OP by assessing its chemical composition and in vitro ruminal fermentation parameters after cultivating OYM on varying percentages of OP. Results indicated that OYM significantly improved the nutritional profile of OP by increasing DM, OM and CP while decreasing fibre content. This bioconversion approach not only enhances feed quality but also addresses waste disposal concerns and offers economic benefits to the olive oil industry (Mnisi et al. 2024). In another study, six types of OC from the Trás‐os‐Montes and Alto Douro regions were examined, with an assessment of their chemical properties conducted through conventional analyses (Paié‐Ribeiro et al. 2024). The key findings revealed that the dehydrated TPOC had the lowest moisture content (8%), whereas crude olive cake (COC) displayed high crude fat (14.5%) and varying protein levels (5.3%–7.3%). The NDF was notably high (>65%), indicating significant lignification. Furthermore, COC had the highest MUFA, whereas exhausted OC showed higher saturated and PUFAs. The phosphorus and phytic acid contents were comparable among most samples. These findings suggest that OC, particularly dry two‐phase OC, possesses beneficial properties for transport, conservation and utilization, presenting a viable option for reducing waste in the olive oil industry (Paié‐Ribeiro et al. 2024). Some researchers investigated the impact of OP on OM degradation through vermicomposting, utilizing *Eisenia fetida* earthworms for the process (Akpinar Borazan et al. 2024). Various biomass mixtures, including eggshells, cabbage and cattle manure, were amended with different OP concentrations (0%, 15%, 30% and 37.5%). After 45 days, analyses, such as nitrogen adsorption–desorption and FT‐IR, were conducted. Results indicated that the addition of OP enhanced biomass compactness and increased earthworm biomass by 31% at 37.5% OP. The study also noted a decrease in C/N ratios with lower OP levels, whereas higher levels led to increased C/N ratios due to elevated nitrogen values. Overall, OP was identified as a beneficial nutritional source for earthworms, promoting OM degradation (Akpinar Borazan et al. 2024). In another study, researchers conducted an investigation into the effects of a dietary intervention using OP oil rich in bioactive triterpenoids, specifically oleanolic and maslinic acid, during diet‐induced obesity in mice (Claro‐Cala et al. 2020). The pomace olive oil utilized in the study contained high concentrations of triterpenic acids (referred to as POCTA). The findings indicate that a 10‐week POCTA diet led to significant reductions in body weight, insulin resistance and adipose tissue inflammation, along with improvements in vascular function, despite a high caloric intake. This research highlights the potential of pomace olive oil as a functional food in combating obesity and contributes to the understanding of health benefits associated with the Mediterranean diet (Claro‐Cala et al. 2020). In general, olive BPs are abundant in compounds that exhibit antioxidant, antimicrobial, cardioprotective and anticancer properties, making them a promising candidate for the treatment of various diseases in both humans and animals. Simultaneously, their incorporation into the nutrition of ruminants facilitates the sustainable utilization of high‐value bioactive compounds within food chains, enhancing the quality of milk and meat while promoting consumer health. This approach does not adversely impact rumen function, metabolism or overall productivity.

**TABLE 5:** Investigation on the use of olive by‐products in animal diets.

Olive by‐product	Animal	Offered amount	Finding	Reference
Dried olive oil mill wastewater	Sarda ewes	0.1% and 0.2% of dietary polyphenol prepared from dried olive oil mill wastewater	· No significant effect on milk yield or composition · Decrease in milk urea content · Reduced somatic cell counts with increased polyphenols · Enhanced levels of vaccenic and rumenic acids with 0.2% polyphenols · Having nutraceutical value of new feed and environmental benefits · Alignment with circular economy principles through waste valorization	Cifuni et al. (2023)
Olive cake	In vitro protocol (rumen fluid collected from goat)	300 mg incubated in the glass syringes	· Two‐phase centrifugation of OC exhibited the lowest DM content, the highest nitrogen‐free extract and increased levels of tannins compared to mechanical press and three‐phase centrifugation · Mechanical press of OC demonstrated the lowest digestibility · The chemical composition of OC and its digestibility were also impacted by the extraction period and process	El Otmani et al. (2019)
Olive cake	Barki sheep	10% of DM	· Diet compositions were traditional concentrate (S1), non‐traditional concentrate (S2) with discarded dates and OC, supplemented with date palm frond (S3) · Higher rumen pH was observed in experimental diets supplemented with date palm frond (S3) compared to traditional concentrate (S1) and non‐traditional concentrate with discarded dates and OC (S2) · The S1 diet resulted in the highest TVFAs and rumen ammonia levels · Increased acetic and butyric acid proportions in S2 and S3; reduced propionic acid compared to S1 · *Methanobrevibacter* dominance in rumen methanogens, with numeric decline due to non‐traditional feed inclusion · Principal component analysis identified three distinct clusters based on fermentation parameters and methanogen abundance · Positive and negative correlations between methanogen genera and rumen metabolites noted	Rabee et al. (2021)
Sieved olive pulp	Rabbit	Replacement of basal diet with 20% and 25%	· Utilization of SOP improved live body weight, body weight gain and feed conversion ratio · Enhanced nutrient digestibility in supplemented groups · Higher dressing percentage in groups fed 20% and 25% SOP with enzyme and yeast combination · Increased total protein, albumin (A), globulin (G) and A/G ratio in treated groups · Elevated TAC, SOD and GPx in supplemented diets · Significant decrease in triglycerides, total cholesterol and MDA levels in treated groups · Improved economic efficiency in SOP‐supplemented diets compared to control · Exogenous enzymes and/or dry yeast supplementation enhance SOP's nutritional value and rabbit performance	Alderey et al. (2024)
Olive cake	Pig	20% of DM	· Significant differences in microbial abundance at the phylum, genus and species levels were identified using differential abundance analysis among experimental diets (control [C], 20% partially defatted OC [20PDOC] and 20% cyclone OC [20COC]) · Increased abundance of health‐promoting bacteria in 20PDOC and 20COC groups, including *Plactomycetota* and *Allisonella* · Higher concentrations of SCFAs in slurry from OC‐fed groups notably increased acetic, butyric, caproic and heptanoic acids in 20COC pigs · Positive correlations between specific bacteria (uncultured *Bacteroidales*, uncultured *Selenomonadaceae*) and energy digestibility · *Monoglobus* and *Desulfovibrio* positively correlated with total SCFAs, indicating significant impact on gut fermentation · Inclusion of OC in pig diets suggests potential to enhance gut microbiota composition and functionality, along with improved nutrient digestibility and fermentation patterns	Belloumi et al. (2024)
Olive pomace	Broiler chicks	1.5%, 3.5% and 4.5% of dietary DM	· There were no significant differences in productive traits (body weight, weight gain, feed intake and food conversion factor) among OP treatments and the control · Highlighted economic importance of olive	Habib et al. (2023)
Olive pomace	Dairy cow	15% of dietary DM	· The inclusion of OP (up to 15% DM basis) does not impact milk production or composition · Feed efficiency was maintained with the inclusion of OP in the livestock diet	Chaves et al. (2020)
Olive oil pomace	Dairy cow	8% of dietary DM	· OOP supplementation did not affect the DM intake, rumen degradability or milk production · Improvement in milk nutritional quality, along with increase in functional fatty acids (e.g., linoleic acid and conjugated linoleic acid) · Decrease in biohydrogenation rate of UFAs in the rumen · OOP reduced methane production potential · There were minimal changes in rumen microbiota, with certain bacteria increasing in abundance with OOP · OOP inclusion enhances milk quality without compromising rumen degradability or animal performance · OOP rich in polyphenols improves milk nutritional value	Scicutella et al. (2023)
Olive pomace	Dairy buffalo	7.5% and 15% of dietary DM	· Study focused on the effects of OP on Khuzestan dairy buffaloes’ production performance and nutrient digestibility · Experiment involved 10 lactating buffaloes with 3 treatments (control, 7.5% OP and 15% OP) · Increased milk fat and fat‐free solids in experimental treatments · No significant effect on DMI, protein production or milk pH · Blood parameters remained largely unaffected; however, cholesterol and alkaline phosphatase levels increased in specific treatments · Nutrient digestibility showed no significant changes due to treatments · Conclusion: OP can replace wheat flour in lactating buffalo diets without negative effects on production performance or nutrient digestibility · Recommendation to include up to 15% OP in the ration of milking buffaloes due to its reasonable price	Mohammadabadi et al. (2023)
Olive cake	Beef cattle	7.5% and 15% of dietary DM	· Evaluation of dietary partially destoned OC supplementation on performance and meat quality of Limousin bulls · 45 bulls divided into 3 groups: control (no supplementation), low OC (7.5%) and high OC (15.0%) · Increase in body weight, ADG, slaughter traits and intramuscular fat content with OC supplementation · Improvement in quality indices of carcass associated with OC inclusion · 15.0% OC reduced cooking loss and shear force · Increased unsaturated fatty acid content with higher OC inclusion	Chiofalo et al. (2020)
Olive cake	Damascus dairy goats	10% and 20% of dietary DM	· Evaluation of dietary inclusion of ensiled OC on milk yield and composition in Damascus dairy goats · 72 goats assigned to 3 iso‐nitrogenous and iso‐energetic diets: 0%, 10% and 20% OC for 42 days · There were no significant differences in milk yield, fat‐corrected milk, fat or protein yield among the groups treated with OC and the control group · Increase in milk fat percentage with higher OC inclusion rates · Increase in milk protein percentages in both OC groups, significantly in the 20% OC group · Reduced content of fatty acids (C4:0–C16:0) and enhanced MUFAs concentration in OC groups · Increased mammary expression of SLC2A1, VLDLR and FABP3 genes in OC groups · Elevated *SLC2A1* and *FASN* gene expression in adipose tissue of goats fed the OC20 diet	Neofytou et al. (2023)
Drum‐dried pitted olive pomace	Mice	10% and 20% of dietary DM	· Evaluation of health benefits of drum‐dried pitted OP pulp from first and second oil extraction in mice · Higher total soluble phenols in OP from first extraction compared to second extraction · Hydroxytyrosol identified as the main phenolic compound in OP · Lower weight gain observed in mice on OP diets compared to high and low‐fat control diets · High faecal protein levels in OP diets indicating poor protein retention, possibly due to phenolic binding · Reduced liver weight and adipose tissue in mice consuming high fat OP diets compared to high fat control diet · No significant effect of OP on blood glucose levels · Changes in gut microbiota: decreased *actinobacteria* and increased *bacteroidetes* in OP diets, correlating with reduced body fat and weight	Inzunza‐Soto et al. (2021)

Abbreviations: ADG, average daily gain; DM, dry matter; DMI, dry matter intake; GPx, glutathione peroxidase; MDA, malondialdehyde; MUFAs, mono‐unsaturated fatty acids; OC, olive cake; OOP, olive oil pomace; OP, olive pomace; SCFAs, short‐chain fatty acids; SOD, superoxide dismutase; SOP, sieved olive pulp; TAC, total antioxidant capacity; TVFAs, total volatile fatty acids; UFAs, unsaturated fatty acids.

The processing of olives into oil affects olive meal by altering its chemical composition, particularly its phenolic content, fibre and antioxidant properties (Safarzadeh Markhali 2021). Enhanced extraction techniques can improve the nutritional value and functional properties of olive meal, making it a valuable BP rich in bioactive compounds that can be utilized in animal feed, food products or nutraceuticals (Safarzadeh Markhali 2021). It has been shown that ensiling olive BPs with different additives and additions to diets of animals can improve their performance (Taheri et al. 2012; Abid et al. 2020; Symeou et al. 2021).

## 8 Tomato BPs

**FIGURE 7:**
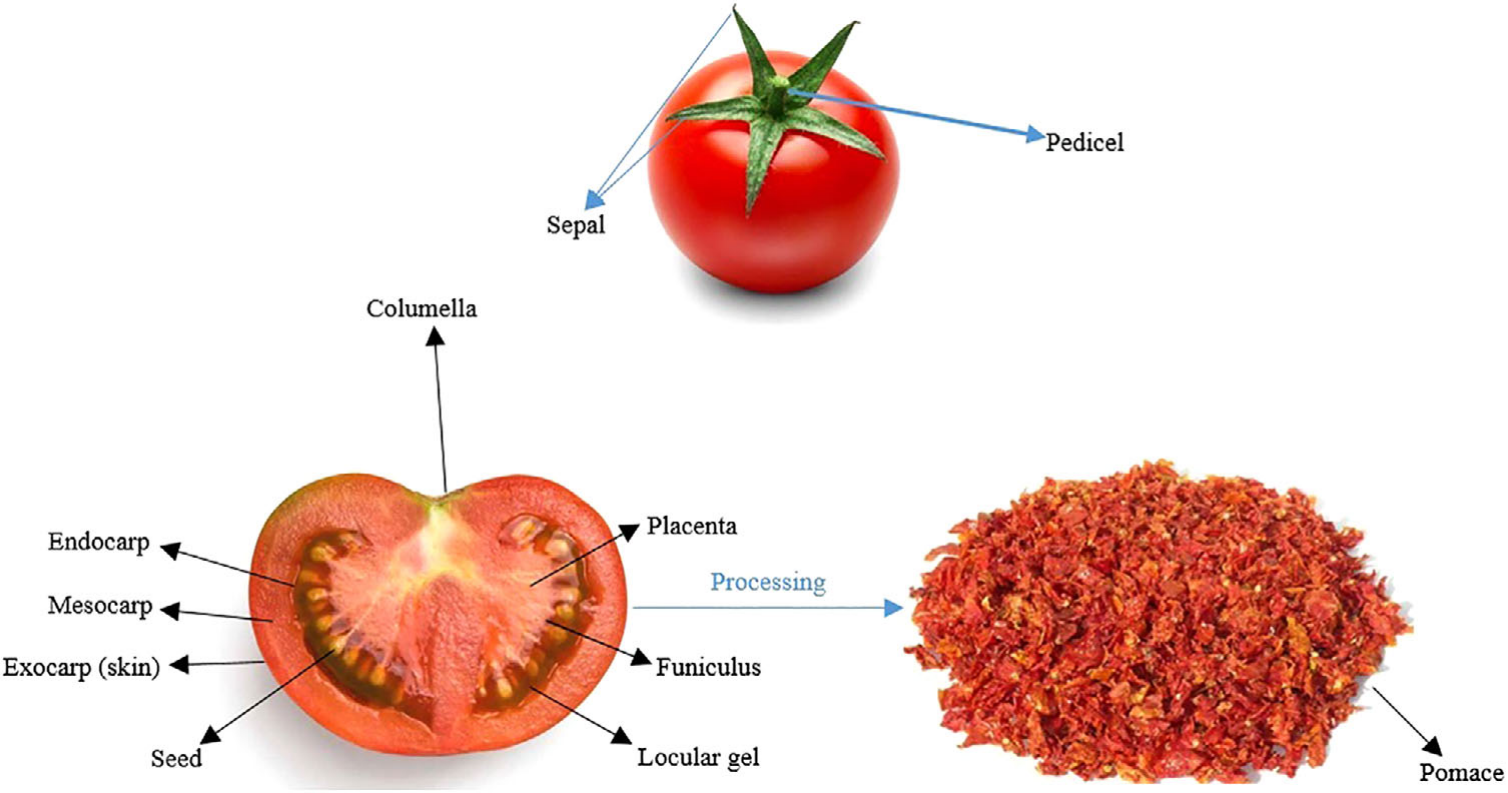
A schematic of the parts of the tomato and its pomace.

Tomatoes (*Solanum lycopersicum* L.) are among the most extensively cultivated vegetables worldwide. It is a significant edible plant that is rich in bioactive compounds, including protein, fibre, carotenoids and pectin (Kiralan and Ketenoglu 2022). These compounds are recognized for their protective properties against various human diseases. Tomato pulp, also referred to as tomato pomace, is an agricultural BP derived from the leftover pulp produced during the manufacturing of tomato paste and sauce. It constitutes approximately 2%–10% of the total weight of fresh tomatoes (Hafez 2024). Tomatoes are processed into a variety of products, such as ketchup, paste, sauce, puree, soup, juice and canned tomatoes, resulting in substantial waste generation (Kiralan and Ketenoglu 2022). A schematic of the parts of the tomato and its pomace is illustrated in Figure 7. In this figure, the various parts of the tomato, including columella, endocarp, mesocarp, exocarp (skin), seed, locular gel, funiculus, placenta, pedicel and sepal, are highlighted. Each of these parts can serve as valuable nutritional waste for animal feed after the dehydration process of the tomato. This waste is a valuable source of bioactive compounds that can enhance human health. More than half of the composition of tomato waste consists of fibre, sugars and proteins, with carotenoids serving as important minor components. Lycopene, in particular, is a notable carotenoid that offers significant health benefits (Laranjeira et al. 2022). Lycopene, a carotenoid, is responsible for the red colouration of tomatoes. The primary degradation pathways of lycopene during the processing of tomatoes include oxidation and isomerization. Oxidation predominantly occurs at low pH levels, particularly in the presence of light and oxygen during non‐thermal processing methods such as cutting, grinding and storage (Laranjeira et al. 2022). TP, a BP of tomato processing, includes the peel, seeds and small amounts of pulp (Kiralan and Ketenoglu 2022). These components are often repurposed in various products. Dried tomato waste can be used as animal feed and as an ingredient in meat products, whereas the seeds can be added to baked goods and fermented cereals. Additionally, tomato seeds are rich in oils that contain high levels of carotenoids, contributing to the oxidative stability of the oil. Tomato seed oil offers promising uses beyond culinary purposes, particularly in the production of biodiesel (Kiralan and Ketenoglu 2022). The predominant SFA identified in TP is palmitic acid (Lu et al. 2022). TP serves as a valuable natural source of lipids (5%–10%), proteins (10%–20%) and dietary fibre (60%–70%) (Hafez 2024). TP is notably rich in minerals, especially calcium, phosphorus, magnesium, sodium and potassium. Among these, potassium exhibited the highest concentration, ranging from 303 to 1125 g/kg, with a mean value of 835.5 g/kg. Sodium levels varied between 47.2 and 191.7 g/kg, yielding an average of 97.7 g/kg (Lu et al. 2022). The calcium content ranged from 76.4 to 160.0 g/kg; however, the average was reported as 170.3 g/kg, which may be attributed to a potential measurement discrepancy. Phosphorus was represented by a single measurement of 219.7 g/kg. Magnesium levels showed considerable variation, ranging from 3.1 to 251.1 g/kg, yet only one measurement was as low as 3.1 g/kg (Lu et al. 2022). Contrarily, other investigations reported magnesium concentrations exceeding 100 g/kg, averaging around 149.7 g/kg. The quantities of iron and zinc were relatively low, with iron levels varying from 1.5 to 11 g/kg and averaging 3.7 g/kg, whereas zinc levels ranged from 0.5 to 6.3 g/kg, with an average of 3.4 g/kg (Lu et al. 2022). The predominant essential amino acids identified in the analysis were phenylalanine, leucine, arginine and lysine (Lu et al. 2022). Ensuring the quality of final processed products is a key priority for the food processing industry; therefore, it is crucial to take into account the factors that affect the raw materials (Laranjeira et al. 2022). Because the quality of BPs is directly linked to the processing techniques and conditions used, it is crucial for the tomato processing industry to receive proper guidance to optimize outcomes. Consequently, variations in the chemical composition and nutritional value of tomato BPs can be attributed to different processing methods. For instance, the concentration of lycopene has been demonstrated to increase following specific processing methods, such as heat treatment (Laranjeira et al. 2022). Thus, employing appropriate heating techniques can greatly enhance the retention of nutrients in both tomato‐derived products and the nutritional quality of the TP. Additionally, it contains bioactive compounds such as lycopene and beta‐carotene, which are recognized for their potent antioxidant properties (Hafez 2024). β‐carotene is a carotenoid that gives the characteristic orange colour to many fruits and vegetables, making it the second most abundant coloured carotenoid present in tomatoes (Laranjeira et al. 2022). It is extensively utilized in the food industry as an additive, particularly as a colouring agent. The primary significance of β‐carotene for human health is attributed to its antioxidant properties and its function as a precursor to vitamin A (Laranjeira et al. 2022). Given the abundance of tomato residues, they present a valuable opportunity to be incorporated as agricultural inputs in animal and poultry nutrition. Tomatoes contain lycopene, folate, vitamin C, vitamin A, phenols and flavonoids (Laranjeira et al. 2022). Phenolic compounds are significant phytochemicals renowned for their potent antioxidant activity, found in a wide variety of fruits and vegetables. These compounds can be categorized into two principal groups: polyphenols and phenolic acids. Their antioxidant capacity is chiefly attributed to their ability to scavenge reactive species through electronic and atomic exchanges (Laranjeira et al. 2022). In tomatoes, notable phenolic compounds include flavonoids, phenolic acids and tannins. The phenolic content in tomatoes is greatly influenced by factors such as agricultural practices, genotype and storage conditions. In many fruits and vegetables, phenolic compounds are predominantly associated with cell walls. Thus, certain processing methods, particularly those that disrupt cell membranes, can enhance the bioavailability of phenolic compounds (Laranjeira et al. 2022). The TPCs varied between 94.5 and 213.4 mg GAE/g, with a mean value of 161.8 mg GAE/g (Lu et al. 2022). The TFC ranged from 30.6 to 378.7 mg QE/g, averaging 124.4 mg QE/g. Lycopene levels were found to range from 36.7 to 50.2 g/kg, with an average of 44.6 g/kg (Lu et al. 2022). The lipids present in tomatoes are primarily in the form of UFAs and are composed of glucose, fructose and essential amino acids (Lamtar Mohammadi et al. 2021). Additionally, TP contains various phytochemicals, including flavanones such as naringenin and glycosylated derivatives, as well as flavonols like quercetin, rutin and glycosylated kaempferol derivatives (Aminifard and Kiani 2023). Some researchers evaluated agricultural wastes as non‐fasting methods for inducing moulting in laying hens. Five treatments were tested: feed withdrawal, apple peel waste, carrot pomace, TP and zinc oxide (20 g/kg) (Heidari Safar et al. 2024). Results showed reduced feed intake in pomace groups without affecting body weight loss. The zinc oxide group exhibited the lowest egg production, whereas the apple peel and TP treatments enhanced production during the resting period. Pomace groups had lower heterophil to lymphocyte ratios and improved yolk colour. Blood tests revealed lower triglycerides in pomace groups, with higher cholesterol and MDA in the feed withdrawal group. Ovary and oviduct weights were lower in pomace groups compared to zinc oxide. In conclusion, they noted that TP and apple peel waste are effective alternatives to feed withdrawal for induced moulting (Heidari Safar et al. 2024). Moreover, it assessed the nutritional value of tomato pulp in broiler diets utilizing a multivalent enzyme (Lotfi et al. 2021). Broiler chicks were fed different diets: control (no tomato pulp or enzyme), 5% TP without enzyme, 5% TP with 0.2% enzyme, 10% TP without enzyme and 10% TP with 0.2% enzyme for 42 days. The results indicated that chicks on enzyme‐supplemented diets experienced greater weight gain compared to those on diets without the enzyme. The 5% TP with enzyme increased empty gizzard weight compared to control. Serum lipids were unaffected, but TP diets raised high‐density lipoprotein (HDL) levels. Villi height in the jejunum and ileum was higher in the 5% TP with enzyme group, whereas the highest villi were observed in the 5% TP without enzyme. The 10% TP diet reduced feed costs per unit of live weight gain by 5%. In general, they demonstrated that using multi‐enzyme improves the nutritional value of TP in broiler diets up to 10% (Lotfi et al. 2021). A study was conducted to assess the nutritional value of TP as an agricultural BP for animal nutrition (Aminifard and Kiani 2023). The research aimed to compare the gas production and digestibility of TP with WB in vitro. They determined the chemical composition and gas production parameters of both materials and substituted TP at levels of 0%, 5.5%, 5.7% and 10% for WB in the diets of fattening lambs. Results indicated that TP contained higher CP and insoluble fibres compared to WB. However, it exhibited lower gas production potential and digestibility of DM and OM. Nonetheless, the production of microbial protein was greater when TP was included in the diet, and increasing the amount of TP further boosted microbial protein production (Aminifard and Kiani 2023). Overall, they noted that incorporating up to 10% TP in lamb diets as a substitute for WB did not negatively impact ruminal fermentation parameters and could be a cost‐effective alternative (Aminifard and Kiani 2023). Additionally, the study explored the use of TP as a substitute feed ingredient in poultry diets, highlighting its potential advantages for breeding quails (Malek et al. 2021). The study involved 160 Japanese quails divided into four treatment groups (0%, 4%, 8% and 12% TP) over 5 weeks, assessing the impact on progeny performance, organ weights, chick quality and MDA levels in meat. Results indicated that incorporating TP did not significantly affect feed consumption, weight gain or feed conversion ratios. However, quails fed 8% TP exhibited numerically higher feed intake. The quality of newly hatched chicks was notably improved in those from quails fed 8% TP, with better overall appearance and lower MDA content in breast meat compared to controls. Conversely, a higher inclusion level (12%) negatively impacted chick quality, likely due to increased dietary fibre reducing nutrient bioavailability. Overall, the study concluded that although TP does not adversely affect progeny performance, an optimal inclusion level of 8% enhances antioxidant status and chick quality (Malek et al. 2021). Moreover, a study was conducted to assess the nutritional and digestibility values of TP both before (TP1) and after the oil extraction (TP2) process (Aminifard et al. 2022). In this study, the lycopene content of TP and its ruminal disappearance rate were measured, using a completely randomized design to evaluate gas production in vitro. The study utilized fistulated cows to determine the ruminal degradation parameters of TP over various incubation times. The results indicated that TP1 contained 168 mg/kg DM of lycopene, whereas TP2 had higher levels of CP, NDF and ADF than TP1. The TP2 also exhibited increased gas production, OM digestibility and short‐chain fatty acid production, alongside reduced ammonia nitrogen levels compared to TP1. The ruminal degradability of lycopene was found to be approximately 30%, suggesting that over 70% of lycopene bypasses the rumen. In general, the findings conclude that de‐oiled TP possesses (TP2) favourable nutritional values for ruminant nutrition (Aminifard et al. 2022). Growth and reproductive performance are critical parameters within animal production systems, serving as key indicators for assessing animals’ responses to feed, environmental conditions and various production methods. The findings indicated that the body weight, feed intake and feed conversion ratio of broiler chickens were not significantly influenced by the inclusion of TP fermented with *A. niger* (Gungor et al. 2024). Consistent with this finding, a group of researchers reported no significant impact on growth performance when dietary TP was supplemented at levels of 20 and 50 g/kg, respectively (Rezaeipour et al. 2012; Faryabidoust et al. 2013). This lack of impact may stem from the high inclusion rate of TP in the chickens’ diets, as its elevated fibre and lignin content could impair digestibility, nutrient absorption and utilization, ultimately diminishing productivity. Conversely, an increase in body weight was observed among broilers when supplemented with tomato puree at levels of 5 and 10 g/kg (Selim et al. 2013). The variations between these findings could be attributed to the use of different components of tomato BPs and the comparatively lower inclusion levels utilized in the diet. Recent studies have focused on the suitability of substitute protein sources in aquaculture diets. In this regard, a study was conducted to investigate the growth performance and blood parameters of common carp (*Cyprinus carpio*) fed diets supplemented with tomato paste BP extract (TPE) (Kesbiç et al. 2022). The research included five diets with varying TPE concentrations (0%, 0.5%, 1%, 2% and 5%) over a 60‐day period involving 300 fish (Kesbiç et al. 2022). Results indicated significant enhancements in relative and specific growth rates, alongside a decrease in feed conversion ratios. Haematological analyses revealed that TPE significantly improved erythrocyte counts, haemoglobin content and haematocrit levels. Biochemical assessments showed that diets containing 1% or more TPE significantly lowered serum glucose, cholesterol and triglyceride levels while increasing total protein, albumin and globulin. The study concluded that a 2% TPE extract could serve as an effective growth promoter in common carp diets without adversely affecting blood parameters (Kesbiç et al. 2022). Similarly, a study evaluated the effects of adding TP, with or without enzymes and amino acids, into the diets of *Nile tilapia* over an 8‐week period, focusing on growth performance, digestive enzymes, histological parameters and liver gene expression (Hafez et al. 2024). Five experimental diets were developed, all maintaining a protein content of 28%, with varying combinations of TP, lysine, methionine and gallizyme. The study found that the groups receiving enzyme supplementation, particularly those with amino acids, showed significant improvements in growth performance metrics, including body weight gain and feed conversion ratio, compared to the control. Furthermore, enzyme supplementation was associated with increased intestinal villi length and goblet cell numbers, whereas liver expression levels of IGF‐1 and GH were significantly elevated in the enzyme and amino acid groups. Overall, the inclusion of enzymes, with or without amino acids, positively influenced both growth parameters and gene expression in *N. tilapia* (Hafez et al. 2024). The inclusion of silages made from surplus tomatoes and olive BPs, along with sunflower oil, in the diet of dairy goats will sustain rumen fermentation and milk production while improving the fatty acid profile of the milk (Arco‐Pérez et al. 2017). Hence, two experiments were conducted to evaluate the use of olive and tomato silages as partial replacements for conventional forage in the diets of lactating Murciano‐Granadina goats. In experiment 1, the inclusion of olive BPs silage and tomato surplus silage (TSD), supplemented with sunflower oil, led to higher fat intake, nutrient digestibility and specific fatty acid profiles in the goats’ milk compared to the control diet (Arco‐Pérez et al. 2017). Additionally, TSD resulted in lower methane production. Experiment 2 confirmed these findings over a longer period (90 days) with more animals, again showing increased DMI and changes in fatty acid profiles without affecting milk yield. The study suggests that substituting traditional forage with local BPs can enhance the energy balance in dairy goats while maintaining overall nutritional efficiency (Arco‐Pérez et al. 2017). Similarly, an assessment was conducted to determine whether the inclusion of BPs, such as GP, pomegranate residues, OC and TP, in the diets of dairy ruminants could improve the nutritional quality of dairy products without adversely affecting production efficiency (Correddu et al. 2023). The results indicated that partial replacement of concentrates with BPs generally does not reduce milk yield or composition, though high doses may decrease yield by 10%–12% (Correddu et al. 2023). Conversely, positive changes in milk fatty acid profiles were observed with the inclusion of BPs (5%–40% of DM), supporting economic and environmental sustainability while lessening food competition between humans and animals (Correddu et al. 2023). They demonstrated that enhancing the nutritional quality of milk fat derived from BPs offers considerable commercial advantages for the dairy industry (Correddu et al. 2023). In a study, researchers substituted 10% of the corn silage with tomato paste silage in the diets of dairy cows (Tuoxunjiang et al. 2020). It was observed that, although no significant changes were detected in milk production or composition, there was an increase in DMI, digestibility and concentrations of vitamins in the milk (Tuoxunjiang et al. 2020). This dietary modification also led to elevated levels of total cholesterol, HDL, cholesterol, serum aspartate aminotransferase, antioxidant capacity and improved immune performance.

Similarly, analogous findings were reported when fermented tomato paste was used as a substitute for soybean meal, highlighting an increase in DMI and 4% fat‐corrected milk production (Zhao et al. 2012). However, no significant impact was observed on average milk production, feed conversion ratio or the contents of milk fat, protein and total solids (Zhao et al. 2012). Importantly, this approach resulted in reduced feed costs alongside increased economic benefits. In contrast, differing outcomes were reported, indicating no significant differences in average daily DMI or the nutrient consumption of DM, OM, NDF and ADF digestibility, as well as no variations in faecal and rumen pH values among the cows fed silage TP (Tahmasbi et al. 2004). Furthermore, the nutritional variability of TP for ruminants was assessed, along with its impact on in vitro fermentation when incorporated into high‐concentrate diets (Marcos et al. 2019). A total of 12 TP samples collected weekly from 2 processing plants were analysed for chemical composition, fermentation and digestibility. The results indicated that TP exhibits consistent chemical composition across plants and minimal variation over time, characterized by low DM but high NDF, CP and EE. The fermentation of TP in the rumen occurred rapidly, demonstrating significant protein degradability, but exhibited low intestinal digestibility. Replacing soybean meal and barley straw with dried TP in diets enhanced fermentation rates and volatile fatty acid production while reducing ammonia nitrogen concentrations; however, methane production remained unaffected. Ultimately, TP can be included in high‐concentrate diets at levels up to 180 g/kg, maintaining effective rumen fermentation (Marcos et al. 2019). TP has also been utilized as a feed ingredient for rabbits. In this regard, a study involved 120 New Zealand White rabbits to evaluate the effects of dried tomato pomace powder (DTPP) supplementation on their performance, blood metabolites, carcass traits and meat quality from 5 to 13 weeks of age (Hassan et al. 2024). Four dietary treatments were applied: a control diet and diets supplemented with 0.5%, 1.0% and 1.5% DTPP. Results indicated that rabbits on the 1.5% DTPP diet experienced the highest growth rates and best feed conversion ratios despite lower feed intake. Supplementation improved meat quality by enhancing fatty acid profiles, reducing fat deposition and lowering cholesterol levels. Notably, diets with DTPP elevated beneficial fatty acids and health indices in the meat. Overall, DTPP up to 1.5% positively impacted growth performance and meat quality in rabbits (Hassan et al. 2024). Similarly, the impact of DTTP on the performance and digestibility of growing rabbits was investigated (Grioui et al. 2019). Fifty‐four 6‐week‐old rabbits were divided into three groups and fed diets with 0% (as control), 10% (DTP‐10) and 20% (DTP‐20) DTP for 6 weeks. The digestibility trial revealed that although DM and EE digestibility remained consistent across groups, CP digestibility significantly increased with higher DTP inclusion. No significant differences were observed in weight gain, ADG, feed intake or feed conversion ratio among groups. Notably, the 20% DTP group exhibited the lowest mortality rate. Additionally, DTP supplementation reduced production costs and enhanced net revenue (Grioui et al. 2019). The study concludes that incorporating up to 20% DTP in rabbit diets improves protein digestibility. In general, the high nutritional profile of TP supports its effective use as a value‐added component in animal feed. Incorporating TP into animal diets can enhance feed intake and growth performance, increase the levels of PUFAs and *n*‐3 fatty acids in meat and improve the colour, nutritional quality and juiciness of the meat (Grioui et al. 2019). Additionally, TP supplementation can boost the immunity and antioxidant capabilities of animals, as well as enhance sperm quality. Furthermore, the reduction of rumen pH and methane emissions in ruminants fosters the fermentation activity of rumen microorganisms, leading to improved economic efficiency. To avoid any negative effects, it is crucial to follow the recommended supplementation levels of TP: no more than 15% for poultry, 40% for goats, 15% for cattle and 60% for rabbits (Lu et al. 2022). Ensiling promotes the preservation of feed quality by facilitating anaerobic fermentation, which enhances nutrient availability and reduces losses from spoilage, ultimately contributing to improved livestock health and productivity. In this context, it has been reported that the ensiling of tomato BPs enhances their quality, subsequently improving animal performance following their consumption (Fayed 2019; Tuoxunjiang et al. 2020). Furthermore, the ensiling of wet tomato pomace with dried molasses sugar beet pulp significantly improved its silage quality by increasing DM, ME and IVOMD, while reducing CP, fibre contents and ammonia nitrogen levels, without the presence of butyric acid (Sargın and Denek 2017).

## 9 Conclusion

The integration of agricultural f into ruminant diets presents a valuable opportunity to enhance sustainability in animal nutrition. BPs from pomegranate, grape, pistachio, saffron, raisins, olives and tomatoes offer significant nutritional benefits, improving feed efficiency and animal performance while decreasing reliance on conventional feed sources. Utilizing these BPs minimizes agricultural waste and contributes to a circular economy by recycling nutrients back into the food system, addressing environmental concerns linked to livestock farming. Furthermore, incorporating agricultural waste can provide economic benefits for farmers by lowering feed costs without compromising animal health or productivity. However, key challenges include the need for further research on optimal incorporation levels and the potential anti‐nutritional factors in some agricultural wastes. To facilitate the adoption of these practices, stakeholders should engage in specific recommendations: Farmers should document the effects of various agricultural BPs on livestock to build a knowledge base on best practices; policymakers can support this transition by offering incentives, such as subsidies or grants, for sustainable practices; and researchers should investigate the nutritional profiles and anti‐nutritional factors of different BPs, providing evidence‐based guidelines for their safe use in ruminant diets. By strategically utilizing agricultural BPs, we can enhance food security and promote sustainable agricultural practices, fostering a more environmentally friendly livestock sector. Embracing innovative methods and encouraging collaboration across sectors will enable the agricultural community to contribute to a sustainable future while meeting the growing global demand for livestock products. This article identifies critical areas for future research and emphasizes the need to prioritize them effectively to guide subsequent efforts. Expanding the call for interdisciplinary approaches, including specific technologies like artificial intelligence in feed formulation and advanced waste processing techniques, would further enhance the integration of agricultural BPs into ruminant diets. Additionally, incorporating regulatory and policy considerations is vital to address potential barriers to the adoption of these innovative feeding strategies, ultimately laying a stronger foundation for future studies in this important field of livestock nutrition.

## Author Contributions

All the contents and information of the article have been collected and organized by Mohsen Kazemi. Additionally, the article was edited by Mohsen Kazemi.

## Ethics Statement

The author verifies that the journal's ethical standards, as detailed on the journal's author guideline page, have been complied with. As this is a review article lacking original research data, ethical approval was not necessary.

## Conflicts of Interest

The author declares no conflicts of interest.

## Data Availability

The data used in this review are derived from reputable sources and do not include any original data from the author.

## References

[vms370321-bib-0001] Abarghuei, M. J. , Y. Rouzbehan , A. Z. M. Salem , and M. J. Zamiri . 2020. “Effects of Pomegranate Peel Extract on Ruminal and Post‐Ruminal *In Vitro* Degradation of Rumen Inoculum of the Dairy Cow.” Animal Biotechnology 32, no. 3: 366–374. 10.1080/10495398.2020.1727492. 32057286

[vms370321-bib-0002] Abbeddou, S. , B. Rischkowsky , M. E.‐D. Hilali , M. Haylani , H. D. Hess , and M. Kreuzer . 2014. “Supplementing Diets of Awassi Ewes With Olive Cake and Tomato Pomace: On‐Farm Recovery of Effects on Yield, Composition and Fatty Acid Profile of the Milk.” Tropical Animal Health and Production 47, no. 1: 145–152. 10.1007/s11250-014-0699-x. 25326442

[vms370321-bib-0003] Abd El‐Aziz, A. , A. Elfadadny , M. Abo Ghanima , et al. 2024. “Nutritional Value of Oregano‐Based Products and Its Effect on Rabbit Performance and Health.” Animals 14, no. 20: 3021. 10.3390/ani14203021. 39457951 PMC11505053

[vms370321-bib-0005] Abdulzahrah, A. S. , and A. A. M. Al‐Wazeer . 2022. “Effects of Pomegranate Peel and Laurel Bay Leaves on Growth Performance and Blood Parameters in Growing Lambs.” AIP Conference Proceedings 2547: 020035. 10.1063/5.0112491.

[vms370321-bib-0006] Abid, K. , J. Jabri , H. Ammar , et al. 2020. “Effect of Treating Olive Cake With Fibrolytic Enzymes on Feed Intake, Digestibility and Performance in Growing Lambs.” Animal Feed Science and Technology 261: 114405. 10.1016/j.anifeedsci.2020.114405.

[vms370321-bib-0007] Abu Hafsa, S. H. , G. Centoducati , A. A. Hassan , A. Maggiolino , M. Elghandour , and A. Z. M. Salem . 2024. “Effects of Dietary Supplementations of Vitamin C, Organic Selenium, Betaine, and Pomegranate Peel on Alleviating the Effect of Heat Stress on Growing Rabbits.” Animals 14, no. 6: 950. 10.3390/ani14060950. 38540048 PMC10967313

[vms370321-bib-0008] Abu Hafsa, S. H. , and S. A. Ibrahim . 2017. “Effect of Dietary Polyphenol‐Rich Grape Seed on Growth Performance, Antioxidant Capacity and Ileal Microflora in Broiler Chicks.” Journal of Animal Physiology and Animal Nutrition 102, no. 1: 268–275. 10.1111/jpn.12688. 28295656

[vms370321-bib-0009] Agricultural Statistics . 2017. Ministry of Agriculture Jihad, Iran. Vol. 2. Agricultural Statistics.

[vms370321-bib-0010] Ahmadi Kohanali, R. , S. J. Hosseini‐vashan , M. Mojtahedi , and H. Sarir . 2022. “Effects of Kallequchi Pistachio Green Hull (*Pistacia vera*) and Its Processed With Urea on Performance, Immune Response, Blood Biochemical Indices, and Jejunal Morphology in Broiler Chickens.” Iranian Journal of Applied Animal Science 14, no. 3: 379–398.

[vms370321-bib-0011] Ahmadi, M. , M. Yari , and M. Hedayati . 2022. “Effect of Graded Substitution of Barley Grain With Raisin Waste in Diet Contained Low Quality Forage on Growth Performance, Blood Metabolites and Nutrient Digestibility of Growing Ram Lamb.” Iranian Journal of Applied Animal Science 15, no. 3: 539–545.

[vms370321-bib-0012] Ahmed, M. G. , S. Z. El‐Zarkouny , A. A. Al‐Sagheer , and E. A. Elwakeel . 2025. “Co‐Ensiling Pomegranate (*Punica granatum* L.) Peels and Molasses With Berseem (*Trifolium alexandrinum* L.) Alters Fermentation Quality, Nutrient Composition, Ruminal Fermentation and Methane Production in Buffalo Bulls *In‐Vitro* .” Tropical Animal Health and Production 57, no. 1: 1–15. 10.1007/s11250-024-04259-6. PMC11711153 39779523

[vms370321-bib-0013] Ain, H. B. U. , T. Tufail , S. Bashir , et al. 2023. “Nutritional Importance and Industrial Uses of Pomegranate Peel: A Critical Review.” Food Science & Nutrition 11, no. 6: 2589–2598. 10.1002/fsn3.3320. 37324891 PMC10261788

[vms370321-bib-0014] Akbari Shooshood, M. , J. Rezaei , M. Ayyari Noushabadi , and Y. Rouzbehan . 2024. “Mechanism of Positive Phytobiotic Effects of Saffron Petals on Energy and Nitrogen Metabolism and Antioxidant Health of Sheep Rumen *In Vitro* .” Journal of Saffron Research 12, no. 1: 65–79.

[vms370321-bib-0015] Akhlaghi, B. , E. Ghasemi , M. Alikhani , M. Hosseini Ghaffari , and A. Razzaghi . 2022. “Effects of Supplementing Pomegranate Peel With Fatty Acid Sources on Oxidative Stress, Blood Metabolites, and Milk Production of Dairy Cows Fed High‐Concentrate Diets.” Animal Feed Science and Technology 286: 115228. 10.1016/j.anifeedsci.2022.115228.

[vms370321-bib-0016] Akpinar Borazan, A. , L. Değirmenci , and Ö. Cumhur Değirmenci . 2024. “Investigation of the Effects of Waste Olive Pomace on Vermicompost.” Bilge International Journal of Science and Technology Research 8, no. 2: 104–114. 10.30516/bilgesci.1460055.

[vms370321-bib-0017] Akram, M. Z. , and S. Y. Fırıncıoğlu . 2019. “The Use of Agricultural Crop Residues as Alternatives to Conventional Feedstuffs for Ruminants: A Review Title.” Eurasian Journal of Agricultural Research 3, no. 2: 58–66. https://dergipark.org.tr/en/pub/ejar/issue/50318/632177.

[vms370321-bib-0018] Akuru, E. A. , C. E. Oyeagu , T. C. Mpendulo , F. Rautenbach , and O. O. Oguntibeju . 2020. “Effect of Pomegranate (*Punica granatum* L) Peel Powder Meal Dietary Supplementation on Antioxidant Status and Quality of Breast Meat in Broilers.” Heliyon 6, no. 12: e05709. 10.1016/j.heliyon.2020.e05709. 33364487 PMC7750561

[vms370321-bib-0019] Alderey, A.‐A. A. , N. E. M. El‐Kassas , E. A. Hussein , et al. 2024. “Impacts of Enzymes and Probiotic in Improving the Utilization of Sieved Olive Pulp Meal in Growing Rabbit Diets.” Journal of Advanced Veterinary and Animal Research 11, no. 1: 161–170. 10.5455/javar.2024.k761. 38680804 PMC11055591

[vms370321-bib-0020] Alfaia, C. M. , M. M. Costa , P. A. Lopes , J. M. Pestana , and J. A. M. Prates . 2022. “Use of Grape By‐Products to Enhance Meat Quality and Nutritional Value in Monogastrics.” Foods (Basel, Switzerland) 11, no. 18: 2754. 10.3390/foods11182754. 36140881 PMC9497639

[vms370321-bib-0021] Alipour, D. , and Y. Rouzbehan . 2007. “Effects of Ensiling Grape Pomace and Addition of Polyethylene Glycol on *In Vitro* Gas Production and Microbial Biomass Yield.” Animal Feed Science and Technology 137, no. 1–2: 138–149. 10.1016/j.anifeedsci.2006.09.020.

[vms370321-bib-0022] Alipour, F. , S. A. Vakili , M. Danesh Mesgaran , and S. Ebrahimi . 2016. “Determine the Chemical Composition and Nutritional Value of Saffron Petals Using Method in Sacco.” In 7th Congress in Animal Science. Tehran University, Iran.

[vms370321-bib-0023] Alipour, F. , A. Vakili , M. Danesh Mesgaran , and H. Ebrahimi . 2019. “The Effect of Adding Ethanolic Saffron Petal Extract (SPE) and Vitamin E on Growth Performance, Blood Metabolites and Antioxidant Status in Baluchi Male Lambs.” Asian‐Australasian Journal of Animal Sciences 32, no. 11: 1695–1704. 10.5713/ajas.18.0615. 31011009 PMC6817774

[vms370321-bib-0024] Amato, A. , L. Liotta , C. Cavallo , et al. 2024. “Effects of Feeding Enriched‐Olive Cake on Milk Quality, Metabolic Response, and Rumen Fermentation and Microbial Composition in Mid‐Lactating Holstein Cows.” Italian Journal of Animal Science 23, no. 1: 1069–1090. 10.1080/1828051x.2024.2381736.

[vms370321-bib-0025] Aminifard, Z. , and A. Kiani . 2023. “Determination of Fermentation and Digestibility Parameters of Tomato Pomace in Comparison With Wheat Bran Under *In Vitro* Conditions.” Animal Science and Research 33, no. 2: 93–105.

[vms370321-bib-0026] Aminifard, Z. , A. Kiani , and A. Azarfar . 2022. “Determination of Nutritional Value and Digestibility of Tomato Pomace Before and After Oil Extraction *In Vitro* and Evaluation of Ruminal Disappearance of Lycopene *In Situ* .” Journal of Animal Production 24, no. 4: 441–452.

[vms370321-bib-0027] Ampem, G. 2017. “Quality Attributes of Pomegranate Fruit and Co‐Products Relevant to Processing and Nutrition.” Published May. https://scholar.sun.ac.za.

[vms370321-bib-0028] Angaji, L. , M. Souri , and M. M. Moeini . 2011. “Deactivation of Tannins in Raisin Stalk by Polyethylene Glycol‐600: Effect on Degradation and Gas Production *In Vitro* .” African Journal of Biotechnology 10, no. 21: 4478–4483.

[vms370321-bib-0029] Antunović, Z. , J. Novoselec , Ž Klir Šalavardić , et al. 2024. “The Effect of Grape Seed Cake as a Dietary Supplement Rich in Polyphenols on the Quantity and Quality of Milk, Metabolic Profile of Blood, and Antioxidative Status of Lactating Dairy Goats.” Agriculture 14, no. 3: 479. 10.3390/agriculture14030479.

[vms370321-bib-0030] Arapoglou, D. , C. Eliopoulos , G. Markou , I. Langousi , G. Saxami , and S. A. Haroutounian . 2024. “Nutritional Upgrade of Olive Mill Stone Waste, Walnut Shell and Their Mixtures by Applying Solid State Fermentation Initiated by *Pleurotus ostreatus* .” Scientific Reports 14, no. 1: 13446. 10.1038/s41598-024-64470-1. 38862766 PMC11166993

[vms370321-bib-0031] Arco‐Pérez, A. , E. Ramos‐Morales , D. R. Yáñez‐Ruiz , L. Abecia , and A. I. Martín‐García . 2017. “Nutritive Evaluation and Milk Quality of Including of Tomato or Olive By‐Products Silages With Sunflower Oil in the Diet of Dairy Goats.” Animal Feed Science and Technology 232: 57–70. 10.1016/j.anifeedsci.2017.08.008.

[vms370321-bib-0032] Arjeh, E. , H.‐R. Akhavan , M. Barzegar , and Á. A. Carbonell‐Barrachina . 2020. “Bio‐Active Compounds and Functional Properties of Pistachio Hull: A Review.” Trends in Food Science & Technology 97: 55–64. 10.1016/j.tifs.2019.12.031.

[vms370321-bib-0033] Asdaq, S. M. B. , and M. N. Inamdar . 2009. “Potential of *Crocus sativus* (Saffron) and Its Constituent, Crocin, as Hypolipidemic and Antioxidant in Rats.” Applied Biochemistry and Biotechnology 162, no. 2: 358–372. 10.1007/s12010-009-8740-7. 19672721

[vms370321-bib-0034] Ashi, H. , E. Hamed , B. Refaat , et al. 2024. “Saffron (C*rocus sativus* Linnaeus) Based Protection Against Aflatoxin B1 Induced Organ Damage in Rats.” Acta Biologica Slovenica 67, no. 3: 4–20. 10.14720/abs.67.3.18966.

[vms370321-bib-0035] Ashktorab, H. , A. Soleimani , G. Singh , et al. 2019. “Saffron: The Golden Spice With Therapeutic Properties on Digestive Diseases.” Nutrients 11, no. 5: 943. 10.3390/nu11050943. 31027364 PMC6567082

[vms370321-bib-0036] Attard, G. , A. Bionda , F. Litrenta , et al. 2024. “Using Olive Cake as a Sustainable Ingredient in Diets of Lactating Dairy Cows: Effects on Nutritional Characteristics of Cheese.” Sustainability 16, no. 8: 3306. 10.3390/su16083306.

[vms370321-bib-0037] Attard, G. , L. Liotta , V. Lopreiato , V. Chiofalo , and A. R. Di Rosa . 2024. “Effects of Supplementing Pistachio Skins in the Diet on Growth Performance and the Fatty Acid Profile of *Biceps femoris* and *Longissimus dorsi* Muscles in Rabbits.” World Rabbit Science 32, no. 2: 99–108. 10.4995/wrs.2024.20230.

[vms370321-bib-0038] Azmat, F. , M. Safdar , H. Ahmad , et al. 2024. “Phytochemical Profile, Nutritional Composition of Pomegranate Peel and Peel Extract as a Potential Source of Nutraceutical: A Comprehensive Review.” Food Science & Nutrition 12, no. 2: 661–674. 10.1002/fsn3.3777. 38370077 PMC10867480

[vms370321-bib-0039] Badiee Baghsiyah, M. , M. Bashtani , and S. H. Farhangfar . 2023. “Effect of Grape By‐Products Inclusion on Ruminal Fermentation, Blood Metabolites, and Milk Fatty Acid Composition in Lactating Saanen Goats.” Iranian Journal of Applied Animal Science 13, no. 4: 731–742.

[vms370321-bib-0040] Bagheripour, E. , Y. Rouzbehan , and D. Alipour . 2008. “Effects of Ensiling, Air‐Drying and Addition of Polyethylene Glycol on *In Vitro* Gas Production of Pistachio By‐Products.” Animal Feed Science and Technology 146, no. 3–4: 327–336. 10.1016/j.anifeedsci.2008.01.002.

[vms370321-bib-0041] Bakeer, M. , H. Abdelrahman , and K. Khalil . 2021. “Effects of Pomegranate Peel and Olive Pomace Supplementation on Reproduction and Oxidative Status of Rabbit Doe.” Journal of Animal Physiology and Animal Nutrition 106, no. 3: 655–663. 10.1111/jpn.13617. 34318525

[vms370321-bib-0042] Bakr, M. H. , M. S. Farghaly , M. A. Hanafy , and M. A. Mahmoud . 2024. “Effect of Partial Replacement of TMR by Treated Olive Cake on Sheep Performance.” Egyptian Journal of Veterinary Sciences 56, no. 3: 437–448.

[vms370321-bib-0043] Barshan, H. , M. A. Azarbayjani , S. Rahmati , and M. Peeri . 2024. “The Effect of Aerobic Exercise and Pistachio Soft Hull Extract on the Expression of the IL‐6 and IL‐1β Genes in the Soleus Muscle of Female Rats Fed a High‐Fat Diet.” Gene, Cell Tissue 11, no. 2: e144065. 10.5812/gct-144065.

[vms370321-bib-0044] Belloumi, D. , P. García‐Rebollar , S. Calvet , et al. 2024. “Impact of Including Two Types of Destoned Olive Cakes in Pigs' Diets on Fecal Bacterial Composition and Study of the Relationship Between Fecal Microbiota, Feed Efficiency, Gut Fermentation, and Gaseous Emissions.” Frontiers in Microbiology 15: 1359670. 10.3389/fmicb.2024.1359670. 38946909 PMC11211982

[vms370321-bib-0045] Bennato, F. , A. Ianni , M. Florio , et al. 2022. “Nutritional Properties of Milk From Dairy Ewes Fed With a Diet Containing Grape Pomace.” Foods 11, no. 13: 1878. 10.3390/foods11131878. 35804692 PMC9265667

[vms370321-bib-0046] Beres, C. , G. N. S. Costa , I. Cabezudo , et al. 2017. “Towards Integral Utilization of Grape Pomace From Winemaking Process: A Review.” Waste Management 68: 581–594. 10.1016/j.wasman.2017.07.017. 28734610

[vms370321-bib-0047] Besharati, M. , and A. Taghizadeh . 2009. “Evaluation of Dried Grape By‐Product as a Tanniniferous Tropical Feedstuff.” Animal Feed Science and Technology 152, no. 3–4: 198–203. 10.1016/j.anifeedsci.2009.04.011.

[vms370321-bib-0048] Bešlo, D. , G. Došlić , D. Agić , et al. 2022. “Polyphenols in Ruminant Nutrition and Their Effects on Reproduction.” Antioxidants 11, no. 5: 970. 10.3390/antiox11050970. 35624834 PMC9137580

[vms370321-bib-0049] Bohluli, A. , A. Naserian , R. Valizadeh , and F. Eftekarshahroodi . 2007. “The Chemical Composition and *In Vitro* Digestibility of Pistachio By‐Product.” Proceedings of the British Society of Animal Science 2007: 223. 10.1017/s1752756200021268.

[vms370321-bib-0050] Bonos, E. , I. Skoufos , K. Petrotos , et al. 2022. “Innovative Use of Olive, Winery and Cheese Waste By‐Products as Functional Ingredients in Broiler Nutrition.” Veterinary Science 9, no. 6: 290. 10.3390/vetsci9060290. PMC9231388 35737342

[vms370321-bib-0051] Botsoglou, E. , P. Florou‐Paneri , E. Christaki , K. Fegeros , and I. Kafantaris . 2010. “Use of Saffron (*Crocus sativus* L.) as a Feed Additive for Improving Growth and Meat or Egg Quality in Poultry.” Functional Plant Biology 4, no. 2: 98–107.

[vms370321-bib-0052] Bouzaida, M. D. , V. C. Resconi , D. Gimeno , et al. 2021. “Effect of Dietary Grape Pomace on Fattening Rabbit Performance, Fatty Acid Composition, and Shelf Life of Meat.” Antioxidants 10, no. 5: 795. 10.3390/antiox10050795. 34067887 PMC8155864

[vms370321-bib-0053] Buffa, G. , E. Tsiplakou , C. Mitsiopoulou , G. Pulina , and A. Nudda . 2020. “Supplementation of By‐Products From Grape, Tomato and Myrtle Affects Antioxidant Status of Dairy Ewes and Milk Fatty Acid Profile.” Journal of Animal Physiology and Animal Nutrition 104, no. 2: 493–506. 10.1111/jpn.13315. 31989701

[vms370321-bib-0054] Çabuk, B. , M. G. Nosworthy , A. K. Stone , et al. 2018. “Effect of Fermentation on the Protein Digestibility and Levels of Non‐Nutritive Compounds of Pea Protein Concentrate.” Food Technology and Biotechnology 56, no. 2: 257–264. 10.17113/ftb.56.02.18.5450. 30228800 PMC6117996

[vms370321-bib-0055] Câmara, J. S. , S. Lourenço , C. Silva , A. Lopes , C. Andrade , and R. Perestrelo . 2020. “Exploring the Potential of Wine Industry By‐Products as Source of Additives to Improve the Quality of Aquafeed.” Microchemical Journal 155: 104758. 10.1016/j.microc.2020.104758.

[vms370321-bib-0056] Caponio, G. R. , F. Minervini , G. Tamma , G. Gambacorta , and M. De Angelis . 2023. “Promising Application of Grape Pomace and Its Agri‐Food Valorization: Source of Bioactive Molecules With Beneficial Effects.” Sustainability 15, no. 11: 9075. 10.3390/su15119075.

[vms370321-bib-0057] Castagna, F. , R. Bava , E. Palma , et al. 2024. “Effect of Pomegranate (*Punica granatum*) Anthelmintic Treatment on Milk Production in Dairy Sheep Naturally Infected With Gastrointestinal Nematodes.” Frontiers in Veterinary Science 11: 1347151. 10.3389/fvets.2024.1347151. 38384955 PMC10879392

[vms370321-bib-0058] Castellani, F. , A. Vitali , N. Bernardi , et al. 2017. “Dietary Supplementation With Dried Olive Pomace in Dairy Cows Modifies the Composition of Fatty Acids and the Aromatic Profile in Milk and Related Cheese.” Journal of Dairy Science 100, no. 11: 8658–8669. 10.3168/jds.2017-12899. 28843691

[vms370321-bib-0059] Cavallini, D. , L. M. E. Mammi , G. Biagi , et al. 2021. “Effects of 00‐Rapeseed Meal Inclusion in Parmigiano Reggiano Hay‐Based Ration on Dairy Cows' Production, Reticular pH and Fibre Digestibility.” Italian Journal of Animal Science 20, no. 1: 295–303. 10.1080/1828051X.2021.1884005.

[vms370321-bib-0060] Cavallucci, C. , B. Onelia , A. Nuccitelli , and D. Beghelli . 2024. “Olive Pomace By‐Products as Potential Functional Ingredient in Horse Nutrition.” In 29° SIVE (Società Italiana Veterinari per Equini) Congresso Internazionale: Atti Congressuali Proceeding, 602–603. EV (Editoria Scientifica).

[vms370321-bib-0061] Chaji, M. , and Z. Jahanara . 2023. “Use of Tannase‐Producing Bacteria Isolated From the Rumen to Improve the Nutritional Value of Pomegranate Peel for Fattening Lambs.” Veterinary Medicine and Science 10, no. 1: e31347. 10.1002/vms3.1347/v3/response1. PMC10790326 38227709

[vms370321-bib-0062] Chaves, B. W. , G. A. F. Valles , R. B. Scheibler , J. Schafhauser Junior , and J. L. Nornberg . 2020. “Milk Yield of Cows Submitted to Different Levels of Olive Pomace in the Diet.” Acta Scientiarum‐Animal Sciences 43: e51158. 10.4025/actascianimsci.v43i1.51158.

[vms370321-bib-0063] Cheng, X. , X. Du , Y. Liang , et al. 2023. “Effect of Grape Pomace Supplement on Growth Performance, Gastrointestinal Microbiota, and Methane Production in Tan Lambs.” Frontiers in Microbiology 14: 1264840. 10.3389/fmicb.2023.1264840. 37840727 PMC10569316

[vms370321-bib-0064] Chiofalo, V. , L. Liotta , V. Lo Presti , et al. 2020. “Effect of Dietary Olive Cake Supplementation on Performance, Carcass Characteristics, and Meat Quality of Beef Cattle.” Animals 10, no. 7: 1176. 10.3390/ani10071176. 32664412 PMC7401520

[vms370321-bib-0065] Cifuni, G. F. , S. Claps , G. Morone , et al. 2023. “Valorization of Olive Mill Byproducts: Recovery of Biophenol Compounds and Application in Animal Feed.” Plants 12, no. 17: 3062. 10.3390/plants12173062. 37687309 PMC10490477

[vms370321-bib-0066] Claro‐Cala, C. M. , J. C. Quintela , M. Pérez‐Montero , et al. 2020. “Pomace Olive Oil Concentrated in Triterpenic Acids Restores Vascular Function, Glucose Tolerance and Obesity Progression in Mice.” Nutrients 12, no. 2: 323. 10.3390/nu12020323. 31991894 PMC7071211

[vms370321-bib-0067] Correddu, F. , M. F. Caratzu , M. F. Lunesu , S. Carta , G. Pulina , and A. Nudda . 2023. “Grape, Pomegranate, Olive, and Tomato By‐Products Fed to Dairy Ruminants Improve Milk Fatty Acid Profile Without Depressing Milk Production.” Foods 12, no. 4: 865. 10.3390/foods12040865. 36832939 PMC9957115

[vms370321-bib-0068] Costa, M. M. , C. M. Alfaia , P. A. Lopes , J. M. Pestana , and J. A. M. Prates . 2022. “Grape By‐Products as Feedstuff for Pig and Poultry Production.” Animals 12, no. 17: 2239. 10.3390/ani12172239. 36077957 PMC9454619

[vms370321-bib-0069] Daler, S. 2024. “Improving Grapevine (*Vitis vinifera* L., cv. Superior Seedless) Drought Tolerance With Cerium Oxide Nanoparticles: Agronomic and Molecular Insights.” Scientia Horticulturae (Amsterdam) 338: 113606. 10.1016/j.scienta.2024.113606.

[vms370321-bib-0070] Daniel, T. , M. Ben‐Shachar , E. Drori , et al. 2021. “Grape Pomace Reduces the Severity of Non‐Alcoholic Hepatic Steatosis and the Development of Steatohepatitis by Improving Insulin Sensitivity and Reducing Ectopic Fat Deposition in Mice.” Journal of Nutritional Biochemistry 98: 108867. 10.1016/j.jnutbio.2021.108867. 34571189

[vms370321-bib-0071] Das, A. K. , P. K. Nanda , N. R. Chowdhury , et al. 2021. “Application of Pomegranate By‐Products in Muscle Foods: Oxidative Indices, Colour Stability, Shelf Life and Health Benefits.” Molecules (Basel, Switzerland) 26, no. 2: 467. 10.3390/molecules26020467. 33477314 PMC7830841

[vms370321-bib-0072] De Bellis, P. , A. Maggiolino , C. Albano , P. De Palo , and F. Blando . 2022. “Ensiling Grape Pomace With and Without Addition of a *Lactiplantibacillus plantarum* Strain: Effect on Polyphenols and Microbiological Characteristics, *In Vitro* Nutrient Apparent Digestibility, and Gas Emission.” Frontiers in Veterinary Science 9: 808293. 10.3389/fvets.2022.808293. 35280128 PMC8907520

[vms370321-bib-0073] Derbali, H. , S. Ben Saïd , K. Abid , et al. 2024. “Valorization of Dehydrated Grape Pomace Waste as a Low‐Cost Feed Additive to Improve Reproduction and Growth Performance of Male Rabbits.” Waste and Biomass Valorization 15, no. 7: 3987–3996. 10.1007/s12649-023-02410-2.

[vms370321-bib-0074] Difonzo, G. , M. A. Crescenzi , S. Piacente , G. Altamura , F. Caponio , and P. Montoro . 2022. “Metabolomics Approach to Characterize Green Olive Leaf Extracts Classified Based on Variety and Season.” Plants 11, no. 23: 3321. 10.3390/plants11233321. 36501360 PMC9735528

[vms370321-bib-0075] Duarte, L. , A. Bustamante , J. F. Orellana , et al. 2024. “Impact of Pomegranate Peel Extract on Gut Microbiota Composition and Metabolic Health Parameters in High‐Fat Diet‐Fed Mice.” Food Bioscience 61: 104663. 10.1016/j.fbio.2024.104663.

[vms370321-bib-0076] Ebadi, Z. , and A. Mahdavi . 2023. “The Nutritional Effect of Incorporating Different Percentages of Pistachio By‐Products Silages Into the Diet of Sheep on the Quantitative and Qualitative Characteristics of Meat.” Journal of Food Biosciences and Technology 13, no. 3: 53–62.

[vms370321-bib-0077] Ebrahimi, S. , M. H. F. Nasri , and S. H. Farhangfar . 2024. “Dietary Supplementation of Saffron Petal Elicits Positive Effects on Performance, Antioxidant Status, and Health of Dairy Goats.” Small Ruminant Research 231: 107179. 10.1016/j.smallrumres.2023.107179.

[vms370321-bib-0078] El‐Hadary, A. E. , and M. Taha . 2020. “Pomegranate Peel Methanolic‐Extract Improves the Shelf‐Life of Edible‐Oils Under Accelerated Oxidation Conditions.” Food Science & Nutrition 8, no. 4: 1798–1811. 10.1002/fsn3.1391. 32328245 PMC7174205

[vms370321-bib-0079] El‐Sabrout, K. , A. Sherasiya , S. Ahmad , et al. 2024. “Environmental Enrichment in Rabbit Husbandry: Comparative Impacts on Performance and Welfare.” Animals 14, no. 16: 2367. 10.3390/ani14162367. 39199901 PMC11350770

[vms370321-bib-0080] Eliopoulos, C. , G. Papadomichelakis , A. Voitova , et al. 2024. “Improved Antioxidant Blood Parameters in Piglets Fed Diets Containing Solid‐State Fermented Mixture of Olive Mill Stone Waste and Lathyrus Clymenum Husks.” Antioxidants 13, no. 6: 630. 10.3390/antiox13060630. 38929069 PMC11201101

[vms370321-bib-0081] Elsheikh, H. , E. Saddick , and U. Nayel . 2024. “Effect of Replacing Corn Grains With Citrus By‐Product in Concentrate Feed Blocks Form on the Performance and Feed Utilization of Lambs.” Journal of Animal and Poultry Production 15, no. 1: 1–8. 10.21608/jappmu.2024.259435.1100.

[vms370321-bib-0082] Emami, A. , M. H. Fathi Nasri , M. Ganjkhanlou , L. Rashidi , and A. Zali . 2016. “Effect of Pomegranate Seed Oil as a Source of Conjugated Linolenic Acid on Performance and Milk Fatty Acid Profile of Dairy Goats.” Livestock Science 193: 1–7. 10.1016/j.livsci.2016.09.004.

[vms370321-bib-0083] Erinle, T. J. , and D. I. Adewole . 2022. “Fruit Pomaces‐Their Nutrient and Bioactive Components, Effects on Growth and Health of Poultry Species, and Possible Optimization Techniques.” Animal Nutrition 9: 357–377. 10.1016/j.aninu.2021.11.011. 35600557 PMC9110891

[vms370321-bib-0084] Erinle, T. J. , S. Oladokun , J. MacIsaac , B. Rathgeber , and D. Adewole . 2022. “Dietary Grape Pomace—Effects on Growth Performance, Intestinal Health, Blood Parameters, and Breast Muscle Myopathies of Broiler Chickens.” Poultry Science 101, no. 1: 101519. 10.1016/j.psj.2021.101519. PMC8605297 34794081

[vms370321-bib-0085] Esmaili, N. , O. Dayani , R. Tahmasbi , M. M. Sharifi Hoseini , and Z. Hajalizadeh . 2021. “Effect of Pistachio Seed Coat on Feed Intake and Nitrogen Retention in Kermani Sheep.” Animal Production 23, no. 3: 351–362.

[vms370321-bib-0086] Espeso, J. , A. Isaza , J. Y. Lee , et al. 2021. “Olive Leaf Waste Management.” Frontiers in Sustainable Food Systems 5: 1–13. 10.3389/fsufs.2021.660582.

[vms370321-bib-0087] de Evan, T. , C. N. Marcos , and M. D. Carro . 2024. “Chemical Composition and *In Vitro* Nutritive Evaluation of Pomegranate and Artichoke Fractions as Ruminant Feed.” Ruminants 4, no. 1: 1–9. 10.3390/ruminants4010001.

[vms370321-bib-0088] Fallahi, H. R. , and S. Mahmoodi . 2018. “Influence of Organic and Chemical Fertilization on Saffron Growth.” Saffron Agronomy and Technology 6, no. 2: 147–166.

[vms370321-bib-0089] Fang, L. , M. Li , L. Zhao , et al. 2020. “Dietary Grape Seed Procyanidins Suppressed Weaning Stress by Improving Antioxidant Enzyme Activity and mRNA Expression in Weanling Piglets.” Journal of Animal Physiology and Animal Nutrition 104, no. 4: 1178–1185. 10.1111/jpn.13335. 32189416

[vms370321-bib-0090] FAO/IAEA . 2000. Quantification of Tannins in Tree Foliage . Joint FAO/IAEA Division of Nuclear Techniques in Food and Agriculture.

[vms370321-bib-0091] Faryabidoust, B. , L. Asadpour , and S. Nassabian . 2013. “The Study of the Effect of Multi‐Enzyme Supplementation and Different Levels of Tomato Pomace on the Performance and Carcass Characteristics of Broiler Chicks.” Global Veterinary 10, no. 3: 337–342.

[vms370321-bib-0092] Fatehi, P. , A. A. Alamouti , M. Behgar , and M. A. Norouzian . 2020. “Effect of Electron Irradiation on Some Physical, Chemical and Digestion Properties of Pistachio By‐Products.” Radiation Physics and Chemistry 174: 108921. 10.1016/j.radphyschem.2020.108921.

[vms370321-bib-0093] Fayed, A. 2019. “Influence of Feeding Mixture of Tomato and Apple Pomace Silage to Lactating Goats on Productive Performance.” Egyptian Journal of Sheep and Goats Sciences 11, no. 3: 1–13.

[vms370321-bib-0094] Flores, D. R. M. , P. A. F. da Fonseca , J. Schmitt , et al. 2020. “Lambs Fed With Increasing Levels of Grape Pomace Silage: Effects on Productive Performance, Carcass Characteristics, and Blood Parameters.” Livestock Science 240: 104169. 10.1016/j.livsci.2020.104169.

[vms370321-bib-0095] Frank, J. , N. K. Fukagawa , A. R. Bilia , et al. 2020. “Terms and Nomenclature Used for Plant‐Derived Components in Nutrition and Related Research: Efforts Toward Harmonization.” Nutrition Reviews 78, no. 6: 451–458. 10.1093/nutrit/nuz081. 31769838 PMC7212822

[vms370321-bib-0096] Friedman, M. 2014. “Antibacterial, Antiviral, and Antifungal Properties of Wines and Winery Byproducts in Relation to Their Flavonoid Content.” Journal of Agricultural and Food Chemistry 62, no. 26: 6025–6042. 10.1021/jf501266s. 24945318

[vms370321-bib-0097] Gasparini, M. , G. Brambilla , S. Menotta , et al. 2024. “Sustainable Dairy Farming and Fipronil Risk in Circular Feeds: Insights From an Italian Case Study.” Food Additives and Contaminants: Part A 41, no. 12: 1582–1593. 10.1080/19440049.2024.2414954. 39446071

[vms370321-bib-0098] Ghoreishi, S. M. , A. R. Zare , M. R. Rezvani , M. J. Zamiri , S. Kargar , and M. J. Abarghuei . 2021. “Partial Replacement of Forage and Concentrate With Pomegranate Pulp (Peel and Seed) Silage and Pomegranate Seed Pulp in Mehraban Fattening Lambs: Effect on Performance and Carcass Characteristics.” Tropical Animal Health and Production 53, no. 5: 486. 10.1007/s11250-021-02901-1. 34586503

[vms370321-bib-0099] Giannuzzi, D. , A. Toscano , S. Pegolo , et al. 2022. “Associations Between Milk Fatty Acid Profile and Body Condition Score.” Ultrasound Hepatic Measurements and Blood Metabolites in Holstein Cows. Animals 12, no. 9: 1202. 10.3390/ani12091202. 35565628 PMC9104722

[vms370321-bib-0100] Giuliani, C. D. S. , A. G. R. Júnior , A. Mateus , et al. 2024. “Meat Quality of Pigs Fed Grape Pomace in Different Production Systems.” Anais Da Academia Brasileira De Ciencias 96, no. 1: e20220610. 10.1590/0001-3765202320220610. 38451592

[vms370321-bib-0101] Goli, S. A. H. , F. Mokhtari , and M. Rahimmalek . 2012. “Phenolic Compounds and Antioxidant Activity From Saffron (*Crocus sativus* L.) Petal.” Journal of Agricultural Science 4, no. 10: 175–181. 10.5539/jas.v4n10p175.

[vms370321-bib-0102] Gonçalves, T. R. , J. R. V. da Silva Filho , G. A. Pereira , et al. 2024. “Potential Use of Vitiviniculture Waste in Mixed Cactus Pear Silages With Elephant Grass in Lamb Diet.” Animal Feed Science and Technology 307: 115824. 10.1016/j.anifeedsci.2023.115824.

[vms370321-bib-0103] Govoni, C. , P. D'Odorico , L. Pinotti , and M. C. Rulli . 2023. “Preserving Global Land and Water Resources Through the Replacement of Livestock Feed Crops With Agricultural By‐Products.” Nature Food 4, no. 12: 1047–1057. 10.1038/s43016-023-00884-w. 38053006

[vms370321-bib-0104] Grgas, D. , M. Rukavina , D. Bešlo , et al. 2023. “The Bacterial Degradation of Lignin—A Review.” Water 15, no. 7: 1272. 10.3390/w15071272.

[vms370321-bib-0105] Grioui, N. , I. B. Slimen , H. Riahi , T. Najar , M. Abderrabba , and M. Mejri . 2019. “Influence of Dried Tomato Pomace as a Source of Polyphenols on the Performance of Growing Rabbit.” Animal Nutrition and Feed Technology 19, no. 3: 493. 10.5958/0974-181x.2019.00045.3.

[vms370321-bib-0106] Grosell, M. , and J. Genz . 2006. “Ouabain‐Sensitive Bicarbonate Secretion and Acid Absorption by the Marine Teleost Fish Intestine Play a Role in Osmoregulation.” American Journal of Physiology – Regulatory, Integrative and Comparative Physiology 291, no. 4: R1145–R1156. 10.1152/ajpregu.00818.2005. 16709644

[vms370321-bib-0107] Guerra‐Rivas, C. , B. Gallardo , Á. R. Mantecón , M. del Álamo‐Sanza , and T. Manso . 2016. “Evaluation of Grape Pomace From Red Wine By‐Product as Feed for Sheep.” Journal of the Science of Food and Agriculture 97, no. 6: 1885–1893. 10.1002/jsfa.7991. 27508943

[vms370321-bib-0108] Gullón, P. , G. Astray , B. Gullón , I. Tomasevic , and J. M. Lorenzo . 2020. “Pomegranate Peel as Suitable Source of High‐Added Value Bioactives: Tailored Functionalized Meat Products.” Molecules (Basel, Switzerland) 25, no. 12: 2859. 10.3390/molecules25122859. 32575814 PMC7355679

[vms370321-bib-0109] Gungor, E. , A. Altop , and G. Erener . 2024. “Effect of Fermented Tomato Pomace on the Growth Performance, Antioxidant Capacity, and Intestinal Microflora in Broiler Chickens.” Animal Science Journal 95, no. 1: e13885. 10.1111/asj.13885. 38221671

[vms370321-bib-0110] Habib, H. G. , I. F. Al‐Zamili , and J. K. Al‐Gharawi . 2023. “Effect of Adding Different Levels of Dried Olive Pomace to the Diet on Some Blood Traits of Broilers.” IOP Conference Series: Earth and Environmental Science 1225, no. 1: 12038. 10.1088/1755-1315/1225/1/012038.

[vms370321-bib-0111] Hafeez, A. , Q. Piral , S. Naz , et al. 2023. “Ameliorative Effect of Pomegranate Peel Powder on the Growth Indices, Oocysts Shedding, and Intestinal Health of Broilers Under an Experimentally Induced Coccidiosis Condition.” Animals 13, no. 24: 3790. 10.3390/ani13243790. 38136827 PMC10740919

[vms370321-bib-0112] Hafez, A. A. 2024. “Impact of Tomato Pomace, Enzymes and/or Amino Acids on Blood Parameters, Serum Biochemicals, Antioxidants, Immune Status and Expression of Immune‐Related Genes in *Nile tilapia* Fish (*Oreochromis nilotius*).” Egyptian Journal of Veterinary Sciences 1–11. 10.21608/ejvs.2024.290519.2096.

[vms370321-bib-0113] Hafez, A. A. , E. El‐Nassef , A. A. Bakr , E. M. Moustafa , W. S. Abdo , and E. M. Hegazi . 2024. “Tomato Pomace With Exogenous Enzymes and/or Amino Acids Enhanced Growth Performance, Histological Parameters and Gene Expression for Growth in *Nile tilapia* Fish (*Oreochromis nilotius*).” Egyptian Journal of Veterinary Sciences 1–11. 10.21608/ejvs.2024.284053.2023.

[vms370321-bib-0114] Hagag, O. Y. A.‐E. , F. E.‐E. Younis , R. A. Al‐Eisa , E. Fayad , and N. S. El‐Shenawy . 2023. “Effect of Feeding Pomegranate (*Punica granatum*) Peel and Garlic (*Allium sativum*) on Antioxidant Status and Reproductive Efficiency of Female Rabbits.” Veterinary Science 10, no. 3: 179. 10.3390/vetsci10030179. PMC10051658 36977218

[vms370321-bib-0115] Hagerman, A. , and L. G. Butler . 1991. “Tannins and Lignins.” In Herbivores: Their Interactions With Secondary Plant Metabolites, 355–388. Academic Press. 10.1016/b978-0-12-597183-6.50015-2.

[vms370321-bib-0116] Hajalizadeh, Z. , and O. Dayani . 2021. “Ruminal Fermentation, Microbial Protein Synthesis and Nitrogen Balance in Sheep Fed Pistachio By‐Product Silage.” Journal of Animal Science and Technology 9, no. 1: 23–30.

[vms370321-bib-0004] Hamady, G. A. A. , M. A. Abdel‐Moneim , G. A. El‐Chaghaby , Z. M. Abd‐El‐Ghany , and M. S. Hassanin . 2015. “Effect of Pomegranate Peel Extract as Natural Growth Promoter on the Productive Performance and Intestinal Microbiota of Broiler Chickens.” African Journal of Agricultural Science and Technology 3, no. 12: 514–519. http://www.oceanicjournals.org/ajast.

[vms370321-bib-0117] Hamed, M. , A. Feriani , A. Sila , J. Sdayria , A. Haddar , and A. Bougatef . 2021. “Sustainable Valorization of Pistachio (*Pistacia vera* L.) by Product Through Recovering Protective Polysaccharides Against Hepatotoxicity and Nephrotoxicity in Rats.” Waste and Biomass Valorization 13, no. 1: 467–479. 10.1007/s12649-021-01545-4.

[vms370321-bib-0118] Hassan, F. A. , M. R. M. Ibrahim , and S. A. Arafa . 2020. “Effect of Dietary Pomegranate By‐Product Extract Supplementation on Growth Performance, Digestibility, and Antioxidant Status of Growing Rabbit.” Tropical Animal Health and Production 52, no. 4: 1893–1901. 10.1007/s11250-020-02201-0. 31955376

[vms370321-bib-0119] Hassan, F. A. , M. S. Mohamed , D. O. Othman , et al. 2024. “Growth Performance, Plasma Metabolites, Meat Quality, and Meat and Lipid Health Indices of New Zealand White Rabbits as Affected by Dietary Dried Tomato Pomace Powder Supplementation During the Summer Season.” Journal of Animal Physiology and Animal Nutrition 108, no. 4: 1083–1095. 10.1111/jpn.13953. 38528432

[vms370321-bib-0120] He, Y. , Z. Li , F. Tan , et al. 2019. “Fatty Acid Metabolic Flux and Lipid Peroxidation Homeostasis Maintain the Biomembrane Stability to Improve Citrus Fruit Storage Performance.” Food Chemistry 292: 314–324. 10.1016/j.foodchem.2019.04.009. 31054680

[vms370321-bib-0121] Heidari Safar, Z. , A. Sadeghi , and A. Karimi . 2024. “Using Tomato, Apple, and Carrot Pomaces as a Non‐Fasting Method for Molt Induction in Laying Hens.” Animal Science Journal 36, no. 141: 117–132.

[vms370321-bib-0122] Hemmati Kakhki, A. 2001. “Optimization of Factors Affecting Production of Edible Colors From Saffron Petals.” Journal of Agricultural Science and Technology 15, no. 2: 13–20.

[vms370321-bib-0123] Herremans, S. , V. Decruyenaere , Y. Beckers , and E. Froidmont . 2018. “Silage Additives to Reduce Protein Degradation During Ensiling and Evaluation of *In Vitro* Ruminal Nitrogen Degradability.” Grass and Forage Science 74, no. 1: 86–96. 10.1111/gfs.12396.

[vms370321-bib-0124] Hosseini Ghaffari, M. , A. M. Tahmasbi , M. Khorvash , A. A. Naserian , A. Hosseini Ghaffari , and H. Valizadeh . 2013a. “Effects of Pistachio By‐Products in Replacement of Alfalfa Hay on Populations of Rumen Bacteria Involved in Biohydrogenation and Fermentative Parameters in the Rumen of Sheep.” Journal of Animal Physiology and Animal Nutrition 98, no. 3: 578–586. 10.1111/jpn.12120. 23957535

[vms370321-bib-0125] Hosseini Ghaffari, M. , A. M. Tahmasbi , M. Khorvash , A. A. Naserian , and A. R. Vakili . 2013b. “Effects of Pistachio By‐Products in Replacement of Alfalfa Hay on Ruminal Fermentation, Blood Metabolites, and Milk Fatty Acid Composition in Saanen Dairy Goats Fed a Diet Containing Fish Oil.” Journal of Applied Animal Research 42, no. 2: 186–193. 10.1080/09712119.2013.824889.

[vms370321-bib-0126] Ikusika, O. O. , O. F. Akinmoladun , and C. T. Mpendulo . 2024. “Enhancement of the Nutritional Composition and Antioxidant Activities of Fruit Pomaces and Agro‐Industrial Byproducts Through Solid‐State Fermentation for Livestock Nutrition: A Review.” Fermentation 10, no. 5: 227. 10.3390/fermentation10050227.

[vms370321-bib-0127] Inzunza‐Soto, M. , S. Thai , A. J. G. Sinrod , et al. 2021. “Health Benefits of First and Second Extraction Drum‐Dried Pitted Olive Pomace.” Journal of Food Science 86, no. 11: 4865–4876. 10.1111/1750-3841.15925. 34642970

[vms370321-bib-0128] Jadouali, S. M. , H. Atifi , Z. Bouzoubaa , et al. 2018. “Chemical Characterization, Antioxidant and Antibacterial Activity of Moroccan *Crocus sativus* L. Petals and Leaves.” Journal of Materials and Environmental Science 9, no. 1: 113–118. 10.26872/jmes.2018.9.1.14.

[vms370321-bib-0129] Jadouali, S. M. , H. Atifi , R. Mamouni , K. Majourhat , Z. Bouzoubaa , and S. Gharby . 2021. “Composition of Saffron By‐Products (*Crocus sativus*) in Relation to Utilization as Animal Feed.” Agricultural Science Digest – A Research Journal 42: 475–481. 10.18805/ag.d-360.

[vms370321-bib-0130] Jalal, H. , S. C. Doğan , M. Giammarco , et al. 2024. “Evaluation of Dietary Supplementation of Garlic Powder (*Allium sativum*) on the Growth Performance, Carcass Traits and Meat Quality of Japanese Quails (*Coturnix coturnix japonica*).” Poultry Science 103, no. 12: 104231. 10.1016/j.psj.2024.104231. PMC11415670 39255542

[vms370321-bib-0131] Javanmard Dakheli, M. 2020. “Effects of Grape and Pomegranate Waste Extracts on Poultry Carcasses Microbial, Chemical, and Sensory Attributes in Slaughterhouse.” Food Science & Nutrition 8, no. 10: 5622–5630. 10.1002/fsn3.1840. 33133564 PMC7590302

[vms370321-bib-0132] Jin, Y. , P. Wang , F. Li , et al. 2024. “The Effects of *Lactobacillus plantarum* and *Lactobacillus buchneri* on the Fermentation Quality, *In Vitro* Digestibility, and Aerobic Stability of *Silphium perfoliatum* L. Silage.” Animals 14, no. 15: 2279. 10.3390/ani14152279. 39123805 PMC11310989

[vms370321-bib-0133] Kahraman, M. , S. Yurtseven , E. Sakar , et al. 2023. “Pistachio, Pomegranate and Olive Byproducts Added to Sheep Rations Change the Biofunctional Properties of Milk Through the Milk Amino Acid Profile.” Food Science of Animal Resources 43, no. 1: 124–138. 10.5851/kosfa.2022.e65. 36789194 PMC9890361

[vms370321-bib-0134] Kamel, E. R. , B. M. Shafik , M. Mamdouh , S. Elrafaay , and F. A. I. Abdelfattah . 2021. “Response of Two Strains of Growing Japanese Quail (*Coturnix coturnix japonica*) to Diet Containing Pomegranate Peel Powder.” Tropical Animal Health and Production 53, no. 6: 549. 10.1007/s11250-021-02987-7. 34782923

[vms370321-bib-0135] Kandylis, P. , and E. Kokkinomagoulos . 2020. “Food Applications and Potential Health Benefits of Pomegranate and Its Derivatives.” Foods 9, no. 2: 122. 10.3390/foods9020122. 31979390 PMC7074153

[vms370321-bib-0136] Karampour, A. R. , R. N. Harsini , and F. Kafilzadeh . 2024. “Physicochemical Characteristics and Fatty Acid Profile of Meat and Adipose Tissue From Lambs Fed Diets With Different Levels of Pomegranate Seed Oil.” Iranian Journal of Applied Animal Science 14, no. 1: 67–79.

[vms370321-bib-0137] Kardan Moghadam, V. , M. H. Fathi Nasri , R. Valizadeh , and H. Farhangfar . 2014. “Growth Nutritive Value of Saffron Residues Harvested at Different Stages.” Iranian Journal of Animal Science Research 6, no. 1: 32–44.

[vms370321-bib-0138] Kardan Moghaddam, V. , M. A. Fathi Nasri Behdani , H. Kardan Moghaddam , and M. H. Fathi Nasari . 2015. “Effect of Pleurotus Florida Fungi on Chemical Composition, Ruminal Degradability and Gas Production of Saffron Foliage Residues.” Journal of Saffron Research 3, no. 2: 175–187.

[vms370321-bib-0139] Kaveh, H. , and A. Salari . 2018. “Study and Comparison of Saffron Quality in Khorasan Provinces.” Saffron Agronomy and Technology 6, no. 2: 209–218.

[vms370321-bib-0140] Kazemi, M. , H. Saleh , and B. Fahmideh . 2020. “Nutritional Value and Ensiling Ability of Saffron Wastes.” Saffron Agronomy and Technology 8, no. 4: 558–574.

[vms370321-bib-0141] Kazemi, M. 2024. “Determination of the Nutritional Potential and Valorization of Saffron (*Crocus sativus* L.) Wastes as an Antioxidant Agent in Ruminant Feeding: *In Vitro* and *In Vivo* Studies.” Tropical Animal Health and Production 56, no. 8: 350. 10.1007/s11250-024-04206-5. 39441224

[vms370321-bib-0142] Kazemi, M. , and R. Valizadeh . 2021. “The Effect of Dietary Supplementation of Ensiled Pomegranate By‐Products on Growth Performance, Nutrient Digestibility, Haematology Parameters and Meat Characteristics of Fat‐Tail Lambs.” Italian Journal of Animal Science 20, no. 1: 1532–1543. 10.1080/1828051x.2021.1986429.

[vms370321-bib-0143] Kazemi, M. , R. Valizadeh , and E. Ibrahimi Khoram Abadi . 2022. “Yogurt and Molasses Can Alter Microbial‐Digestive and Nutritional Characteristics of Pomegranate Leaves Silage.” AMB Express 12, no. 1: 111. 10.1186/s13568-022-01452-4. 36048307 PMC9437189

[vms370321-bib-0144] Kazemi, M. , R. Valizadeh , and A. Z. M. Salem . 2024. “Dietary Inclusion of Pistachio Wastes (*Pistacia vera* L.) to Fattening Male Goat Kids' Feeding: Chemical‐Mineral Compositions, *In Vitro* Ruminal Fermentation, In Vivo Digestibility, Hemato‐Biochemical Profile, and Growth Performance.” Small Ruminant Research 235: 107274. 10.1016/j.smallrumres.2024.107274.

[vms370321-bib-0145] Kemboi, F. , J. O. Ondiek , A. M. King'ori , and P. A. Onjoro . 2023. “Effects of Polyethylene Glycol (PEG 6000) and Bentonite Clay Incorporation in Selected Local Browse‐Based Diets on the Performance of Small East African Goats.” Tropical Animal Health and Production 55, no. 2: 124. 10.1007/s11250-023-03545-z. 36943532

[vms370321-bib-0146] Kesbiç, O. S. , Ü. Acar , M. S. Hassaan , S. Yılmaz , M. C. Guerrera , and F. Fazio . 2022. “Effects of Tomato Paste By‐Product Extract on Growth Performance and Blood Parameters in Common Carp (*Cyprinus carpio*).” Animals 12, no. 23: 3387. 10.3390/ani12233387. 36496908 PMC9737255

[vms370321-bib-0147] Khejornsart, P. , T. Juntanam , P. Gunun , N. Gunun , and A. Cherdthong . 2024. “Effect of High‐Tannin and Polyphenol Plant Material Supplement on Rumen Fermentation, Nitrogen Partitioning, and Nutrient Utilization in Beef Cattle.” Animals 14, no. 21: 3092. 10.3390/ani14213092. 39518815 PMC11545557

[vms370321-bib-0148] Khiaosa‐Ard, R. , M. Mahmood , E. Mickdam , C. Pacífico , J. Meixner , and L.‐S. Traintinger . 2023. “Winery By‐Products as a Feed Source With Functional Properties: Dose‐Response Effect of Grape Pomace, Grape Seed Meal, and Grape Seed Extract on Rumen Microbial Community and Their Fermentation Activity in RUSITEC.” Journal of Animal Science and Biotechnology 14, no. 1: 92. 10.1186/s40104-023-00892-7. 37424021 PMC10332069

[vms370321-bib-0149] Khorsandi, S. , A. Riasi , M. Khorvash , and F. Hashemzadeh . 2019. “Nutrients Digestibility, Metabolic Parameters and Milk Production in Postpartum Holstein Cows Fed Pomegranate (*Punica granatum* L.) By‐Products Silage Under Heat Stress Condition.” Animal Feed Science and Technology 255: 114213. 10.1016/j.anifeedsci.2019.114213.

[vms370321-bib-0150] Khoshbakht Fahim, N. , S. S. Fakoor Janati , and J. Feizy . 2012. “Chemical Composition of Saffron (*Crocus sativus* L.) Petals as Animal Feed.” GIDA 37, no. 4: 197–201.

[vms370321-bib-0151] Khoshvaght, A. , S. Zeinoaldini , and M. Ganjkhanlou . 2024. “The Effect of Adding Green Zinc Oxide Nanoparticles to Ram Semen Dilution Medium and Its Effects on Sperm Quality and Microbial Load of Frozen Semen.” Iranian Journal of Animal Science Research 16, no. 1: 141–155.

[vms370321-bib-0152] Kim, Y. , S. A. Lee , and H. H. Stein . 2024. “Determination of Energy Values in Pistachio Shell Powder and Soybean Hulls Fed to Gestating and Lactating Sows.” Translational Animal Science 8: txae135. 10.1093/tas/txae135. 39387097 PMC11462085

[vms370321-bib-0153] Kiralan, M. , and O. Ketenoglu . 2022. “Utilization of Tomato (*Solanum lycopersicum*) By‐Products: An Overview.” In Mediterr Fruits Bio‐Wastes, 799–818. Springer. 10.1007/978-3-030-84436-3_34.

[vms370321-bib-0154] Knez, E. , K. Kadac‐Czapska , and M. Grembecka . 2023. “Effect of Fermentation on the Nutritional Quality of the Selected Vegetables and Legumes and Their Health Effects.” Life 13, no. 3: 655. 10.3390/life13030655. 36983811 PMC10051273

[vms370321-bib-0155] Koakoski, D. L. , T. Bordin , D. Cavallini , and G. Buonaiuto . 2024. “A Preliminary Study of the Effects of Gaseous Ozone on the Microbiological and Chemical Characteristics of Whole‐Plant Corn Silage.” Fermentation 10, no. 8: 398. 10.3390/fermentation10080398.

[vms370321-bib-0156] Kolláthová, R. 2021. “Grape Pomace in Equine Nutrition: Effect on Antioxidant Status.” Acta fytotechnica et zootechnica 24, no. 4: 340–344. 10.15414/afz.2021.24.04.340-344.

[vms370321-bib-0157] Kolláthová, R. , B. Gálik , M. Halo , et al. 2020. “The Effects of Dried Grape Pomace Supplementation on Biochemical Blood Serum Indicators and Digestibility of Nutrients in Horses.” Czech Journal of Animal Science 65, no. 2: 58–65. 10.17221/181/2019-cjas.

[vms370321-bib-0158] Kordi, M. , and A. Naserian . 2012. “Influence of Wheat Bran as a Silage Additive on Chemical Composition and *In Vitro* Gas Production of Citrus Pulp Silage.” African Journal of Biotechnology 11, no. 63: 12669–12674.

[vms370321-bib-0159] Kordi, M. , A. A. Naserian , and F. Samadian . 2022. “The Influences of Adding Polyethylene Glycol and Activated Sodium Bentonite on the Performance, Blood Parameters, and Muscle Mineral Content of Saanen Goats Fed Pistachio By‐Products.” Iranian Journal of Applied Animal Science 12, no. 2: 303–312.

[vms370321-bib-0160] Kotsampasi, B. , C. Christodoulou , A. Mavrommatis , et al. 2021. “Effects of Dietary Pomegranate Seed Cake Supplementation on Performance, Carcass Characteristics and Meat Quality of Growing Lambs.” Animal Feed Science and Technology 273: 114815. 10.1016/j.anifeedsci.2021.114815.

[vms370321-bib-0161] Lamtar Mohammadi, A. , M. Torki , and M. Mottaghitalab . 2021. “Effect of Adding Garlic and Tomato Pomace Powder to Diet on the Body Weight, Productive Performance, and Egg Quality Traits in Broiler Breeders.” Animal Production Research 10, no. 3: 79–87.

[vms370321-bib-0162] Laranjeira, T. , A. Costa , C. Faria‐Silva , et al. 2022. “Sustainable Valorization of Tomato By‐Products to Obtain Bioactive Compounds: Their Potential in Inflammation and Cancer Management.” Molecules (Basel, Switzerland) 27, no. 5: 1701. 10.3390/molecules27051701. 35268802 PMC8911995

[vms370321-bib-0163] Leite, A. , L. Vasconcelos , S. Lopez , et al. 2024. “Incorporating Olive By‐Products in Bísaro Pig Diets: Effect on Dry‐Cured Product Quality.” Foods 13, no. 16: 2579. 10.3390/foods13162579. 39200506 PMC11353563

[vms370321-bib-0164] Li, Y. , C. Shi , J. Deng , et al. 2024. “Effects of Grape Pomace on Growth Performance, Nitrogen Metabolism, Antioxidants, and Microbial Diversity in Angus Bulls.” Antioxidants 13, no. 4: 412. 10.3390/antiox13040412. 38671860 PMC11047470

[vms370321-bib-0165] Lima Júnior, D. M. D. , P. Monteiro , A. Rangel , M. D. V. Maciel , S. E. O. Oliveira , and D. Freire . 2010. “Antinutritional Factors for Ruminants.” Acta Veterinaria Brasilica 4, no. 3: 132–143.

[vms370321-bib-0166] Lioliopoulou, S. , G. A. Papadopoulos , I. Giannenas , et al. 2023. “Effects of Dietary Supplementation of Pomegranate Peel With Xylanase on Egg Quality and Antioxidant Parameters in Laying Hens.” Antioxidants 12, no. 1: 208. 10.3390/antiox12010208. 36671069 PMC9854943

[vms370321-bib-0167] Lotfi, H. , B. Navidshad , M. Assadi , et al. 2021. “Evaluation of Multi‐Enzyme Effect on Performance and Intestinal Morphological of Broiler Chickens Fed by Tomato Pomace.” Journal of Plasma Biomarkers 14, no. 4: 1–16.

[vms370321-bib-0168] Lu, S. , S. Chen , H. Li , et al. 2022. “Sustainable Valorization of Tomato Pomace (*Lycopersicon esculentum*) in Animal Nutrition: A Review.” Animals 12, no. 23: 3294. 10.3390/ani12233294. 36496814 PMC9736048

[vms370321-bib-0169] Ma, J. , X. Fan , W. Zhang , et al. 2023. “Grape Seed Extract as a Feed Additive Improves the Growth Performance, Ruminal Fermentation and Immunity of Weaned Beef Calves.” Animals 13, no. 11: 1876. 10.3390/ani13111876. 37889835 PMC10251878

[vms370321-bib-0170] Mahood, H. E. , A. A. Dahham , V. Sarropoulou , and T.‐T. Tzatzani . 2023. “Extraction of Phenolic and Flavonoid Compounds and Evaluation of Their Antioxidant Activity in Saffron Anthers (*Crocus sativus* L.).” Notulae Scientia Biologicae 15, no. 4: 11640. 10.55779/nsb15411640.

[vms370321-bib-0171] Majoul, T. , K. Charradi , F. Limam , and E. Aouani . 2023. “Grape Processing Waste: A Safe Extract to Alleviate High‐Fat‐Diet‐Induced Lipotoxicity and Energy Fueling Alteration in Rat Heart.” Waste and Biomass Valorization 15, no. 4: 2249–2257. 10.1007/s12649-023-02290-6.

[vms370321-bib-0172] Makkar, H. P. S. , M. Blümmel , and K. Becker . 1995. “Formation of Complexes Between Polyvinyl Pyrrolidones or Polyethylene Glycols and Tannins, and Their Implication in Gas Production and True Digestibility in *In Vitro* Techniques.” British Journal of Nutrition 73, no. 6: 897–913. 10.1079/bjn19950095. 7632671

[vms370321-bib-0173] Malek, H. , P. Farhoomand , R. Farah , and M. Daneshyar . 2021. “Effect of Different Levels of Dietary Tomato Pomace on Performance, Chick Quality Parameters and Meat Malondialdehyde Content of Progeny in Quail Breeders.” Journal of Animal Science and Research 30, no. 4: 83–94.

[vms370321-bib-0174] Manoni, M. , F. Gschwend , S. Amelchanka , et al. 2024. “Gallic and Ellagic Acids Differentially Affect Microbial Community Structures and Methane Emission When Using a Rumen Simulation Technique.” Journal of Agricultural and Food Chemistry 72, no. 49: 27163–27176. 10.1021/acs.jafc.4c06214. 39588639 PMC11638960

[vms370321-bib-0175] Manoni, M. , M. Terranova , S. Amelchanka , L. Pinotti , P. Silacci , and M. Tretola . 2023. “Effect of Ellagic and Gallic Acid on the Mitigation of Methane Production and Ammonia Formation in an *In Vitro* Model of Short‐Term Rumen Fermentation.” Animal Feed Science and Technology 305: 115791. 10.1016/j.anifeedsci.2023.115791.

[vms370321-bib-0176] Maqsood, S. , S. Naz , A. Sikandar , S. Arooj , A. Fahad Alrefaei , and M. Israr . 2024. “Additive Effect of *Moringa oleifera* Leaf Meal and Pomegranate (*Punica granatum*) Peel Powder on Productive Performance, Carcass Attributes and Histological Morphology of Ileum in Japanese Quails.” Journal of Applied Animal Research 52, no. 1: 1–7. 10.1080/09712119.2024.2319269.

[vms370321-bib-0177] Marcos, C. N. , T. de Evan , E. Molina‐Alcaide , and M. D. Carro . 2019. “Nutritive Value of Tomato Pomace for Ruminants and Its Influence on *In Vitro* Methane Production.” Animals 9, no. 6: 343. 10.3390/ani9060343. 31212765 PMC6616965

[vms370321-bib-0178] Marrone, G. , S. Urciuoli , M. Di Lauro , et al. 2024. “Saffron (*Crocus sativus* L.) and Its By‐Products: Healthy Effects in Internal Medicine.” Nutrients 16, no. 14: 2319. 10.3390/nu16142319. 39064764 PMC11279474

[vms370321-bib-0179] Masmoudi, R. , N. Ben Yahmed , N. Moujahed , C. Darej , and I. Smaali . 2024. “Detoxification and Enhancement of *In Vitro* Rumen Digestibility of Exhausted Olive Pomace Wastes Through Alkaline Hydrogen Peroxide Treatment.” Chemical and Biological Technologies in Agriculture 11, no. 1: 16. 10.1186/s40538-024-00533-9.

[vms370321-bib-0180] Mazza, P. H. S. , S. Jaeger , F. L. Silva , et al. 2020. “Effect of Dehydrated Residue From Acerola (*Malpighia emarginata* DC.) Fruit Pulp in Lamb Diet on Intake, Ingestive Behavior, Digestibility, Ruminal Parameters and N Balance.” Livestock Science 233: 103938. 10.1016/j.livsci.2020.103938.

[vms370321-bib-0181] McSweeney, C. S. , B. Palmer , D. M. McNeill , and D. O. Krause . 2001. “Microbial Interactions With Tannins: Nutritional Consequences for Ruminants.” Animal Feed Science and Technology 91, no. 1–2: 83–93. 10.1016/s0377-8401(01)00232-2.

[vms370321-bib-0182] Melnyk, J. P. , S. Wang , and M. F. Marcone . 2010. “Chemical and Biological Properties of the World's Most Expensive Spice: Saffron.” Food Research International 43, no. 8: 1981–1989. 10.1016/j.foodres.2010.07.033.

[vms370321-bib-0183] Mirheidari, A. , N. M. Torbatinejad , P. Shakeri , and A. Mokhtarpour . 2020. “Effects of Biochar Produced From Different Biomass Sources on Digestibility, Ruminal Fermentation, Microbial Protein Synthesis and Growth Performance of Male Lambs.” Small Ruminant Research 183: 106042. 10.1016/j.smallrumres.2019.106042.

[vms370321-bib-0184] Mnisi, C. M. , S. I. Kunene , N. N. Soko , C. F. Egbu , and V. Mlambo . 2024. “Oyster Mushroom Bioprocessing Enhances the Nutritional Value of Olive Pomace for Ruminants.” South African Journal of Animal Science 54: 226–235.

[vms370321-bib-0185] Mohammadabadi, T. , S. Amindavar , M. Chaji , and E. Direkvandi . 2023. “Dietary Supplementation of Olive Pomace in Lactating Buffaloes: Effects on Milk and Yogurt Composition and Fatty Acid Profile Toward Heart Health.” 10.21203/rs.3.rs-3252204/v1. PMC13484900 42611697

[vms370321-bib-0186] Mokhtarpour, A. , A. A. Naserian , A. M. Tahmasbi , and R. Valizadeh . 2012. “Effect of Feeding Pistachio By‐Products Silage Supplemented With Polyethylene Glycol and Urea on Holstein Dairy Cows Performance in Early Lactation.” Livestock Science 148, no. 3: 208–213. 10.1016/j.livsci.2012.06.006.

[vms370321-bib-0187] Molosse, V. L. , G. L. Deolindo , R. V. Lago , et al. 2024. “The Use of Secondary Grape Biomass in Beef Cattle Nutrition on Carcass Characteristics, Quality and Shelf Life of Meat.” Food and Nutrition Sciences 15, no. 6: 447–469.

[vms370321-bib-0188] Molosse, V. L. , G. L. Deolindo , R. V. P. Lago , et al. 2023. “The Effects of the Inclusion of Ensiled and Dehydrated Grape Pomace in Beef Cattle Diet: Growth Performance, Health, and Economic Viability.” Animal Feed Science and Technology 302: 115671. 10.1016/j.anifeedsci.2023.115671.

[vms370321-bib-0189] Monteiro, C. S. , I. A. Adedara , E. O. Farombi , and T. Emanuelli . 2024. “Nutraceutical Potential of Olive Pomace: Insights From Cell‐Based and Clinical Studies.” Journal of the Science of Food and Agriculture 104, no. 7: 3807–3815. 10.1002/jsfa.13281. 38270195

[vms370321-bib-0190] Moustafa Omar, S. M. 2023. “Study the Possible Protective Effect of Saffron Extract on Induced Rats Toxicity by Acrylamide.” Egyptian Journal of Nutrition and Health 17, no. 2: 51–69. 10.21608/ejnh.2023.283072.

[vms370321-bib-0191] Najafi, Z. , H. A. Zahran , N. Şahin Yeşilçubuk , and H. Gürbüz . 2022. “Effect of Different Extraction Methods on Saffron Antioxidant Activity, Total Phenolic and Crocin Contents and the Protective Effect of Saffron Extract on the Oxidative Stability of Common Vegetable Oils.” Grasas y Aceites 73, no. 4: e480. 10.3989/gya.0783211.

[vms370321-bib-0192] Natalello, A. , G. Luciano , L. Morbidini , et al. 2019. “Effect of Feeding Pomegranate Byproduct on Fatty Acid Composition of Ruminal Digesta, Liver, and Muscle in Lambs.” Journal of Agricultural and Food Chemistry 67, no. 16: 4472–4482. 10.1021/acs.jafc.9b00307. 30929432

[vms370321-bib-0193] Natalello, A. , R. Menci , G. Luciano , et al. 2023. “Effect of Dietary Pomegranate By‐Product on Lamb Flavour.” Meat Science 198: 109118. 10.1016/j.meatsci.2023.109118. 36681062

[vms370321-bib-0194] Natalello, A. , A. Priolo , B. Valenti , et al. 2020. “Dietary Pomegranate By‐Product Improves Oxidative Stability of Lamb Meat.” Meat Science 162: 108037. 10.1016/j.meatsci.2019.108037. 31901579

[vms370321-bib-0195] Nath, P. C. , A. Ojha , S. Debnath , et al. 2023. “Valorization of Food Waste as Animal Feed: A Step Towards Sustainable Food Waste Management and Circular Bioeconomy.” Animals 13, no. 8: 1366. 10.3390/ani13081366. 37106930 PMC10134991

[vms370321-bib-0196] Nemati, Z. , S. Amirdahri , A. Asgari , et al. 2024. “Feeding Pomegranate Pulp to Ghezel Lambs for Enhanced Productivity and Meat Quality.” Veterinary and Animal Science 24: 100356. 10.1016/j.vas.2024.100356. 38774584 PMC11106540

[vms370321-bib-0197] Neofytou, M. C. , A.‐L. Hager‐Theodorides , E. Sfakianaki , et al. 2023. “The Dietary Inclusion of Ensiled Olive Cake Increases Unsaturated Lipids in Milk and Alters the Expression of Lipogenic Genes in Mammary and Adipose Tissue in Goats.” Animals 13, no. 21: 3418. 10.3390/ani13213418. 37958173 PMC10650401

[vms370321-bib-0198] Niu, P. , M. Kreuzer , A. Liesegang , C. Kunz , A. Schwarm , and K. Giller . 2023. “Effects of Graded Levels of Dietary Pomegranate Peel on Methane and Nitrogen Losses, and Metabolic and Health Indicators in Dairy Cows.” Journal of Dairy Science 106, no. 12: 8627–8641. 10.3168/jds.2022-23141. 37641245

[vms370321-bib-0199] Noruzi, H. , F. Aziz‐Aliabadi , and Z. K. Imari . 2024. “Effects of Different Levels of Pistachio (*Pistachia vera*) Green Hull Aqueous Extract on Performance, Intestinal Morphology and Antioxidant Capacity in *Eimeria* Challenged Broilers.” Poultry Science 103, no. 6: 103667. 10.1016/j.psj.2024.103667. PMC11004999 38574462

[vms370321-bib-0200] Oancea, A.‐G. , M. Saracila , P. A. Vlaicu , I. Varzaru , A. E. Untea , and C. Dragomir . 2024. “Assessment of the Antioxidant Potential of Blackthorns and Hawthorns: Comparative Analysis and Potential Use in Ruminants Nutrition.” Separations 11, no. 9: 275. 10.3390/separations11090275.

[vms370321-bib-0201] Obeidat, B. S. 2023. “Effect of Feeding Pomegranate Seed Pulp on Awassi Lambs' Nutrient Digestibility, Growth Performance, and Carcass Quality.” Veterinary World 16, no. 3: 588–594. 10.14202/vetworld.2023.588-594. 37041845 PMC10082746

[vms370321-bib-0202] Obeidat, B. S. , M. H. Qadorah , and M. G. Thomas . 2024. “Effects of Feeding Pomegranate Seed Pulp and Coconut Meal By‐Products on Milk Yield, Milk Quality, and Metabolic Responses of Awassi Ewes and Pre‐Weaning Growth.” Veterinary World 17, no. 5: 1149–1156. 10.14202/vetworld.2024.1149-1156. 38911096 PMC11188889

[vms370321-bib-0203] Okasha, M. , R. Hegazy , and R. M. Kamel . 2023. “Assessment of Raisins Byproducts for Environmentally Sustainable Use and Value Addition.” AgriEngineering 5, no. 3: 1469–1480. 10.3390/agriengineering5030091.

[vms370321-bib-0204] Omidi, A. , and H. A. Nik . 2023. “Altered Body Weight Gain and Selected Blood Constituents of Baluchi Lambs Fed Dried Pomegranate Peels Supplemented Ration.” Comparative Clinical Pathology 33, no. 2: 231–237. 10.1007/s00580-023-03544-4.

[vms370321-bib-0205] Osman, A. , A. O. Alameen , W. a. Elmagbol , et al. 2024. “Impact of Methanolic Extract of Pomegranate (*Punica granatum* L.) Seeds on Serum Biomarkers in Wistar Rats Fed High Cholesterol and Fructose Diet.” Egyptian Journal of Veterinary Sciences 1–10. 10.21608/ejvs.2024.292047.2115.

[vms370321-bib-0206] El Otmani, S. , M. Chentouf , J. L. Hornick , and J. F. Cabaraux . 2019. “Chemical Composition and *In Vitro* Digestibility of Alternative Feed Resources for Ruminants in Mediterranean Climates: Olive Cake and Cactus Cladodes.” Journal of Agricultural Science 157, no. 03: 260–271. 10.1017/s0021859619000558.

[vms370321-bib-0207] Özdikicierler, O. , and B. Öztürk‐Kerimoğlu . 2023. Bioactive Phytochemicals From Pistachio (Pistachia vera L.) Oil Processing By‐Products, 577–594. Springer International Publishing. 10.1007/978-3-030-91381-6_27.

[vms370321-bib-0208] Paié‐Ribeiro, J. , F. Baptista , J. Teixeira , et al. 2024. “From Waste to Resource: Compositional Analysis of Olive Cake's Fatty Acids Nutrients and Antinutrients.” Applied Sciences 14, no. 13: 5586. 10.3390/app14135586.

[vms370321-bib-0209] Palmonari, A. , D. Cavallini , C. J. Sniffen , et al. 2021. “ *In Vitro* Evaluation of Sugar Digestibility in Molasses.” Italian Journal of Animal Science 20, no. 1: 571–577. 10.1080/1828051X.2021.1899063.

[vms370321-bib-0210] Pathak, P. D. , S. A. Mandavgane , and B. D. Kulkarni . 2016. “Valorization of Pomegranate Peels: A Biorefinery Approach.” Waste and Biomass Valorization 8, no. 4: 1127–1137. 10.1007/s12649-016-9668-0.

[vms370321-bib-0211] Pauletto, M. , R. Elgendy , A. Ianni , et al. 2020. “Nutrigenomic Effects of Long‐Term Grape Pomace Supplementation in Dairy Cows.” Animals 10, no. 4: 714. 10.3390/ani10040714. 32325906 PMC7222749

[vms370321-bib-0212] Peixoto, C. M. , M. I. Dias , M. J. Alves , et al. 2018. “Grape Pomace as a Source of Phenolic Compounds and Diverse Bioactive Properties.” Food Chemistry 253: 132–138. 10.1016/j.foodchem.2018.01.163. 29502813

[vms370321-bib-0213] Pinotti, L. , A. Luciano , M. Ottoboni , et al. 2021. “Recycling Food Leftovers in Feed as Opportunity to Increase the Sustainability of Livestock Production.” Journal of Cleaner Production 294: 126290. 10.1016/j.jclepro.2021.126290.

[vms370321-bib-0214] Pinotti, L. , S. Mazzoleni , A. Moradei , P. Lin , and A. Luciano . 2023. “Effects of Alternative Feed Ingredients on Red Meat Quality: A Review of Algae, Insects, Agro‐Industrial By‐Products and Former Food Products.” Italian Journal of Animal Science 22, no. 1: 695–710. 10.1080/1828051X.2023.2238784.

[vms370321-bib-0215] Popović‐Djordjević, J. B. , A. Ž. Kostić , and M. Kiralan . 2021. “Antioxidant Activities of Bioactive Compounds and Various Extracts Obtained From Saffron.” In Saffron, 41–97. Academic Press. 10.1016/b978-0-12-821219-6.00002-6.

[vms370321-bib-0216] Potortì, A. G. , V. Lopreiato , V. Nava , et al. 2024. “The Use of Olive Cake in the Diet of Dairy Cows Improves the Mineral Elements of Provola Cheese.” Food Chemistry 436: 137713. 10.1016/j.foodchem.2023.137713. 37857194

[vms370321-bib-0217] Proca, A. C. , L. Horodincu , C. Solcan , and G. Solcan . 2024. “The Potential of Grape Polyphenols Additive in Pig Nutrition: Chemical Structure, Bioavailability and Their Effect on Intestinal Health of Pigs.” Agriculture 14, no. 7: 1142. 10.3390/agriculture14071142.

[vms370321-bib-0218] Quagliardi, M. , E. Frapiccini , M. Marini , et al. 2024. “Use of Grape By‐Products in Aquaculture: New Frontiers for a Circular Economy Application.” Heliyon 10, no. 5: e27443. 10.1016/j.heliyon.2024.e27443. 38468965 PMC10926132

[vms370321-bib-0219] Quideau, S. , D. Deffieux , C. Douat‐Casassus , and L. Pouységu . 2011. “Plant Polyphenols: Chemical Properties, Biological Activities, and Synthesis.” Angewandte Chemie International Edition 50, no. 3: 586–621. 10.1002/anie.201000044. 21226137

[vms370321-bib-0220] Rabee, A. E. , K. Z. Kewan , E. A. Sabra , H. M. El Shaer , and M. Lamara . 2021. “Rumen Bacterial Community Profile and Fermentation in Barki Sheep Fed Olive Cake and Date Palm Byproducts.” PeerJ 9: e12447. 10.7717/peerj.12447. 34820187 PMC8605757

[vms370321-bib-0221] Rafiee, Z. , M. Barzegar , M. A. Sahari , and B. Maherani . 2018. “Nanoliposomes Containing Pistachio Green Hull's Phenolic Compounds as Natural Bio‐Preservatives for Mayonnaise.” European Journal of Lipid Science and Technology 120, no. 9: 1800086. 10.1002/ejlt.201800086.

[vms370321-bib-0222] Rashed‐Mohassel, M.‐H. 2020. “Evolution and Botany of Saffron (*Crocus sativus* L.) and Allied Species.” In Woodhead Publishing Series in Food Science, Technology and Nutrition, edited by A. Koocheki and M. B. T.‐S. Khajeh‐Hosseini , 37–57. Woodhead Publishing. 10.1016/B978-0-12-818638-1.00004-6.

[vms370321-bib-0223] Rebolledo‐Leiva, R. , D. Hernández , M. T. Moreira , and S. González‐García . 2024. “Using Olive and Apple Pomaces for Fattening Pig Diets: Environmental Impacts Under an Attributional and Consequential Perspective.” Environmental Technology & Innovation 34: 103549. 10.1016/j.eti.2024.103549.

[vms370321-bib-0224] Renna, M. , A. L. Martínez Marín , C. Lussiana , L. Colonna , A. Mimosi , and P. Cornale . 2023. “Caprine Milk Fatty Acid Responses to Dietary Dried Grape Pomace.” Italian Journal of Animal Science 22, no. 1: 1186–1194. 10.1080/1828051x.2023.2276259.

[vms370321-bib-0225] Rezaee Khorasany, A. , and H. Hosseinzadeh . 2016. “Therapeutic Effects of Saffron (*Crocus sativus* L.) in Digestive Disorders: A Review.” Iranian Journal of Basic Medical Sciences 19, no. 5: 455–469. 27403251 PMC4923465

[vms370321-bib-0226] Rezaeipour, V. , B. Dastar , A. F. B. Yaghobfar , and A. Gheisari . 2012. “Effects of Dietary Dried Tomato Pomace With an Exogenous Enzyme Supplementation on Growth Performance, Meat Oxidative Stability and Nutrient Digestibility of Broiler.” Journal of Animal Science Advances 2, no. 9: 777–786.

[vms370321-bib-0227] Ripari Garrido, J. , M. Patrignani , M. C. Puppo , and M. V. Salinas . 2024. “Nutritional and Bioactive Characterization of Pistachio—A Review With Special Focus on Health.” Exploration of Foods and Foodomics 2, no. 4: 363–390. 10.37349/eff.2024.00042.

[vms370321-bib-0228] Rolinec, M. , D. Bíro , M. Šimko , et al. 2021. “Grape Pomace Ingestion by Dry Cows Does Not Affect the Colostrum Nutrient and Fatty Acid Composition.” Animals 11, no. 6: 1633. 10.3390/ani11061633. 34073000 PMC8227017

[vms370321-bib-0229] Rolinec, M. , J. Medo , M. Gábor , et al. 2023. “Effect of Grape Pomace Intake on the Rumen Bacterial Community of Sheep.” Diversity 15, no. 2: 234. 10.3390/d15020234.

[vms370321-bib-0230] Romelle Jones, K. , S. Karuppusamy , and V. Sundaram . 2024. “Unraveling the Promise of Agroindustrial Byproducts as Alternative Feed Source for Sustainable Rabbit Meat Production.” Emerging Animal Species 10: 100044. 10.1016/j.eas.2024.100044.

[vms370321-bib-0231] Sadeghi, M. , E. Ghasemi , R. Sadeghi , et al. 2024. “Productivity and Nitrogen Metabolism of Lactating Cows Fed Pistachio Hull With Soybean Meal Partially Replaced by Slow‐Release Urea.” Tropical Animal Health and Production 56, no. 8: 305. 10.1007/s11250-024-04123-7. 39347824

[vms370321-bib-0232] Safarzadeh Markhali, F. 2021. “Effect of Processing on Phenolic Composition of Olive Oil Products and Olive Mill By‐Products and Possibilities for Enhancement of Sustainable Processes.” Processes 9, no. 6: 953. 10.3390/pr9060953.

[vms370321-bib-0233] Salami, S. A. , G. Luciano , M. N. O'Grady , et al. 2019. “Sustainability of Feeding Plant By‐Products: A Review of the Implications for Ruminant Meat Production.” Animal Feed Science and Technology 251: 37–55. 10.1016/j.anifeedsci.2019.02.006.

[vms370321-bib-0234] Salas‐Millán, J. Á. , and E. Aguayo . 2024. “Fermentation for Revalorisation of Fruit and Vegetable By‐Products: A Sustainable Approach Towards Minimising Food Loss and Waste.” Foods 13, no. 22: 3680. 10.3390/foods13223680. 39594095 PMC11594132

[vms370321-bib-0235] San Martin, D. , J. Ibarruri , M. Gutierrez , et al. 2024. “Valorisation of Grape Stem as an Alternative Ingredient in Rabbit Feed.” Waste Management & Research: The Journal for a Sustainable Circular Economy 20: 734242X241259660. 10.1177/0734242x241259660. 38902937

[vms370321-bib-0236] Sánchez‐Terrón, G. , R. Martínez , D. Morcuende , V. Caballero , and M. Estévez . 2024. “Pomegranate Supplementation Alleviates Dyslipidemia and the Onset of Non‐Alcoholic Fatty Liver Disease in Wistar Rats by Shifting Microbiota and Producing Urolithin‐Like Microbial Metabolites.” Food & Function 15, no. 14: 7348–7363. 10.1039/D4FO00688G. 38661445

[vms370321-bib-0237] Saremi, V. , D. Alipour , A. Azarfar , and R. Sedighi . 2014. “Effect of Different Levels of Raisin Waste on Performance, Nutrients Digestibility and Protozoal Population of Mehraban Growing Lambs.” Spanish Journal of Agricultural Research 12, no. 1: 159–166. 10.5424/sjar/2014121-4613.

[vms370321-bib-0238] Sargın, H. G. , and N. Denek . 2017. “Effect of Adding Different Levels of Dried Molasses Sugar Beet Pulp on the Silage Quality and *In Vitro* Digestibility of Wet Tomato Pomace Silage.” Harran Üniversitesi Veteriner Fakültesi Dergisi 6, no. 1: 84–89.

[vms370321-bib-0239] Schuster, M. J. , X. Wang , T. Hawkins , and J. E. Painter . 2017. “A Comprehensive Review of Raisins and Raisin Components and Their Relationship to Human Health.” Journal of Nutrition and Health 50, no. 3: 203. 10.4163/jnh.2017.50.3.203.

[vms370321-bib-0240] Scicutella, F. , M. A. Cucu , F. Mannelli , et al. 2023. “Rumen Microbial Community and Milk Quality in Holstein Lactating Cows Fed Olive Oil Pomace as Part in a Sustainable Feeding Strategy.” Animal 17, no. 6: 100815. 10.1016/j.animal.2023.100815. 37167820

[vms370321-bib-0241] Scicutella, F. , B. Valenti , A. Buccioni , et al. 2024. “Effect of Co‐Products From Olive‐Oil Production Chain on Rumen Microbial Communities: An *In Vitro* Study.” Italian Journal of Animal Science 23, no. 1: 532–545. 10.1080/1828051x.2024.2331560.

[vms370321-bib-0242] Seidali Dolat‐Abad, S. , H. Zare‐Maivan , and A. Ranjbar . 2016. “Effects of Bacterial Inoculants and Absorbents on Fermentation Properties and Chemical Composition of Fresh Sugar Beet Pulp Silage.” Iranian Journal of Animal Science Research 7, no. 4: 413–421.

[vms370321-bib-0243] Selim, N. A. , S. A. Nada , A. F. Abdel‐Sala , and S. F. Youssef . 2013. “Evaluation of Some Natural Antioxidant Sources in Broiler Diets: 2‐Effect on Chemical and Microbiological Quality of Chilled and Frozen Broiler Meat.” International Journal of Poultry Science 12, no. 10: 572–581. 10.3923/ijps.2013.572.581.

[vms370321-bib-0244] Shakerardekani, A. , and M. Molaei . 2020. “Post‐Harvest Pistachio Waste: Methods of Its Reduction and Conversion.” Pistachio and Health Journal 3, no. 2: 40–51.

[vms370321-bib-0245] Shakeri, P. 2016. “Pistachio By‐Product as an Alternative Forage Source for Male Lambs: Effects on Performance, Blood Metabolites, and Urine Characteristics.” Animal Feed Science and Technology 211: 92–99. 10.1016/j.anifeedsci.2015.11.011.

[vms370321-bib-0246] Shakeri, P. , A. Riasi , and M. Alikhani . 2014. “Effects of Long Period Feeding Pistachio By‐Product Silage on Chewing Activity, Nutrient Digestibility and Ruminal Fermentation Parameters of Holstein Male Calves.” Animal 8, no. 11: 1826–1831. 10.1017/s1751731114001621. 25322789

[vms370321-bib-0247] Shiri‐Shahsavar, M. R. , S. Alijani , N. Parsamanesh , et al. 2023. “The Effect of Grape‐Seed Oil on Diabetes‐Related Hyperglycemia, Dyslipidemia, and Inflammation in Streptozotocin‐Induced Diabetic Rats.” Obesity Medicine 37: 100476. 10.1016/j.obmed.2022.100476.

[vms370321-bib-0248] Signor, M. H. , A. L. de Freitas dos Santos , M. G. de Vitt , et al. 2024. “Grape Seed Oil in the Diet of Primiparous Jersey Cows Before and After Parturition: Effects on Performance, Health, Rumen Environment, and Milk Quality.” Tropical Animal Health and Production 56, no. 6: 202. 10.1007/s11250-024-04064-1. 38992295

[vms370321-bib-0249] Sinthuja, S. , S. Ramachandra , and A. U. Alahakoon . 2023. “Importance of Nutritional Properties of Fruit and Vegetable Wastes and By‐Products in Livestock Feed Production: A Review.” In 2023 International Research Conference of Sri Lanka Technology Campus Colombo, Sri Lanka, 245–249.

[vms370321-bib-0250] Smaoui, S. , H. B. Hlima , A. C. Mtibaa , et al. 2019. “Pomegranate Peel as Phenolic Compounds Source: Advanced Analytical Strategies and Practical Use in Meat Products.” Meat Science 158: 107914. 10.1016/j.meatsci.2019.107914. 31437671

[vms370321-bib-0251] Sokač Cvetnić, T. , V. Gunjević , A. Damjanović , et al. 2023. “Monitoring of Chemical and Fermentative Characteristics During Different Treatments of Grape Pomace Silage.” Agriculture 13, no. 12: 2264. 10.3390/agriculture13122264.

[vms370321-bib-0252] SoltaniNezhad, B. , O. Dayani , A. Khezri , and R. Tahmasbi . 2016. “Performance and Carcass Characteristics in Fattening Lambs Feed Diets With Different Levels of Pistachio By‐Products Silage With Wasted Date.” Small Ruminant Research 137: 177–182. 10.1016/j.smallrumres.2016.03.015.

[vms370321-bib-0253] Symeou, S. , D. Miltiadou , C. Constantinou , P. Papademas , and O. Tzamaloukas . 2021. “Feeding Olive Cake Silage up to 20% of DM Intake in Sheep Improves Lipid Quality and Health‐Related Indices of Milk and Ovine Halloumi Cheese.” Tropical Animal Health and Production 53: 1–7. 10.1007/s11250-021-02674-7. 33772370

[vms370321-bib-0254] Taheri, M. R. , M. J. Zamiri , E. Rowghani , and A. Akhlaghi . 2012. “Effect of Feeding Olive‐Pulp Ensiled With Additives on Feedlot Performance and Carcass Attributes of Fat‐Tailed Lambs.” Tropical Animal Health and Production 45: 345–350. 10.1007/s11250-014-0555-z. 22820912

[vms370321-bib-0255] Tahmasbi, R. , H. Moghadam , A. Naserian , and B. Saremi . 2004. “Chemical Composition of Mixed Corn Plant and Tomato Pomace Silage and Its Effect on Holstein Dairy Cattle Performance.” In New Dimensions and Challenges for Sustainable Livestock Farming: Proceedings of the 11th Animal Science Congress, Kuala Lumpur, Malaysia. Malaysian Society of Animal Production.

[vms370321-bib-0256] Tayengwa, T. , O. C. Chikwanha , M. E. R. Dugan , T. Mutsvangwa , and C. Mapiye . 2020. “Influence of Feeding Fruit By‐Products as Alternative Dietary Fibre Sources to Wheat Bran on Beef Production and Quality of Angus Steers.” Meat Science 161: 107969. 10.1016/j.meatsci.2019.107969. 31704473

[vms370321-bib-0257] Tayengwa, T. , O. C. Chikwanha , E. Raffrenato , M. E. R. Dugan , T. Mutsvangwa , and C. Mapiye . 2021. “Comparative Effects of Feeding Citrus Pulp and Grape Pomace on Nutrient Digestibility and Utilization in Steers.” Animal 15, no. 1: 100020. 10.1016/j.animal.2020.100020. 33516014

[vms370321-bib-0258] Teixeira, A. , N. Baenas , R. Dominguez‐Perles , et al. 2014. “Natural Bioactive Compounds From Winery By‐Products as Health Promoters: A Review.” International Journal of Molecular Sciences 15, no. 9: 15638–15678. 10.3390/ijms150915638. 25192288 PMC4200838

[vms370321-bib-0259] Teng, Z. , S. Liu , L. Zhang , et al. 2024. “Tea Polyphenols Inhibit Methanogenesis and Improve Rumen Epithelial Transport in Dairy Cows.” Animals 14, no. 17: 2569. 10.3390/ani14172569. 39272354 PMC11394105

[vms370321-bib-0260] Thaore, V. , M. Bahramian , M. Boudou , P. Hynds , and A. Priyadarshini . 2024. “Geospatial Analysis of Food Waste Generation at the Consumer‐Level in High‐Income Regions, 2000–2023–A Scoping Review.” Environmental Research 263: 120247. 10.1016/j.envres.2024.120247. 39476926

[vms370321-bib-0261] Thiruchelvi, R. , P. Jayashree , G. A. Nayik , et al. 2020. “Raisin.” In Antioxidants in Vegetables and Nuts – Properties and Health Benefits, 523–537. Springer Singapore. 10.1007/978-981-15-7470-2_27.

[vms370321-bib-0262] Tripura, S. , K. Shyama , K. Ally , et al. 2021. “Incorporation of Cooked Barley Residue and Spent Grapes in the Ration of Pregnant Large White Yorkshire Sows and Their Piglets.” Tropical Animal Health and Production 53, no. 77: 1–12. 10.1007/s11250-020-02504-2. 33404863

[vms370321-bib-0263] Tuoxunjiang, H. , A. Yimamu , X. Li , R. Maimaiti , and Y. Wang . 2020. “Effect of Ensiled Tomato Pomace on Performance and Antioxidant Status in the Peripartum Dairy Cow.” Journal of Animal and Feed Sciences 29, no. 2: 105–114. 10.22358/jafs/124049/2020.

[vms370321-bib-0264] Turcu, R. P. , T. D. Panaite , A. E. Untea , P. A. Vlaicu , I. A. Badea , and S. Mironeasa . 2021. “Effects of Grape Seed Oil Supplementation to Broilers Diets on Growth Performance, Meat Fatty Acids, Health Lipid Indices and Lipid Oxidation Parameters.” Agriculture 11, no. 5: 404. 10.3390/agriculture11050404.

[vms370321-bib-0265] Vakili, R. , and A. Mokhtarpour . 2023. Laying Performance, Egg Composition, Blood Metabolites, Fecal Minerals Excretion, Fecal pH and Ammonia Emission in Laying Hens Fed Feeding with Different Saffron Extract Supplementation Levels . SSRN. 10.2139/ssrn.4328187. [preprint].

[vms370321-bib-0266] Valizadeh, R. , A. A. Naserian , S. Ghasemi , and M. A. Mousavi . 2019. “The Effects Gamma Irradiation, Sodium Hydroxide, Urea and Polyethylene Glycol on Phenolic Compounds, *In Vitro* Gas Production Kinetics and Microbial Protein Synthesis of Pistachio By‐Products.” Journal of Ruminant Research 7, no. 2: 97–112.

[vms370321-bib-0267] Vastolo, A. , F. Serrapica , D. Cavallini , I. Fusaro , A. S. Atzori , and M. Todaro . 2024. “Editorial: Alternative and Novel Livestock Feed: Reducing Environmental Impact.” Frontiers in Veterinary Science 11: 1441905. 10.3389/fvets.2024.1441905. 39100757 PMC11295273

[vms370321-bib-0268] Viveros, A. , S. Chamorro , M. Pizarro , I. Arija , C. Centeno , and A. Brenes . 2011. “Effects of Dietary Polyphenol‐Rich Grape Products on Intestinal Microflora and Gut Morphology in Broiler Chicks.” Poultry Science 90, no. 3: 566–578. 10.3382/ps.2010-00889. 21325227

[vms370321-bib-0269] Voicu, D. , I. Voicu , A. Vasilachi , R. Uta , T. Mihalcea , and R. S. Pelmus . 2016. “Influence of Sorghum Inclusion in Fattening Steers Diets on Health and Fatty Acids Profile of *Longissimus dorsi* Muscle.” Indian Journal of Animal Sciences 86, no. 7: 777–780. 10.56093/ijans.v86i7.59921.

[vms370321-bib-0270] Wang, R. , H. Yu , H. Fang , et al. 2020. “Effects of Dietary Grape Pomace on the Intestinal Microbiota and Growth Performance of Weaned Piglets.” Archives of Animal Nutrition 74, no. 4: 296–308. 10.1080/1745039x.2020.1743607. 32308036

[vms370321-bib-0271] Xu, P. , J. Wang , P. Chen , et al. 2024. “Effects of Pomegranate (*Punica granatum* L.) Peel on the Growth Performance and Intestinal Microbiota of Broilers Challenged With *Escherichia coli* .” Poultry Science 103, no. 2: 103304. 10.1016/j.psj.2023.103304. PMC10757254 38096668

[vms370321-bib-0272] Yang, F. , W. Yanping , S. Zhao , and W. Yuan . 2020. “ *Lactobacillus Plantarum* Inoculants Delay Spoilage of High Moisture Alfalfa Silages by Regulating Bacterial Community Composition.” Frontiers in Microbiology 11: 1989. 10.3389/fmicb.2020.01989. 32903392 PMC7434841

[vms370321-bib-0273] Yang, K. , Y. Qing , Q. Yu , et al. 2021. “By‐Product Feeds: Current Understanding and Future Perspectives.” Agriculture 11, no. 3: 207. 10.3390/agriculture11030207.

[vms370321-bib-0274] Yari, M. , M. Manafi , M. Hedayati , et al. 2015a. “Nutritive Value of Several Raisin By‐Products for Ruminants Evaluated by Chemical Analysis and *In Situ* Ruminal Degradability.” Research Opinions in Animal and Veterinary Sciences 5, no. 4: 198–204.

[vms370321-bib-0275] Yari, M. , M. Manafi , M. Hedayati , et al. 2015b. “Prediction of Energy Contents and Potential Nutrient Supply of Raisin Byproducts for Ruminants Using National Research Council Feeding System and *In Vitro* Gas Production Method.” Research Opinions in Animal and Veterinary Sciences 5, no. 6: 284–289.

[vms370321-bib-0276] Yari, M. , S. Valinejad , M. Manafi , Z. Ghaseminejad , A. Koolivand , and S. M. Zolhavarieh . 2016. “Nutritional Value of Raisin By‐Product and Its Effect on Late Flower Alfalfa Hay Fermentation Profile and Nutrient Availability in Ruminants.” Iranian Journal of Animal Science Research 8, no. 2: 271–283.

[vms370321-bib-0277] Yu, D. , W. Zhao , J. Dong , et al. 2022. “Multifunctional Bioactive Coatings Based on Water‐Soluble Chitosan With Pomegranate Peel Extract for Fish Flesh Preservation.” Food Chemistry 374: 131619. 10.1016/j.foodchem.2021.131619. 34810018

[vms370321-bib-0278] Zhao, Y. , J. Guo , Y. Zhang , X. Wang , R. Wang , and J. Li . 2012. “Effects of Tomato Pomace Fermentation Feed on Growth Performance, Milk Composition and Blood Cell Parameters for Xinjiang Brown Cows.” Xinjiang Agricultural Sciences 49: 1546–1551.

